# Saving collections: taxonomic revision of the herpetological collection of the Instituto de Investigação Científica Tropical, Lisbon (Portugal) with a protocol to rescue abandoned collections

**DOI:** 10.3897/zookeys.1052.64607

**Published:** 2021-07-30

**Authors:** Luis M.P. Ceríaco, Diogo Parrinha, Mariana P. Marques

**Affiliations:** 1 Museu de História Natural e da Ciência da Universidade do Porto, Praça Gomes Teixeira 4099-002 Porto, Portugal Universidade de Lisboa Lisboa Portugal; 2 Departamento de Zoologia e Antropologia (Museu Bocage), Museu Nacional de História Natural e da Ciência, Universidade de Lisboa, Rua da Escola Politécnica, 58, 1269-102 Lisboa, Portugal Museu de História Natural e da Ciência da Universidade do Porto Porto Portugal; 3 Centro de Investigação em Biodiversidade e Recursos Genéticos (CIBIO), InBIO, Universidade do Porto, Rua Padre Armando Quintas 7, Vairão, 4485-661 Porto, Portugal Universidade do Porto Porto Portugal

**Keywords:** Angola, Cabo Verde, East Timor, Goa, Guinea-Bissau, herpetofauna, Macau, Mozambique, Natural History Collections, São Tomé & Príncipe

## Abstract

The herpetological collections of the Instituto de Investigação Científica Tropical, Lisbon, are amongst the most important collections from the former Portuguese territories in Africa and Asia. The collection comprises more than 5000 preserved specimens, including type specimens of nine taxa, *Trachylepis adamastor*, *Trachypelis thomensis*, *Panaspis thomensis*, *Naja peroescobari*, *Dalophia angolensis*, *Hemidactylus nzingae*, *Boaedon fradei*, *Platysaurus maculatus maculatus*, and *Platysaurus maculatus lineicauda*. The collection was abandoned in the early years of 2000s and was at risk of being lost. In this paper the entire collection is reviewed, a catalogue provided of the extant specimens, and a brief account of the history of herpetological research at IICT given. Details are also provided on the recovery of the collection and a protocol to rescue abandoned collections.

## Introduction

The zoological collections of the Instituto de Investigação Científica Tropical (IICT), Lisbon (Portugal), are amongst the largest and most important biological collections in Portugal. Spanning all major zoological groups, the IICT collections are mostly focused on the fauna of the former Portuguese colonial territories in Africa and Asia. The collection’s geographical coverage is of interest, as they cover areas from where collections are relatively scarce and countries for which the current faunal knowledge is still deficient. While part of the IICT zoological collections have been catalogued and digitised recently (see Monteiro et al. 2014, 2016, 2017), a considerable part was neglected for years. This was the case of the herpetological collections. With approximately 5000 specimens from Angola, Mozambique, Guinea-Bissau, Cabo Verde, São Tomé & Príncipe, East Timor, Macau (China), Goa (Portuguese India), and Portugal, the IICT herpetological collections are the largest of their kind in the country. These collections are also amongst the largest available collections of amphibians and reptiles in the world for some of the countries covered (e.g., Guinea-Bissau, Cabo Verde). These collections were built from the 1930s to the 1990s, during several expeditions and field surveys conducted by IICT researchers and staff, but also by donations and contributions from several Portuguese colonial officers and landowners in these former territories.

Despite its importance, the IICT herpetological collections were abandoned in the late 1990s. Without a fully dedicated curator or collection manager, the collection became degraded and almost completely inaccessible for researchers. Besides the original field books and an incomplete manuscript catalogue, the collections were never fully catalogued or digitised. In 2015 we started a cataloguing and digitisation project for the IICT herpetological collections. This process was followed by a complete evaluation of the conservation status of the collections, the recovery of the specimens that were in critical condition, and the transfer of the entire collection to the Museu Nacional de História Natural e da Ciência, Lisbon. In this paper we provide a comprehensive review of the taxonomic diversity and importance of the IICT herpetological collections, report on the collection recuperation process, and propose a protocol to recover abandoned natural history collections.

## Brief history of the IICT and its herpetological collections

Dating back to the second half of the nineteenth century, IICT’s institutional history has been particularly complex, even in the bureaucracy-prone context of Portuguese scientific institutions. Its origins are related to the foundation of the ‘Comissão de Cartografia’ in 1883, but only in 1936 did the institution become more active, when it was renamed ‘Junta das Missões Geográficas e de Investigações Coloniais’. This would not be the last name change of the institution, as it would be renamed ‘Junta de Investigações do Ultramar’ in 1945, ‘Junta de Investigações Científicas do Ultramar’ in 1973, ‘Laboratório Nacional de Investigação Científica Tropical’ in 1979, and finally ‘Instituto de Investigação Científica Tropical’ in 1982 (Anonymous 1983). The reform of the institution in 1936 and the publication of the National Plan for the Scientific Occupation of the Portuguese Overseas in 1945 (Anonymous 1945) established the internal organisation of the institution, namely its departments and branches. Subsequently, the institution established a centre for zoological research, commonly known as “Centro de Zoologia” [also known by the acronym CZL – Centro de Zoologia de Lisboa, see Sabaj (2020)]. The Centro de Zoologia was housed in an adapted mansion in Rua da Junqueira 14, Alcântara neighborhood, near the Tagus river, Lisbon. This centre was the main entity responsible for conducting zoological research in the Portuguese overseas territories, building, and maintaining collections and providing zoological expertise to other fields related to the colonial enterprise.

**Figure 1.**
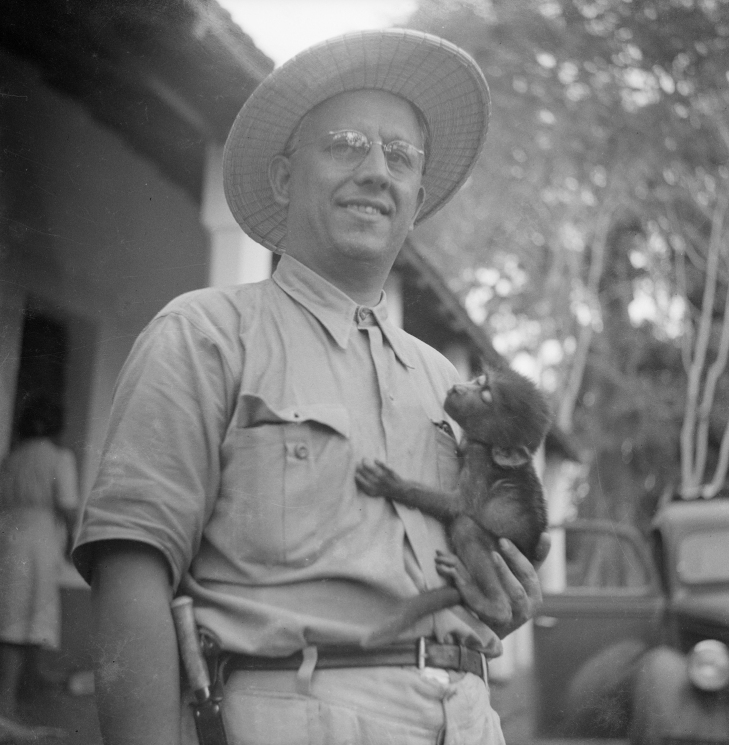
Fernando Frade during field work in Guinea-Bissau. Photograph credits: ULisboa – Col.Fotografia IICT-ZOO 22149.

The leadership of the Centro de Zoologia was appointed to the Portuguese zoologist Fernando Frade Viegas da Costa (1898–1983), commonly known as Fernando Frade (Fig. 1). Besides being the director of the CZL, Frade also had the responsibility for leading most of the so-called “Missões Zoológicas” (Zoological Missions) to the Portuguese overseas territories. The objective of these missions was to catalogue the fauna of those territories, study their ecological relationships, and understand the potential use or threats that native fauna could pose to the colonial enterprise (Anonymous 1945). During Frade’s direction the CZL led several zoological missions, namely to Guinea-Bissau, São Tomé & Príncipe, Cabo Verde, Angola, Mozambique, Portuguese India (Goa) and East Timor. Herpetological specimens were collected in the course of all these missions, as well as on other missions, as was the case for the Apiary Mission to Angola (1957–1959).

**Figure 2.**
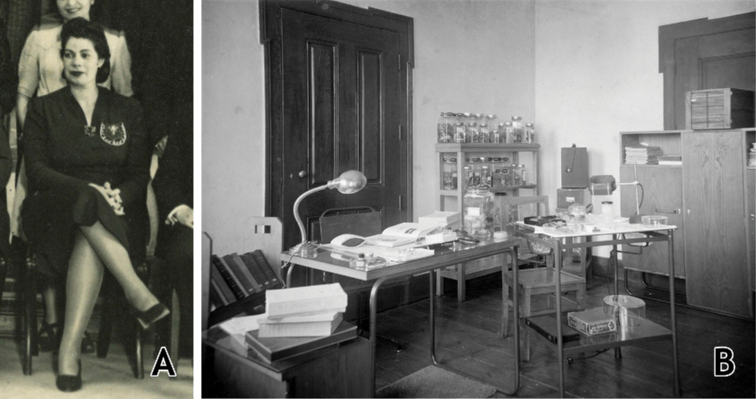
Portrait of Sara Manaças (**A**), and her herpetological laboratory (date unknown; **B**). Photograph credits: **A** courtesy of Luis Mendes **B** ULisboa – Col.Fotografia IICT-ZOO 21688.

**Figure 3.**
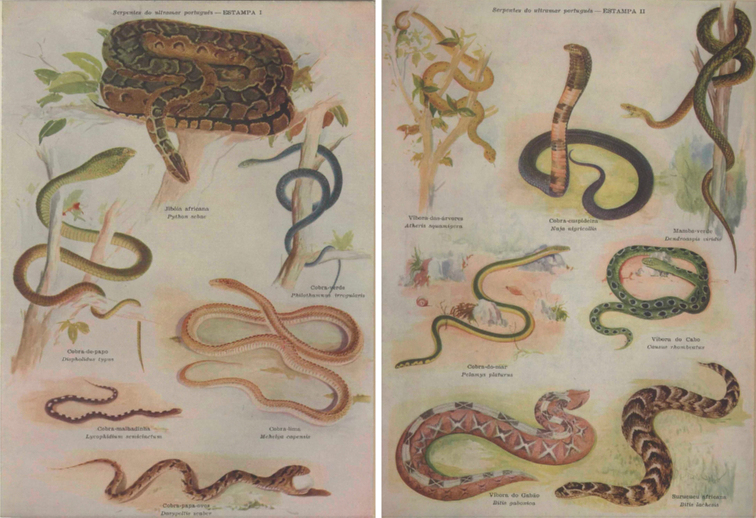
Plates with the water colours of the Portuguese painter António Silva Lino (1911–1984) for the poorly known manuscript “Serpentes do Ultramar Português”, co-authored by Fernando Frade and Sara Manaças (Frade and Manaças 1955).

The study of the herpetological collections was entrusted to the Portuguese herpetologist Sara Maria Bárbara Marques Manaças (1896–date of death unknown; Fig. 2). Manaças published 20 papers in which she identified and catalogued specimens, reported morphological data, and provided taxonomic and distribution comments. Although she worked with both amphibians and reptiles, the majority of her papers were focused on reptiles (7 vs. 13 publications, respectively). Her papers covered the herpetofauna of Guinea-Bissau (Manaças 1947, 1949, 1950a, 1951a, b, 1955, 1982), Mozambique (Manaças 1950b, 1952, 1954, 1959, 1961a, 1982), Portuguese Timor (currently East Timor) (Manaças 1956, 1972), São Tomé & Príncipe (Manaças 1958, 1973), Portuguese India – Goa (Manaças 1961b), and Angola (Manaças 1963, 1973 “1974”, 1982). All of these papers were single-authored by Manaças, with the exception of one, co-authored with Fernando Frade, on the venomous snakes of the Portuguese overseas territories (Frade and Manaças 1955; Fig. 3). The last paper of her career, also dedicated to the venomous snakes of Guinea-Bissau, São Tomé & Príncipe, Angola, and Mozambique (Manaças 1982), was published posthumously with some additions and corrections by the Portuguese herpetologist Margarida Pinheiro (date of birth unknown–to date). During the time Manaças curated the herpetological collections, the English-born Zimbabwean herpetologist Donald G. Broadley (1932–2016) visited and consulted the collections in August 1968 (Broadley 2018).

**Figure 4.**
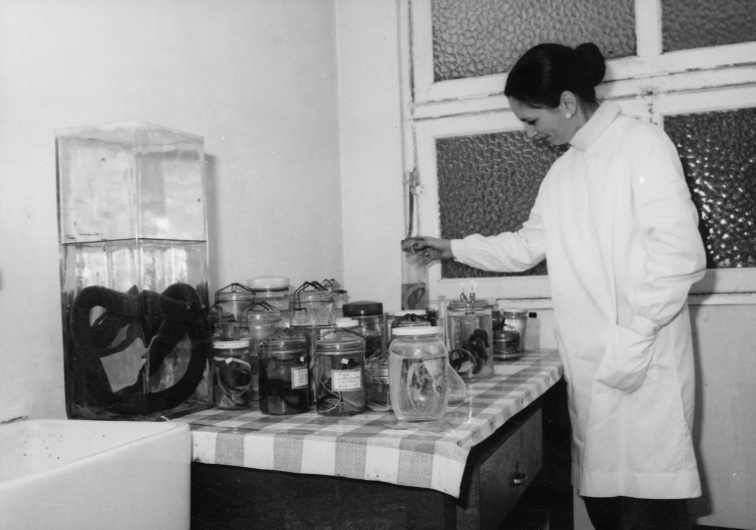
Margarida Pinheiro working with herpetological specimens in one of the laboratories of the Centro de Zoologia in 1979. Photograph credits: ULisboa – Col.Fotografia IICT 21199.

Following the passing of Manaças in the early 1980s, Pinheiro was entrusted with the herpetological collections (Fig. 4). With the independence of the former Portuguese territories (with the exception of Macau) in 1975, fieldwork almost halted, and the activity of the herpetology department slowed down considerably. Pinheiro focused mostly on the study of Cabo Verde’s herpetofauna (Pinheiro 1986, 1990), but also participated in a field survey to the then Portuguese territory of Macau (currently a special administrative region of the People’s Republic of China; Dias et al. 1994). From this latter survey, Pinheiro published a brief note on the distribution of the Agamid *Leiolepis belliana* (Hardwicke & Gray, 1827) in the region (Pinheiro 1994) and participated as a co-author, together with the herpetologist Clara Ruas (date of birth unknown–to date) and the entomologist Luís Mendes (1946–to date), on two notes on the diet of local amphibians (Mendes et al. 1994a, b). During the 1990’s, Clara Ruas became assistant researcher in the herpetological department. Ruas focused almost exclusively on the study of amphibians, and besides the aforementioned publications, she published two papers regarding the amphibians of Angola (Ruas 1996, 2002a) and one on the amphibians of Mozambique (Ruas 2002b).

**Figure 5.**
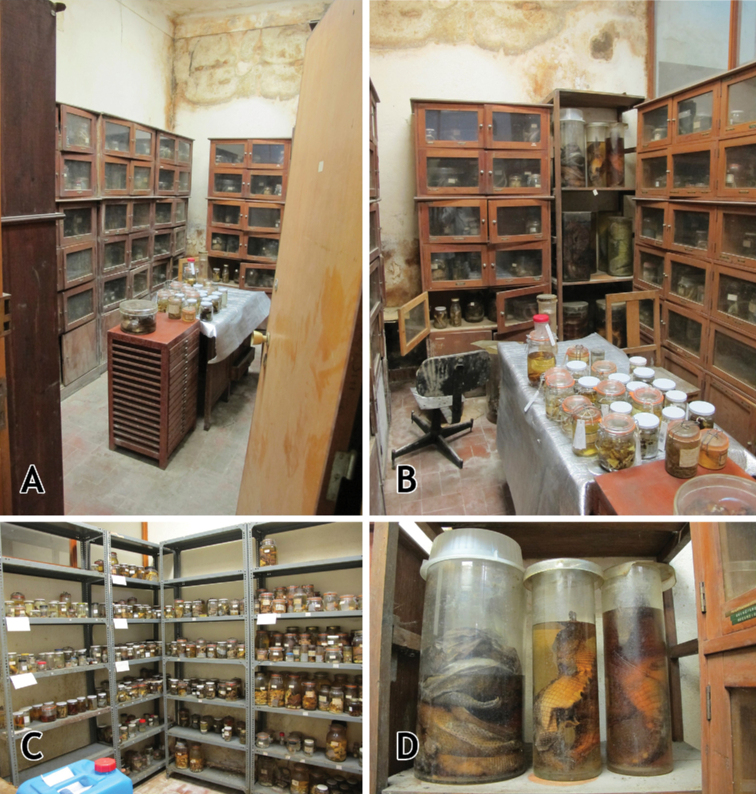
Herpetological collections reserve rooms in the basement of Centro de Zoologia, IICT, Lisbon, in July 2014 **A** entrance view of the room dedicated to the reptile collections, note the broken door **B** another view of the room dedicated to the reptile collections **C** view of the room dedicated to the amphibian collections **D** close-up of the poor conservation conditions of preserved specimens. Photographs by Luis M. P. Ceríaco.

During the first decades of the twenty-first century, the Centro de Zoologia became increasingly understaffed and research in the collections diminished considerably. This contributed to the degradation of the herpetological collections. Without constant supervision, lacking proper collection management, and housed in two rooms in the basement of the Centro de Zoologia building, which suffered from the lack of climate control, high humidity levels, and no security, the herpetological collections reached a critical situation and were at risk of being irreplaceably lost (Fig. 5).

After years of uncertainty regarding its future, IICT was formally closed by the Portuguese government on 31 July 2015 (Decreto-Lei 141/2015). With this closure, all the responsibilities of the institution, namely those of research and development, were transferred from the mansion in Rua da Junqueira to the University of Lisbon and its natural history collections were subsequently deposited in the Museu Nacional de História Natural e da Ciência (MUHNAC), Lisbon. The transfer of the collections from Centro de Zoologia to MUHNAC took place between 2015 and 2018. Despite sharing the same spaces and curatorial team, the IICT collections remain independent from the rest of MUHNAC collections, i.e., they maintain their former catalogue numbers and have not been catalogued as MUHNAC collections. The IICT herpetological collection is also considered a “closed” collection, in the sense that it is not accepting additional specimens, and therefore constitutes a closed set. In January 2018, the first author of this paper (LMPC) was appointed as External Curator of the IICT herpetological collections, while the third author (MPM) is the acting Assistant Curator.

## Materials and methods

**Figure 6.**
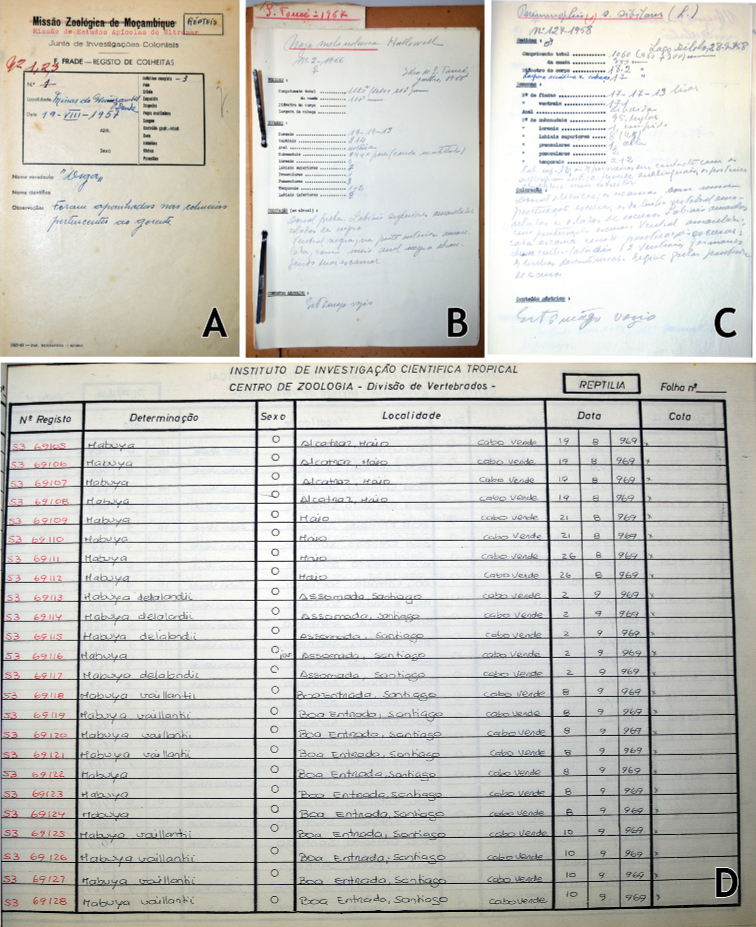
Documentation associated with the herpetological collection **A** field notebook of the Apiary Missions in Angola **B** data sheet associated with São Tomé snakes **C** data sheet associated with Angolan snakes **D** Manuscript catalogue of the herpetological collection. The “n° Registo” [record number] is not physically attached to the specimen, making it impossible to link the two. Photographs by Diogo Parrinha.

During an initial survey, all the available catalogues, field notebooks, and documentation associated with the collections were located, compiled, and digitised. The available field notebooks (one per major expedition), provided locality data, collecting dates, names of the collectors and/or identification of the expedition, and an assortment of natural history data and observations (Fig. 6A). Contrary to the catalogue numbers, the numbers in the field notebooks (i.e., the field numbers) were the same as those physically associated to the specimen. This number, comprising a serial number and the collecting year (e.g., 161/1959) was the same number used in all of the publications referable to the specimen, and therefore should be considered the *de facto* catalogue number. Manuscript documentation associated with the collection provided measurements, scale counts, and observations made by Sara Manaças for individual specimens (Fig. 6B, C). The available catalogue was organised by subcollection, with one entry per specimen, providing their taxonomic identity, collecting date, and locality (Fig. 6D). Each specimen also had a catalogue number, but this number was not physically associated with the specimen, nor was the respective field number, rendering it impossible to link the entry for the catalogue number and the specimen.

### List of institute acronyms (abbreviations follow Sabaj 2020)

**AMNH**American Museum of Natural History, New York, USA;

**BPBM**Bernice Pauahi Bishop Museum, Department of Zoology, Honolulu;

**CAS**California Academy of Sciences, San Francisco, USA;

**CM**Carnergie Museum, Pittsburgh, USA;

**CZL**Centro de Zoologia de Lisboa [extinct, now part of IICT, see below], Lisbon, Portugal;

**DiSSCO** Distribued System of Scientific Collections;

**GBIF** Global Biodiversity Information Facility;

**iDigBio** Integrated Digitized Biocollections;

**IICT** Instituto de Investigação Científica Tropical, Lisboa, Portugal;

**INBAC** Instituto Nacional da Biodiversidade e Áreas de Conservação, Luanda, Angola;

**ISCED** Instituto Superior de Ciências da Educação, Lubango, Angola;

**MCNB**Museu de Ciències Naturals de Barcelona, Barcelona, Spain;

**MCUC**Museu da Ciência da Universidade de Coimbra, Coimbra, Portugal;

**MD**Museu Regional do Dundo, Dundo, Angola;

**MCZ**Museum of Comparative Zoology, Harvard University, Cambridge, USA;

**MHNC**Musée d’Histoire Naturelle, la Chaux-de-Fonds, Switzerland;

**MHNC-UP**Museu de História Natural e da Ciência da Universidade do Porto, Porto, Portugal;

**MNHN**Muséum National d’Histoire Naturelle, Paris, France;

**MUHNAC**Museu Nacional de História Natural e da Ciência, Lisbon, Portugal;

**MVZ**Museum of Vertebrate Zoology, University of California, Berkeley, USA;

**NCSM**North Carolina Museum of Natural Sciences, Raleigh, USA;

**NHMUK**Natural History Museum, London, UK;

**PEM**Port Elizabeth Museum, Port Elizabeth, South Africa;

**TCWC** Biodiversity Research and Teaching Collections, Department of Wildlife and Fisheries Sciences, Texas A&M University, College Station, USA;

**TM**Ditsong National Museum of Natural History, Pretoria, South Africa;

**UMMZ**University of Michigan Museum of Zoology, Ann Arbor, USA;

**USNM**Smithsonian Institution, National Museum of Natural History;

**YPM**Yale University, Peabody Museum of Natural History, New Haven, USA;

**ZFMK**Zoologisches Forschlungmuseum Alexander Koenig, Bonn, Germany;

**ZSI** Zoological Society of India, India.

**Figure 7.**
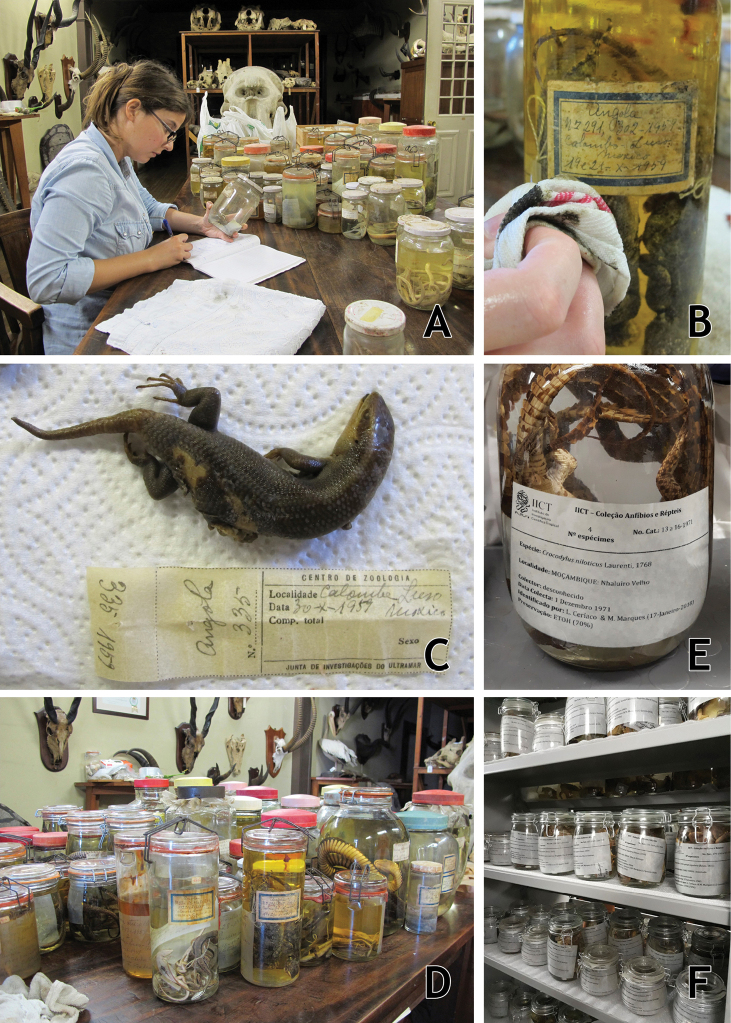
Rehabilitation and revision of the IICT herpetological collections **A** initial listing of the specimens to be transported **B** cleaning of the external label **C** example of internal label, usually attached to individual specimens **D** general view of the different types of jars and respective conservation issues associated–embrittled and or melted rubber gaskets, oxidized metal stoppers, cracked jars, etc **E** new standardized ResistAll internal label **F**IICT herpetology collection after the intervention in the MUHNAC reserves. Photographs by Luis M. P. Ceríaco.

All herpetological specimens were located in the CZL reserves, and after a brief initial identification and listing (Fig. 7A), were transported to the Wet Laboratory of MUHNAC. Specimens were distributed in different types of glass jars, with most of the specimens in glass-top, wire-bail jars with rubber gaskets, although some specimens were in glass jars with Bakelite or metal lids. None of the jars was in optimal condition, ranging from dirty to broken. Almost all the specimens were preserved in formaldehyde, as inferred based on the examination. Once in the laboratory, jars and other containers were cleaned in order to retrieve the available data from the existing external labels (Fig. 7B). These data were confirmed against the contents of the jar/container as well as with any existing specimen tags or internal labels (either attached to the specimens or in the bottom of the jar; Fig. 7C). Data on the jars and specimens were cross-referenced to data available in scientific publications that cited IICT specimens to confirm the presence of individual specimens in the collections and compare the published data to the data on the label/tags (Manaças 1947, 1949, 1950a, b, 1951a, b, 1952, 1954, 1955, 1956, 1958, 1959, 1961a, b, 1972, 1973, 1973 “1974”, 1982; Pinheiro 1986, 1990, 1994; Mendes et al. 1994a, b; Ruas 1996, 2002a, b; Ceríaco 2015; Ceríaco et al. 2016, 2017, 2020a, b, 2021; Soares et al. 2018; Hallermannn et al. 2020). All specimens, whenever possible, were identified to species level. For this we followed the most updated checklists, guides, and identification keys to the groups and geographical regions covered in the collection.

As the majority of the jars presented structural deficiencies (cracks, melted or embrittled gaskets, rusty/oxidised metal stoppers and/or lids, etc.; Fig. 7D) they were discarded. Original external labels were retrieved whenever possible. Following a similar procedure to that suggested by Simmons (2014), specimens that were originally preserved in formaldehyde (the vast majority) were rinsed in distilled water for a few minutes, then underwent steps of 20% increase in concentration of ethanol (20% for 30 minutes, 40% for 30 minutes, 60% for 30 minutes, 70% for final preservation). All the specimens were placed in 70% ethanol. In those cases in which the original jar/container had been discarded, specimens were placed in new jars. Original tags and/or internal labels were kept inside the new jars and, whenever possible, original external labels were reattached to the exterior. A new standardised, typewritten label was printed on ResistAll paper and placed inside each jar, presenting the basic data its content (Fig. 7E). After this, specimens were deposited in MUHNAC’s Wet Collections reserves, placed in compactor cabinets, and arranged by country of origin (sub-collection) and then taxonomically (Fig. 7F).

**Figure 8.**
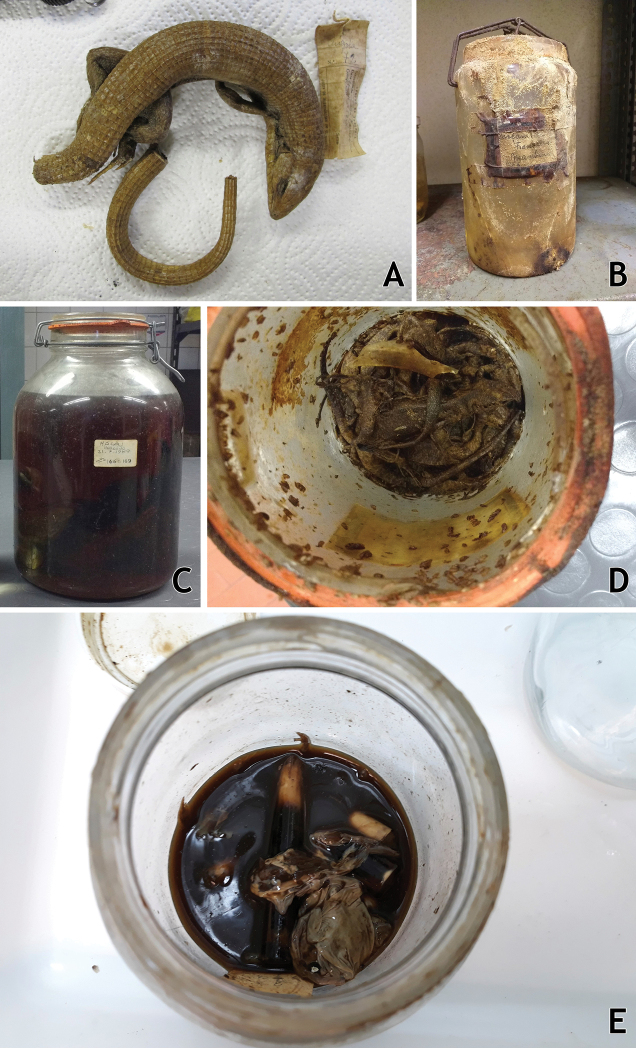
Examples of problems associated with the IICT herpetological collections **A** a dehydrated specimen of *Gerrhosaurus multilineatus* from Angola **B** a jar with extensive fungal growth on the inside and outside **C** lipid loss on a snake specimen from Macau **D** jar in which the preservative fluid has evaporated and the specimen was attacked by insect pests **E** rotten specimen of *Leptopelis* due to poor initial fixation and evaporation of the ethanol preservative. Photographs by Mariana P. Marques.

Some specimens presented critical conservation issues such as being dehydrated due to evaporation of the preservative fluid (Fig. 8A), fungal and bacterial growth (Fig. 8B), deposits of formaldehyde crystals, loss of proteins and lipids (Fig. 8C), or decomposition of the specimens (Fig. 8D, E). For dehydrated specimens we attempted a slow rehydration through the placement of the specimen in a humid atmosphere and staging in water overnight. Specimens with fungal and bacterial growth were washed in 70% ethanol and the growth carefully removed with cotton swabs. Specimens with deposits of formaldehyde crystals were washed with water then a series of steps of 20% increase in concentration of ethanol (20% for 30 minutes, 40% for 30 minutes, 60% for 30 minutes, 70% for final preservation); the few specimens that showed loss of proteins and lipids underwent similar treatment. Specimens that were soft due to problems during the fixation process were injected and immersed with formaldehyde for one to two days, depending on the size of the specimen. Specimens that were beyond salvation were discarded, but each was photographed for archival purposes and their associated data was collected whenever possible.

All specimens with locality data were georeferenced. Locality data are reported in the form of decimal degrees and use the WGS 84 map datum. Older (non-GPS) records were georeferenced using the GEOLocate web application (https://www.geo-locate.org). Whenever possible original maps, field books or collectors’ notes were consulted. Elevations are all reported as meters above sea level.

## Results

The collections are divided into eight sub-collections, respectively the Cabo Verde collection, the Guinea-Bissau collection, the São Tomé & Príncipe collection, the Angola collection, the Mozambique collection, the Portuguese India collection, the Macau collection, and the East Timor collection. Some specimens from Portugal and other African countries also exist in the collections, but represent a diminutive subset, mostly originating from occasional collecting events by IICT researchers or external donations. Combined, the eight sub-collections hold a total of 5173 specimens (3048 reptiles, 2125 amphibians). The largest sub-collection is that of Cabo Verde (1740 specimens), followed by Mozambique (1181 specimens), Guinea-Bissau (980 specimens), Angola (677 specimens), São Tomé & Príncipe (234 specimens), Macau (318 specimens), Portuguese India – Goa (26 specimens), and East Timor (17 specimens). The specimens were collected in 253 different localities across their respective countries. The collections hold 47 type specimens (two holotypes, 45 paratypes) of nine nominal taxa. Surprisingly, only 76 jars with an unknown number of specimens (due to the poor preservation or total destruction of the specimens) were discarded because they were beyond salvation. Detailed reviews of the different sub-collections are provided in the following accounts.

### Cabo Verde collection

The herpetofauna of the Cabo Verde archipelago has been reviewed in the past decades and is currently well known. The most recent account of its terrestrial reptiles was published by Vasconcelos et al. (2013), listing a total of 31 extant taxa, of which 22 are endemic to the archipelago. Seven exotic reptiles have been recorded in Cabo Verde (Vasconcelos et al. 2013; Ceríaco and Sousa 2017), and the only amphibian present in the archipelago is the exotic African Common Toad, *Sclerophrys regularis* (Reuss, 1833) (Vasconcelos et al. 2010b). These numbers are constantly being updated, as a new species has been recently described (Vasconcelos et al. 2020). The iconic giant skink, *Chioninia coctei* (Duméril & Bibron, 1839) is presumed to have gone extinct in the twentieth century due to a combination of human and ecological factors, and recent searches for this species have been unsuccessful (Ceríaco 2014).

This collection comprises a total of 1740 specimens (376 amphibians and 1364 reptiles), composed of one species of amphibians and 17 species and four subspecies of reptiles (Table 1). All the amphibians in the collection belong to the invasive African Common Toad (*Sclerophys regularis*), accounting for roughly 20% of the total collection. The reptile collection is composed of representatives from the families Gekkonidae, Phyllodactylidae and Scincidae, each represented by a single genus. Family Phyllodactylidae is the best represented with 871 specimens, followed by Scincidae with 489 and Gekkonidae with only four specimens. All specimens from the family Phyllodactylidae belong to endemic species of the genus *Tarentola*, and all species known to occur in Cabo Verde are represented in the collection, except *Tarentola boavistensis* Joger, 1993. Of the two recognised subspecies of *Tarentola gigas* (Bocage, 1875), only the nominal *T. g. gigas* is present in the collection, while both subspecies of *Tarentola protogigas* Joger, 1984 are represented (*T. p. protogigas* and *T. p. hartogi*). With 296 specimens, *Tarentola nicolauensis* Schleich, 1984 is the best represented species, followed by *Tarentola substituta* Joger, 1984, *T. gigas gigas* and Tarentola cf. caboverdiana Schleich, 1984 with 131, 128 and 124 specimens, respectively. The family Scincidae is represented by the endemic genus *Chioninia*, covering all known species except for the extinct *C. coctei*. At the subspecific level, *C. vaillantii vaillantii* (Boulenger, 1887) and *C. spinalis maioensis* (Mertens, 1955) are the only recognised subspecies that are represented in this collection. Within the Scincidae, *C. delalandii* (Duméril & Bibron, 1839) is the best represented species with 220 specimens, followed by *C. nicolauensis* (Schleich, 1987) with 90 specimens. The few specimens from the family Gekkonidae are assigned to *Hemidactylus* sp. and *Hemidactylus angulatus* Hallowell, 1854, which is the only exotic reptile from Cabo Verde that is represented in the collection.

**Table 1.** Overview of the Cabo Verde amphibian and reptile collections of IICT.

Family	Genus	Species	Localities – Accession number	References	Number of specimens
			(* denotes a type specimen)		
**AMPHIBIANS**					
**ANURA Duméril, 1806**					
**Bufonidae** (Gray, 1825)	*Sclerophrys* Tschudi, 1838	*Sclerophrys regularis* (Reuss, 1833)	**Brava Island**: Fajã D´Água – IICT/A 295–301/1969; Vinagre – IICT/A 273–294/1969;		376
			**Santiago Island**: Assomada – IICT/A 41–42/1969, 59–66/1969, 170–196/1969, 272/1969; Engenho – IICT/A 43–53/1969, 67–85/1969, 222–224/1969, 231–255/1969, 261–263/1969, 268–271/1969; Boa Entrada – IICT/A 54–58/1969, 86–91/1969, 264–267/1969, 1–11/1972; 18–31/1972; Boa Entrada stream – IICT/A 12–14/1969; Chão de Tanque – IICT/A 27–34/1969; Mato Sancho stream [or Mato Sanches stream] – IICT/A 1–11/1969; Picos – IICT/A 227–230/1969; Picos stream – IICT/A 225–226/1969; Praia Formosa – IICT/A 1–2/1993; Santa Catarina – IICT/A 10/1969, 16–26/1969, 256–260/1969, 32–37/1972, CV1–9, CV11, CV13; São Jorge dos Orgãos – IICT/A 103–169/1969; Sedeguma – IICT/A 35–40/1969, 92–102/1969, 197–214/1969, 15–17/1972; Santiago Island [unknown locality] – IICT/A 12–15/1969; 215–221/1969, CV10;		
			**São Nicolau Island**: Ribeira João – IICT/A 4–21/1970; Ribeira Brava – IICT/A 1–3/1970		
**TOTAL NUMBER OF AMPHIBIAN SPECIMENS**					**376**
**REPTILES**					
**SQUAMATA** Oppel, 1811					
**Gekkonidae** Gray, 1825	*Hemidactylus* Goldfuss, 1820	*Hemidactylus angulatus* Hallowell, 1854	**Santo Antão Island**: Ponta do Sol – IICT/R 292/1972; Porto Novo – CV18		3
			**Fogo Island**: São Filipe – IICT/R 442/1969		
		*Hemidactylus* sp.	**Santo Antão Island**: Ponta do Sol, Fantanha – IICT/R CV16		1
**Phyllodactylidae** Gamble, Bauer, Greenbaum & Jackman, 2008	*Tarentola* Gray, 1825	*Tarentola bocagei* Vasconcelos, Perera, Geniez, Harris & Carranza, 2012	**São Nicolau Island**: between Jucalinho and Carrissal – IICT/R 509–515/1970; Carvoeiro – IICT/R 354–357/1970; Preguiça– IICT/R 167–174/1970; Preguiça Airfield – IICT/R 152–159/1970, 161–166/1970, 175–180/1970, 599–603/1970, 624–631/1970; Ribeira das Queimadas – IICT/R 37/1970; Ribeira Maiamba – IICT/R 135–140/1970; Taboleiro – IICT/R 580–586/1970; São Nicolau Island [unknown locality] – IICT/R 48/1970, 58–59/1970, 499–509/1970		61
		Tarentola cf. caboverdiana Schleich, 1984	**Santo Antão Island**: Ponta do Sol – IICT/R 334–338/1972, CV15; Porto Novo – IICT/R 119–134/1972, 145–156/1972, 166–243/1972, 232–/1972; Santo Antão Island [unknown locality] – IICT/R 135–144/1972, 246–258/1969		124
		*Tarentola darwini* Joger, 1984	**Santiago Island**: Praia – IICT/R CV19; S. João Batista – IICT/R 448–449/1969		2
		*Tarentola fogoensis* Vasconcelos, Perera, Geniez, Harris & Carranza, 2012	**Fogo Island**: São Filipe – IICT/R 427–24871969, 435/1969, 439–441/1969, 444–446/1969; Fogo Island [unknown locality] – IICT/R 447/1969		10
		*Tarentola gigas gigas* (Bocage, 1875)	**Raso Islet**: Raso Islet [unknown locality] IICT/R 230–231/1970, 237/1970, 239–271/1970, 363/1970, 367–452/1969, 631–637/1970, CV3		128
		*Tarentola maioensis* Schleich, 1984	**Maio Island**: Airport – IICT/R 365–375/1969; Barreiro – IICT/R 377–381/1969; Calheta – IICT/R 349/1969, 358/1969; Lagoa – IICT/R 343–348/1969; Vila de Maio – IICT/R 334–338/1969; Maio Island [unknown locality] – IICT/R 339–342/1969, 359–364/1969; 376/1969, 382–389/1969; 391–398/1969		61
		*Tarentola nicolauensis* Schleich, 1984	**São Nicolau Island**: between Jucalinho and Carrissal – IICT/R 488–498/1970; between Ribeira Brava and Juncalinho– IICT/R 516–523/1970; Cabeçalinho – IICT/R 548–561/1970; Calejão – IICT/R 212–229/1970, 289–296/1970, 638–650/1970;		296
**Phyllodactylidae** Gamble, Bauer, Greenbaum & Jackman, 2008	*Tarentola* Gray, 1825	*Tarentola nicolauensis* Schleich, 1984	Carvoeiro – IICT/R 340–353/1970, 358–362/1970, 598–623/1970; Chanzinha – IICT/R 284–288/1970; Preguiça – IICT/R 141–151/1970; Perguiça port – IICT/R 297–315/1970; Ribeira da Prainha – IICT/R 187–211/1970; Ribeira de Caixa – IICT/R 316–319/1970; Ribeira Maiamba – IICT/R 126–134/1970, 181–186/1970; Ribeira Seca – IICT/R 320–334/1970; S. João – IICT/R 272–277/1970; S. Nicolau hostel – IICT/R 475/1970; Taboleiro – IICT/R 533–539/1970, 562–579/1970, 583A–585A/1970, 587–595/1970; Ribeira Brava – IICT/R 115–125/1970, 335–339/1970 ; Ribeira Brava surroundings – IICT/R 476–487/1970; São Nicolau Island [unknown locality] – IICT/R 596–597/1970		
		*Tarentola protogigas hartogi* Joger, 1993	**Brava Island**: Achada do Favatal – IICT/R 403–405/1969, 412–421/1969; Vinagre – IICT/R 422–425/1969		24
			**Rombos Islet**: 406–411/1969, CV32		
		*Tarentola protogigas protogigas* Joger, 1984	**Fogo Island**: São Filipe – IICT/R 426/1969, 429/1969, 433–434/1969, 436–43871969; São Filipe vivarium – IICT/R 443/1969; Vale de Cavaleiros lighthouse – IICT/R 292/1969		9
		*Tarentola raziana* Schleich, 1984	**Branco Islet**: Branco Islet [unknown locality] – IICT/R 470–474/1969		14
			**Raso Islet**: Raso Islet [unknown locality] IICT/R 232–237/1970, 364–366/1970		
		*Tarentola rudis* Boulenger, 1906	**Santiago Island**: Praia – IICT/R 350–357/1969, 399/1969		10
		*Tarentola substituta* Joger, 1984	**São Vicente Island**: Baia das Gatas – IICT/R 301–31871972; Calhau – IICT/R 321–333/1972; Mato Inglês – IICT/R 339–377/1972; Monte Sossego – IICT/R 396–412/1972; Ribeira Passarão – IICT/R 381–395/1972; São Pedro – IICT/R 259–287/1970;		131
		*Tarentola* sp.	**Fogo Island**: Fogo Island [unknown locality] – IICT/R 431/1969		1
**Scincidae** Cuvier, 1808	*Chioninia* Gray, 1845	*Chioninia delalandii* (Duméril & Bibron, 1839)	**Brava Island**: Achada do Favatal – IICT/R 237–255/1969; Nova Sintra – IICT/R 213–216/1969, 221–236/1969, 271–276/1969, CV14; Nova Sintra graveyard – IICT/R 217–220/1969, 221A–223A/1969; Senhora do Monte – IICT/R 256–261/1969; Vinagre – IICT/R 264–270/1969; Brava Island [unknown locality] – IICT/R 193/1969, 207–212/1969	Pinheiro (1986, 1990)	220
			**Maio Island**: Calheta – IICT/R 26–30/1969, 38–43/1969, 47/1969, 49/1969; Maio Island [unknown locality] – IICT/R 90/1969, 96–97/1969		
			**Santiago Island**: Assomada – IICT/R 158 Boa Entrada – IICT/R 159/1969, 161/1969, 171/1969; Boa Entrada – IICT/R 131–133/1969, 144–147/1969, 156–159/1969, 189–192/1969, 45–47/1972; Chão da Fazenda – IICT/R 24–26/1972; Praia – IICT/R 58–74/1969; Santa Catarina – IICT/R 27–32/1972, 53–72/1972, 79–93/1972; Tarrafal – IICT/R 176/1969; Santiago Island [unknown locality] – IICT/R 162–165/1969, 288–291/1969, 33–39/1972, CV29–30		
			**Fogo Island**: São Filipe – IICT/R 297–300/1969, 303/1969, 323–327/1969; São Filipe vivarium – IICT/R 328–331/1969; Fogo Island [unknown locality] – IICT/R 292–332/1969, CV2		
		*Chioninia fogoensis* (O’Shaughnessy, 1874)	**Santo Antão Island**: Chão do Mocho – IICT/R 13–19/1972; Porto Novo – IICT/R 10–11/1972, 234–245/1972, 1001–1012/1972, CV17; Vale Paúl stream – IICT/R 4–9/1972, 298–300/1972; Santo Antão Island [unknown locality] – IICT/R 1–3/1972, 12/1972, 158–165/1972, 379–380/1972	Pinheiro (1990)	56
		*Chioninia nicolauensis* (Schleich, 1987)	**São Nicolau Island**: Caleijão – IICT/R 79–85/1970; Preguiça Airfield – IICT/R 12–13/1970, 160/1970; Ribeira das Queimadas – IICT/R 41–47/1970, 49–57/1970; Ribeira João – IICT/R 1–11/1970; São Nicolau Island [unknown locality] – IICT/R 14–40/1970, 60–78/1970, 102–106/1970, 651/1970	Pinheiro (1990)	90
**Scincidae** Cuvier, 1808	*Chioninia* Gray, 1845	*Chioninia spinalis maioensis* (Mertens, 1955)	**Maio Island**: Alcatraz – IICT/R 98–108/1969; Bumba stream – IICT/R 9–14/1969; Calheta – IICT/R 26A–37A/1969; 44–46/1969, 48/1969, 50/1969, 52–56/1969; Maio Island [unknown locality] – IICT/R 1–8/1969, 15–19/1969, 109–110/1969; Lage-Branca Islet – IICT/R 75–83/1969, CV3	Pinheiro (1990)	64
		*Chioninia strangeri* (Gray, 1845)	**Raso Islet**: Raso Islet [unknown locality] IICT/R 86–114/1970	Pinheiro (1990)	19
		*Chioninia vaillantii vaillantii* (Boulenger, 1887)	**Santiago Island**: Assomada – IICT/R 166–170/1969, 180–188/1969; Boa Entrada – IICT/R 118–120/1967, 125–130/1967, 148–150/1969, 172–175/1969, 1340–1343/1969; Tarrafal – IICT/R 177–178/1969; Santiago Island [unknown locality] – IICT/R 121/1969, 138–142/1969, 151–153/1969, 44/1972, CV28	Pinheiro (1986)	40
**TOTAL NUMBER OF REPTILES SPECIMENS**					**1364**

**Figure 9.**
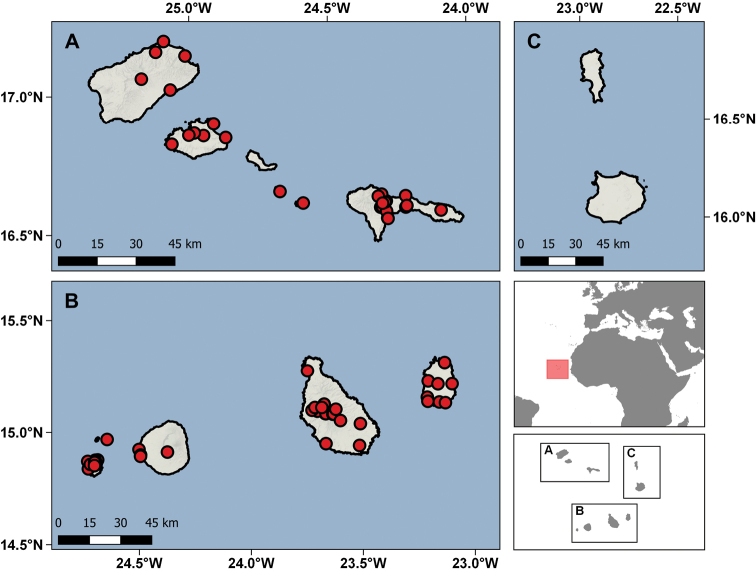
Distribution of the Cabo Verde localities represented in the IICT herpetological collections.

The geographic range of this collection covers 74 different localities from ten islands and islets: São Nicolau, Santiago, Brava, Santo Antão, Maio, Raso, Fogo, São Vicente, Rombos, and Branco (Table 2; Fig. 9). Of all the major islands of the archipelago, only three are not represented: Santa Luzia in the Desertas group, and the eastern islands of Sal and Boavista. São Nicolau is the best represented island, with 474 specimens from 24 different localities, while the Rombos islets are the least sampled with only seven specimens. Collecting events took place between 1967 and 1994, although most of the material was collected between 1969 and 1972 in expeditions organised by the CZL, especially aimed at collecting birds and invertebrates.

**Table 2.** Gazetteer of Cabo Verde localities of IICT specimens. Latitude and longitude decimal coordinates are presented in WGS-84 projection.

Island/Islet	Verbatim locality	Current locality	Latitude and Longitude	Uncertainty (meters)	Elevation (meters)	Number of taxa/records
Brava	Achada do Favatal [or Ach do Favatal]	Achada do Favatal (currently a waste dump)	14.877435, -24.686155	1616	308	2/18
Brava	Achada do Ferreiro	Achada do Ferreiro	14.837713, -24.727839	1376	238	1/11
Brava	Braga	Brava Island [or Brava]	14.852066, -24.700687	5640	905	1/6
Brava	Cemitério de Nova Sintra	Nova Sintra graveyard	14.876142, -24.69713	125	478	1/7
Brava	Fajã D´Água	Fajã D´Água	14.870898, -24.730602	500	39	1/7
Brava	Nova Sintra	Nova Sintra	14.870483, -24.695545	747	497	2/30
Brava	Senhora do Monte	Senhora do Monte	14.857583, -24.719033	500	640	1/6
Brava	Vinagre	Vinagre	14.866667, -24.7	350	634	3/33
Brava	Ilha Brava	Brava Island	14.852066, -24.700687	5640	905	1/1
Maio	Aeroporto	Airport	15.156885, -23.214155	1659	11	1/11
Maio	Alcatraz	Alcatraz	15.217212, -23.103711	206	24	1/11
Maio	Barreiro	Barreiro	15.135554, -23.160517	370	31	1/5
Maio	Calheta	Calheta	15.229421, -23.210159	800	9	3/37
Maio	Ilhéu Lage-Branca	Lage-Branca Islet	15.313, -23.137	47	–	1/10
Maio	Lagoa	Lagoa	15.132533, -23.133345	540	3	1/6
Maio	Vila de Maio	Vila de Maio	15.138703, -23.211588	730	22	1/5
Maio	Ribeira de Bumba	Bumba stream [undetermined locality]	–	–	–	1/6
Maio	Ilha de Maio	Maio Island	15.216667, -23.166667	13613	82	2/61
Santiago	Assomada	Assomada, Santa Catarina	15.098508, -23.67269	3036	542	1/40
Santiago	Boa Entrada	Boa Entrada, Santa Catarina	15.125985, -23.674844	420	475	4/77
Santiago	Ribeira Boa Entrada	Boa Entrada stream	15.125498, -23.675343	1017	475	1/3
Santiago	Chã da Fazenda Sta. Catarina	Chão da Fazenda, Santa Catarina	15.083664, -23.669757	5513	371	1/2
Santiago	Santa Catarina, Chão de Tanque	Chão de Tanque, Santa Catarina	15.094417, -23.705235	506	237	1/8
Santiago	Santa Catarina – Engenho [or Engenho]	Engenho, Santa Catarina	15.083333, -23.666667	3036	382	3/84
Santiago	Ribeira de Mato Sancho [or Ribeira de Mato Sanches]	Mato Sanches stream [or Mato Sancho stream]	15.098698, -23.728967	1677	349	1/11
Santiago	Picos	Picos	15.081013, -23.636095	559	444	1/4
Santiago	Ribeira dos Picos	Picos stream	15.081738, -23.632877	1257	-	1/2
Santiago	Praia	Praia	14.942039, -23.516667	4271	69	2/27
Santiago	Praia Formosa	Praia Formosa	15.039177, -23.51306	1318	110	1/2
Santiago	S. João Batista	S. João Batista	14.95, -23.666667	3036	135	1/2
Santiago	Santa Catarina [or Santa Catarina - Ilha do Sanque]	Santa Catarina	15.110938, -23.716136	7374	293	2/81
Santiago	Sedeguma	Sedeguma, Santa Catarina	15.111, -23.685382	768	510	1/37
Santiago	São Jorge dos Orgãos	São Jorge dos Orgãos	15.052822, -23.601954	802	313	1/67
Santiago	Tarrafal	Tarrafal	15.275278, -23.749454	1634	21	2/3
Santiago	Ilha de Santiago	Santiago Island	15.103222, -23.622722	29199	242	3/40
Santo Antão	Chão do Mocho	Chão do Mocho	17.161455, -25.119461	2046	187	1/7
Santo Antão	Ponta do Sol [or Ponta do Sol, Fantanha]	Ponta do Sol [or Ponta do Sol, Fantanha]	17.200315, -25.091084	635	30	3/8
Santo Antão	Porto Novo	Porto Novo	17.025443, -25.066603	1493	47	3/120
Santo Antão	Vale do Paúl [or Vale Paúl, Ribeira]	Vale do Paúl [or Vale Paúl, stream]	17.148052, -25.013314	1217	7	1/12
Santo Antão	Ilha de Santo Antão	Santo Antão Island	17.064672, -25.170897	23523	1077	2/36
São Nicolau	Arredores Vila Ribeira Brava	Ribeira Brava surroundings	16.624659, -24.286525	1125	36	2/12
São Nicolau	Cabeçalinho	Cabeçalinho	16.602177, -24.306767	430	300	1/14
São Nicolau	Calejão	Calejão	16.604442, -24.301217	303	221	2/46
São Nicolau	Carvoeiro	Carvoeiro	16.648998, -24.303291	428	99	2/50
São Nicolau	Chanzinha	Chanzinha	16.616616, -24.29656	507	107	1/5
São Nicolau	entre Jucalinho e Carrissal	between Jucalinho and Carrissal	16.591956, -24.088655	6465	467	2/18
São Nicolau	entre Ribeira Brava e Juncalinho	between Ribeira Brava and Juncalinho	16.643644, -24.216589	12531	54	1/8
São Nicolau	Perguiça	Perguiça	16.563247, -24.281361	301	61	2/19
São Nicolau	Campo de Aviação da Preguiça	Preguiça Airfield	16.588013, -24.285383	1296	181	3/36
São Nicolau	Porto da Preguiça	Preguiça port	16.561723, -24.280177	159	6	1/19
São Nicolau	Ribeira da Prainha	Ribeira da Prainha	16.620503, -24.290903	1719	96	1/24
São Nicolau	Ribeira das Queimadas	Ribeira das Queimadas	16.641588, -24.315379	300	241	2/18
São Nicolau	Ribeira de Caixa	Ribeira de Caixa [undetermined locality]	–	–	–	1/4
São Nicolau	Ribeira João	Ribeira João [undetermined locality]	–	–	–	2/29
São Nicolau	Ribeira Maiamba	Ribeira Maiamba [undetermined locality]	–	–	–	3/21
São Nicolau	Ribeira Seca	Ribeira Seca	16.608037, -24.212366	24792	175	1/15
São Nicolau	S. João	S. João	16.602678, -24.215949	22806	200	1/6
São Nicolau	Pousada S. Nicolau	S. Nicolau hostel [undetermined locality]	–	–	–	1/1
São Nicolau	Taboleiro	Taboleiro [undetermined locality]	–	–	–	3/44
São Nicolau	Vila da Ribeira Brava [or Vale Paúl, Ribeira]	Ribeira Brava [or Vale Paúl, Ribeira]	16.616667, -24.3	301	142	2/19
São Nicolau	Ilha de São Nicolau	São Nicolau Island	16.608037, -24.212366	24792	175	3/66
São Vicente	Baia das Gatas	Baia das Gatas	16.903244, -24.909886	301	6	1/18
São Vicente	Calhau	Calhau	16.853122, -24.86641	1482	8	1/13
São Vicente	Mato Inglês	Mato Inglês	16.860123, -24.946237	649	191	1/39
São Vicente	São Pedro	São Pedro	16.828744, -25.060674	1821	6	1/29
São Vicente	Ribeira Passarão	Ribeira Passarão	16.869242, -24.979704	1425	27	1/15
São Vicente	Monte Sossego	Monte Sossego	16.86152, -24.999323	414	30	1/17
Fogo	Posto de Fogo, Cavaleiros	Vale de Cavaleiros lighthouse	14.923969, -24.501743	300	44	1/1
Fogo	S. Filipe	São Filipe	14.899416, -24.494312	1484	132	4/27
Fogo	Viveiros de S. Filipe	S. Filipe vivarium	14.893628, -24.494822	1559	92	2/5
Fogo	Ilha do Fogo	Fogo Island	14.912456, -24.374662	16064	2004	3/6
Branco	Ilhéu Branco	Branco Islet	16.658552, -24.670287	2405	276	1/5
Raso	Ilhéu Raso	Raso Islet	16.617111, -24.586456	2405	48	3/156
Rombos	Ilhéus Rombos	Rombos Islets	14.96853, -24.644065	2484	9	1/7

Part of the material assigned to the genus *Chioninia* was studied and published by Margarida Pinheiro on two different occasions (Pinheiro 1986, 1990), while a few geckos of the genera *Hemidactylus* and *Tarentola* were used by José Jesus for sequencing and phylogenetic analysis (Jesus et al. 2001, 2002). More recently the collection has been consulted by Raquel Vasconcelos (1980–to date) for several ecological and dietary studies of species of the genus *Tarentola*. No type material is present in the collection.

This is the largest known herpetological collection from Cabo Verde, with 1740 specimens, and is followed by relevant collections from the MNHN with a total of 137 specimens, the NHMUK with 104 specimens, the MCNB with 102 specimens, and the TCWC with 97 specimens (data retrieved from GBIF.org in March 2020). Other collections worldwide generally do not exceed 50 specimens (data retrieved from GBIF.org in February 2021).

The dataset of this collection is available at GBIF (Ceríaco et al. 2018b; https://www.gbif.org/dataset/e5dd4343-8bd9-449a-bef5-47c655968225).

### Guinea-Bissau collection

The herpetofauna of Guinea-Bissau is one of the most poorly known in the region. The most recent checklist was published by Auliya et al. (2012), listing respectively 25 and 73 species of amphibians and reptiles. Historically the country has been poorly surveyed in terms of its biodiversity, with the first available data on the country’s herpetofauna provided by Bocage (1866, 1867, 1872, 1873, 1896a, b), Ferreira (1902), and Boulenger (1905, 1906). The Swiss naturalist Albert Monard provided the first country-wide revision of the herpetofauna (Monard 1940a, b), which included the description of new species (*Agama boensis*, *Latastia ornata*) and the first records of previously unrecorded species for the country. This collection is still extant in the MHNC and is composed of a total of 329 (191 amphibians and 138 reptiles) specimens. From 1944 to 1946, Fernando Frade led an extensive zoological expedition to the country (Frade et al. 1946), which resulted in the collection of approximately a thousand specimens that were later studied by Sara Manaças and constitute the bulk of the IICT collections (see below). Besides the Auliya et al. (2012) report on the herpetofauna of Bijagós, and two small contributions on amphibians (Rebelo 2018a) and reptiles (Rebelo 2018b) from “João Vieira e Poilão” National Park and a master’s thesis on the amphibians and reptiles of Boé area (Cabuy 2014), no modern data exists on its terrestrial herpetofauna.

The IICT collection comprises 980 specimens (604 amphibians and 376 reptiles), covering 12 species of amphibians and 37 species of reptiles (Table 3), corresponding to roughly half of the known taxa for the country. The amphibian collection comprises representatives of seven families and eight genera. These specimens are dominated by representatives of common species of the following families: Dicroglossidae, with 252 representatives of Crowned Bullfrogs, *Hoplobatrachus occipitalis* (Günther, 1858); Bufonidae, with 188 representatives of African Common Toads, *Sclerophrys regularis*; and Ptychadenidae, with 99 representatives of Mascarene Grass Frogs, *Ptychadena mascareniensis* (Duméril & Bibron, 1841), 27 representatives of Bibron’s Grass Frog, *Ptychadena bibroni* (Hallowell, 1845), and a single representative of Ansorge’s Grass Frog, Ptychadena cf. ansorgii (Boulenger, 1905). The first four taxa make up about 94% of the amphibian specimens, and approximately 60% of the entire collection. The reptile collection, despite being smaller, is taxonomically more diverse. It comprises representatives of 15 families and 26 genera. Within Squamates, the families Scincidae, Agamidae, and Lamprophiidae are the best represented, with 82, 82, and 52 specimens respectively. Families Lamprophiidae and Colubridae are those which have a greater diversity of species (seven species each), while the remaining families have no more than three species represented in the collection.

**Table 3.** Overview of the Guinea Bissau amphibian and reptile collections of IICT.

Family	Genus	Species	Localities – Accession number	References	Number of specimens
			(* denotes a type specimen)		
**AMPHIBIANS**					
**ANURA Duméril, 1806**					
**Pipidae** Gray, 1825	*Pseudhymenochirus* Chabanaud, 1920	*Pseudhymenochirus merlini* Chabanaud, 1920	Pitche – IICT/A 221-1946	Manaças (1947)	1
	*Xenopus* Wagler, 1827	*Xenopus tropicalis* (Gray, 1864)	Bissau – IICT/A 106 to 115–1945; Calequisse, Chachungo – IICT/A 143-1945	Manaças (1947)	11
**Bufonidae** (Gray, 1825)	*Sclerophrys* Tschudi, 1838	*Sclerophrys regularis* (Reuss, 1833)	Bissalanca – IICT/A 15–20/1945; Bissau – IICT/A GB1; Bissorã – IICT/A 171–274/1945; Cacine – IICT/A 72–89/1946, 91–93/1946; Mansoa [or Mansôa] – IICT/A 17/1946; Marques Mano – IICT/A 1–10/1945, 166/1945; Pecixe – IICT/A 145–149/1945, 151–159/1945; Pitche – IICT/A 164–257/1946; Tor – IICT/A 28–31/1945;	Manaças (1949)	179
		*Sclerophrys* sp.	Bissorã – IICT/A 258–266/1945		9
**Hyperoliidae** Laurent, 1943	*Hyperolius* Rapp, 1842	*Hyperolius concolor* (Hallowell, 1844)	Bissalanca – IICT/A 167/1945; Mansoa [or Mansôa] – IICT/A 25-1946; Marques Mano Farm – IICT/A 160–163/1945, 165/1945, 168–170/1945; Pitche – IICT/A 178-1946	Manaças (1949)	11
		*Hyperolius* sp.	between Buba and Corubal river – IICT/A-1990; Caiomete – IICT/A 131–132/1945; Catió – IICT/A GB2; Marques Mano Farm – IICT/A 1–4/1947, 164/1945	Manaças (1949)	9
**Ptychadenidae** Dubois, 1987	*Ptychadena* Boulenger, 1917	*Ptychadena ansorgii* (Boulenger, 1905)	Cacine – IICT/A 342-1946		1
		*Ptychadena bibroni* (Hallowell, 1845)	Bissorã – IICT/A 50–52/1946; Cutiá, Mansoa [or Mansôa] – IICT/A 26–49/1946	Manaças (1949)	27
		*Ptychadena mascareniensis* (Duméril & Bibron, 1841)	Bissorã – IICT/A 194–195/1945, 284–333/1946, 289–295/1945; Cacine – IICT/A 66–71/1946, 75/1946; Catió – IICT/A 66–71/1946, 102–103/1946, 106–112/1946, 141/1946; Catió, Cufar – IICT/A 151/1946; Cutiá, Mansoa [or Mansôa] – IICT/A 283/1946; Gabu] – IICT/A 97/1945; Madina do Boé – IICT/A 230–243/1945; Mansoa [or Mansôa] – IICT/AIICT/A 8–1946; Prabis – IICT/A 50–54/1945	Manaças (1949)	99
**Dicroglossidae** Anderson, 1871	*Hoplobatrachus* Peters, 1863	*Hoplobatrachus occipitalis* (Günther, 1858)	Bafatá – IICT/A 100–105/1945; Bissalanca – IICT/A 12–14/1945; Bissau – IICT/A 116–119/1945; Bissorã – IICT/A 191–193/1945, 196–207/1945, 246–250/1945, 254–257/1945, 268–273/1945, 275–288/1945; Calequisse, Chachungo – IICT/A 154–156/1945; Cachungo – IICT/A 120–130/1945, 133–142/1945; Catió – IICT/A 94–98/1946, 104/1946, 116/1946, 120–140/1946, 142–147/1946; 156–169/1946; Catió, Cufar – IICT/A 148–150/1946, 152–155/1946; Farim – IICT/A 68–96/1945; Gabu – IICT/A 98–99/1945; Mansoa [or Mansôa] – IICT/A 1–7/1946, 9–24/1946, 53–65/1946, 258–282/1946, 336–341/1946; Parabis – IICT/A 36–49/1945; Pecixe – IICT/A 150/1945; Marques Mano Farm – IICT/A 11/1945; São Domingos – IICT/A 55–57/1945; Tor – IICT/A 21–27/1945, 32–35/1945	Manaças (1949)	252
**Ranidae** Batsch, 1796	*Amnirana* Dubois, 199	*Amnirana galamensis* (Duméril & Bibron, 1841)	Catió – IICT/A 99–101/1946	Manaças (1949)	3
**GYMNOPHIONA** Müller, 1832					
**Dermophiidae** Taylor, 1969	*Geotrypetes* Peters, 1880	*Geotrypetes seraphini* (Duméril, 1859)	Machado Farm – IICT/A 296–297/1945		2
**TOTAL NUMBER OF AMPHIBIAN SPECIMENS**					**604**
**REPTILES**					
**CROCODYLIA** Gmelin, 1789					
**Crocodylidae** Cuvier, 1808	*Crocodylus* Laurenti, 1768	*Crocodylus suchus* Geoffroy, 1807	Bijimita – IICT/R 102–109/1954		8
**SQUAMATA** Oppel, 1811					
**Gekkonidae** Gray, 1825	*Hemidactylus* Goldfuss, 1820	*Hemidactylus angulatus* Hallowell, 1854	Bissalanca – IICT/R 97/1945, 145/1945, 184/1945; Bissau – IICT/R 152/1945, 128–130/1946; Buba – IICT/R GB4; Cacine – IICT/R 17/1946, 19/1946/, 28/1946; Chitole [or Xitole] – IICT/R 12/1946; Mansoa [= Mansôa] – IICT/R 1/1946; Marques Mano Farm – IICT/R 2–3/1944, 44/1945, 60–61/1945, 257–259/1945, 262/1945, 264/1945, 267/1945, 269/1945, 278/1945; Gabu – IICT/R A–B/1962; São Vicente – IICT/R 1A-1946	Manaças (1951)	29
**Phyllodactylidae** Gamble, Bauer, Greenbaum & Jackman	*Tarentola* Gray, 1825	*Tarentola ephippiata senegambiae* Joger, 1984	Bafatá – IICT/R 291/1945; Pitche – IICT/R 122/1946	Manaças (1951)	2
**Amphisbaenidae** Gray, 1825	*Cynisca* Duméril & Bibron, 1839	*Cynisca feae* (Boulenger, 1906)	Bissalanca – IICT/R 64/1945; Bissau – IICT/R 53/1945; Marques Mano Farm – IICT/R 28–30/1945, 40/1945, 42–43/1945, 46–48/1945, 54/1945, 65–71/1945, 76–77/1945, 76A/1945, 92–96/1945, 138–139/1945	Manaças (1955)	29
**Varanidae** Hardwicke & Gray, 1824	*Varanus* Merrem, 1820	*Varanus exanthematicus* (Bosc, 1792)	Bissalanca – IICT/R 45/1945, 51/1945, 80/1945; Brene – IICT/R 52/1945; Cachungo – IICT/R 192/1945, 201/1945; Pecixe – IICT/R 219–220/1945; Pitche – IICT/R 112/1945; Tor – IICT/R 118/1945	Manaças (1955)	10
		*Varanus niloticus* (Linnaeus, 1758)	Ilha Formosa – IICT/R 155/1945		1
**Scincidae** Cuvier, 1808	*Mochlus* Günther, 1864	*Mochlus guineensis* Peters, 1879	Cacine – IICT/R 29/1946	Manaças (1951)	1
	*Trachylepis* Fitzinger, 1843	*Trachylepis affinis* (Gray, 1838)	Bissalanca – IICT/R 183/1945; Bissorã – IICT/R 305–306/1945; Cacine – IICT/R 15–16/1946, 18/1946, 25–27/1946, 30/1946; Calequisse, Chachungo – IICT/R 202/1945; Madina do Boé – IICT/R 77–94/1946; Marques Mano Farm – IICT/R 4–5/1945, 9/1945, 137/1945, CZ000011298–11300; Pecixe – IICT/R 215–216/1945; Pitche – IICT/R 50/1946, 95–111/1946	Manaças (1951)	56
		*Trachylepis perroteti* (Duméril & Bibron, 1839)	Bissorã – IICT/R 309/1945; Cacine – IICT/R 31/1946; Calequisse, Chachungo – IICT/R 193–199/1945, 203/1945; Marques Mano Farm – IICT/R 32–33/1945, 254–255/1945, 254A–255A/1945, 256/1945, 260/1945, 261/1945, 261A/1945, 265/1945, 265A/1945, 266/1945, Guiné-Bissau [undetermined locality] – IICT/R 46/1964, 46ª/1964	Manaças (1951)	25
**Agamidae** Gray, 1827	*Agama* Daudin, 1802	Agama cf. boensis Monard, 1940	Pitche – IICT/R 44/1946, 76/1946; Guiné-Bissau [undetermined locality] – IICT/R GB6	Manaças (1951)	3
		*Agama picticauda* Peters, 1877	Bafatá – IICT/R 290/1945; Bambadinca – IICT/R 175/1945; Bissau, city center – IICT/R 228/1945, Bissorã – IICT/R 298/1945, 310–311/1945; Cachungo – IICT/R 205–209/1945, 209A/1945, 211–214/1945; Chitole [or Xitole] – IICT/R 8–11/1946; Cutiá, Mansoa [or Mansôa] – IICT/R 4/1946; Gabu – IICT/R 159–166/1945; Ilha Formosa – IICT/R 157–158/1945; Mansoa [or Mansôa] – IICT/R 5/1946; Marques Mano Farm – IICT/R 11–13/1945, 34–36/1945, 98/1945; Pecixe – IICT/R 185–187/1945, 189/1945, 218/1945, 221–223/1945; Pitche – IICT/R 47–49/1946, 51–65/1946, 67–75/1946, 121/1946; Tor IICT/R 119–121/1945, 140–141/1945	Manaças (1951)	79
		*Agama* sp.	Guiné-Bissau [undetermined locality] – IICT/R GB7		1
**Chamaeleonidae** Gray, 1825	*Chamaeleo* Laurenti, 1768	*Chamaeleo gracilis* Hallowell, 1842	Bissorã – IICT/R 209/1945, 301–302/1945, 304/1945, 308/1945; Brene – IICT/R 62/1946; Cacine – IICT/R 14/1946, 20/1946, 32/1946; Catió – IICT/R 39–40/1946; Chitole [or Xitole] – IICT/R 13/1946; Mansoa [or Mansôa] – IICT/R 2/1946; Nhampurbani, Pitche – IICT/R 123/1946; Marques Mano Farm – IICT/R 31/1945, 72/1945; Parabis – IICT/R 146–151/1945; Tor – IICT/R 123–129/1945, 132–133/1945	Manaças (1951)	31
**Chamaeleonidae** Gray, 1825	*Chamaeleo* Laurenti, 1768	*Chamaeleo senegalensis* Daudin, 1802	Bijimita – IICT/R 130/1945; Bissorã – IICT/R 294–295/1945, 297/1945, 299–300/1945; 307/1945; Mansoa [or Mansôa] – IICT/R 3/1946		8
SERPENTES					
**Typhlopidae** Merrem, 1820	*Afrotyphlops* Broadley & Wallach, 2009	*Afrotyphlops punctatus* (Leach, 1819)	Marques Mano Farm – IICT/R 1/1939, 5/1947; Guiné-Bissau [undetermined locality] – IICT/R 282/1945	Manaças (1955)	3
**Leptotyphlopidae** Stejneger, 1892	*Myriopholi* Hedges, Adalsteinsson & Branch, 2009	*Myriopholis narirostris* (Peters, 1867)	Marques Mano Farm – IICT/R 49/1945	Manaças (1955)	1
**Pythonidae** Fitzinger, 1826	*Python* Daudin, 1803	*Python sebae* (Gmelin, 1789)	Mandinga – IICT/R GB10		1
**Viperidae** Oppel, 1811	*Bitis* Gray, 1842	*Bitis arietans* (Merrem, 1820)	Marques Mano Farm – IICT/R 283/1945		1
	*Causus* Wagler, 1830	*Causus maculatus* (Hallowell, 1842)	Cachungo – IICT/R 240/1945		1
**Lamprophiidae** Fitzinger, 1843	*Atractaspis* Smith, 1849	*Atractaspis aterrima* Günther, 1863	Buba-Tombó – IICT/R GB11; Cacine – IICT/R 35/1946; Machado Farm – IICT/R 11/1947; Guiné-Bissau [undetermined locality] – IICT/R GB12		4
	*Boaedon* Duméril, Bibron & Duméril, 1854	*Boaedon fuliginosus* (Boie, 1827)	Bijimita – IICT/R 102/1945; Bissalanca – IICT/R 79/1945; Bissau – IICT/R 177/1945; Brene – IICT/R 74/1945; Marques Mano Farm – IICT/R 1/1944, 26/1945, 50/1945, 74/1945; Machado Farm – IICT/R 12/1987	Manaças (1955)	8
		*Boaedon* sp.	Marques Mano Farm – IICT/R 1/1944; Tor – IICT/R 113/1945	Manaças (1955)	2
	*Limaformosa* Broadley, Tolley, Conradie, Wishart, Trape, Burger, Kusamba, Zassi-Boulou & Greenbaum, 2018	*Limaformosa crossi* (Boulenger, 1895)	Tor – IICT/R 112/1945, 243/1945		2
	*Lycophidion* Fitzinger, 1843	*Lycophidion albomaculatum* Steindachner, 1870	Bissalanca – IICT/R 21/1945, 241/1945, Bissau – IICT/R 229/1945; Marques Mano Farm – IICT/R 27/1945; Guiné-Bissau [undetermined locality] – IICT/R GB14	Manaças (1955)	5
		*Lycophidion irroratum* (Leach, 1819)	Marques Mano Farm – IICT/R 135/1945; Machado Farm – IICT/R 275–276/1945, 280/1945	Manaças (1955)	4
	*Psammophis* Boie, 1825	*Psammophis elegans* (Shaw, 1802)	Bissalanca – IICT/R 22–23/1945, 58/1945; Catió – IICT/R 38/1946; Marques Mano Farm – IICT/R 14 15/1945; Pecixe – IICT/R 239/1945; Tor – IICT/R 88/1945, 111/1945, 117/1945	Manaças (1955)	10
		*Psammophis lineatus* (Duméril, Bibron & Duméril, 1854)	Bafatá – IICT/R 2/1953; Catió – IICT/R 3871946; Machado Farm – IICT/R 263/1945	Manaças (1955)	3
		*Psammophis afroccidentalis* Trape, Böhme & Mediannikov, 2019	Bijmita – IICT/R 101/1945; Bissau – IICT/R 126/1946; Cacine – IICT/R 21–22/1946, 34/1946; Catió – IICT/R 37/1946; Mandinga – IICT/R B9; Marques Mano Farm – IICT/R 25/1945; Tor – IICT/R 122/1945, 134/1945, 134A/1945, 142–143/1945	Manaças (1955)	13
		*Psammophis* sp.	Guiné-Bissau [undetermined locality] – IICT/R GB1		1
**Elapidae** Boie, 1827	*Naja* Laurenti, 1768	*Naja haje* (Linnaeus, 1758)	Guiné-Bissau [undetermined locality] – IICT/R 227		1
**Colubridae** Oppel, 1811	*Crotaphopeltis* Fitzinger, 1843	*Crotaphoepltis hotamboeia* (Laurenti, 1768)	Bafatá – IICT/R 287/1945; Bijimita – IICT/R78/1945; Bissalanca – IICT/R 103/1945, 24871945, 124/1946; Bissau, city center – IICT/R 10/1945, 182/1945; Machado Farm – IICT/R 277/1945; Guiné-Bissau [undetermined locality] – IICT/R GB13	Manaças (1955)	9
	*Dasypeltis* Wagler, 1830	*Dasypeltis confusa* Trape & Mané, 2008	Bissalanca – IICT/R 231/1945	Manaças (1955)	1
**Colubridae** Oppel, 1811	*Dasypeltis* Wagler, 1830	*Dasypeltis gansi* Trape & Mané, 2006	Cachamba – IICT/R GB5		1
	*Dispholidus* Duvernoy, 1832	*Dispholidus typus* (Smith, 1828)	Cacine – IICT/R 23/1946	Manaças (1955)	1
	*Grayia* Günther, 1858	*Grayia smithi* (Leach, 1818)	Machado Farm – IICT/R 15/1947	Manaças (1955)	1
	*Philothamnus* Smith, 1840	*Philothamnus irregularis* (Leach, 1819)	Bijimita – IICT/R 100/1945, 104/1945; 115/1945; Bissalanca – IICT/R 181/1945, 20/1945; Bissau – IICT/R 230/1945; Bissorã – IICT/R 303/1945; Bolama – IICT/R GB3; Marques Mano – IICT/R 136/1945; Tor– IICT/R 89–91/1945, 114/1945; Guiné-Bissau [undetermined locality] – IICT/R 6/1944, 10/1944, GB8		16
	*Toxicodryas* Hallowell, 1857	*Toxicodryas blandingii* (Hallowell, 1844)	Ilha de Bubaque – IICT/R GB2; Bissalanca – IICT/R 242/1945, 244/1945; Marques Mano Farm – IICT/R 16/1945	Manaças (1955)	4
**TOTAL NUMBER OF REPTILES SPECIMENS**					**376**

**Figure 10.**
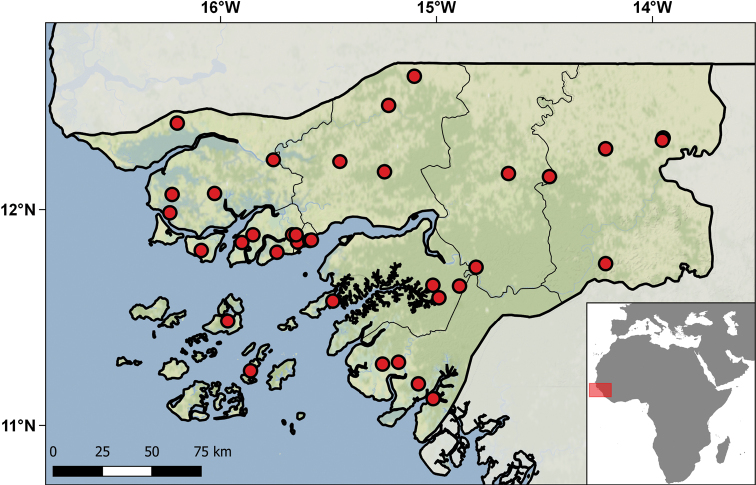
Distribution of the Guniea-Bissau localities represented in the IICT herpetological collections.

Geographically, these specimens come from 38 different localities distributed in the nine regions of the country – Bafatá, Biombo, Bissau, Bolama, Cacheu, Gabu, Oio, Quinara, and Tombali (Table 4; Fig. 10). The temporal range of collecting events ranges from 1939 to 1997, although the bulk of the specimens were collected between 1944 and 1946. The main contributors to the collections were Fernando Frade and his team during the Zoological Missions to Guinea Bissau (1944–1946), although different individuals collected other specimens on other occasions and dates. This material was primarily studied and published by Sara Manaças on five different occasions (Manaças 1947, 1949, 1950a, 1951a, b, 1955). Gans (1987) used specimens of *Cynisca feae* (Boulenger, 1906) (IICT 28/1945; 29/1945; 40/1945; 42/1945; 46/1945; 47/1945; 53/1945; 64/1945; 65/1945; 66/1945; 70/1945; 71/1945; 76/1945; 77/1945; 93/1945; 94/1945; 138/1945) in a taxonomic revision of the genus *Cynisca* (Squamata: Amphisbaenidae). Some of the snake specimens were used by Manaças (1982) to produce a checklist and identification keys for the venomous snakes of the Portuguese speaking countries in Africa. No further material was ever cited in subsequent papers and the collection has remained almost inaccessible until now. In the course of our rehousing effort, previously unpublished specimens were located, including the first confirmed record of the Gaboon Caecilian, *Geotrypetes seraphini* (Duméril, 1859) for the country (Marques and Ceríaco pers. obs.). Specimens from this collection are currently being used for ongoing taxonomic revisions and graduate students’ projects. The collection holds no type material.

**Table 4.** Gazetteer of Guinea-Bissau localities of IICT specimens. Latitude and longitude decimal coordinates are presented in WGS-84 projection.

Province	Verbatim locality	Current locality	Latitude and Longitude	Uncertainty (meters)	Elevation (meters)	Number of taxa/records
Bafatá	Bafatá	Bafatá	12.166667, -14.666667	3036	8	5/10
Bafatá	Bambadinca	Bambadinca	12.152227, -14.476879	3036	49	1/1
Bijagós	llha de Bubaque	Bubaque Island	11.252357, -15.861174	6538	24	1/1
Bijagós	Bolama	Bolama	11.57671, -15.48019	3036	16	1/1
Bijagós	Ilha Formosa	Formosa Island	11.483333, -15.966667	12350	31	2/3
Biombo	Bijimita	Bijimita	11.883333, -15.85	3036	13	6/13
Biombo	Ponta de Machado	Machado Farm	11.84958, -15.642988	10000	42	9/15
Biombo	Marques Mano	Marques Mano Farm	11.84958, -15.642988	10000	42	20/104
Biombo	Prabis	Prabis [or Prábis]	11.8025, -15.738888	1008	22	3/25
Biombo	Tor	Tor	11.847634, -15.901396	3036	18	10/45
Bissau	Bissalanca	Bissalanca	11.883333, -15.666667	3036	37	14/32
Bissau	Bissau	Bissau	11.857056, -15.58711	7327	15	7/19
Bissau	Bissau, Cidade	Bissau, city center	11.858736, -15.579213	301	14	4/8
Bissau	Brene	Brene	11.883333, -15.65	301	35	3/3
Cacheu	Caiomete	Caiomete	11.985172, -16.234847	1000	8	1/2
Cacheu	Calequisse, Canchingo	Calequisse, Chachungo	12.069722, -16.224444	900	25	4/13
Cacheu	Chachungo	Chachungo	12.074424, -16.02772	825	31	4/34
Cacheu	Pecixe	Pecixe	11.810861, -16.089651	3036	8	6/25
Cacheu	São Domingos	São Domingos	12.401944, -16.200556	3036	10	1/3
Cacheu	São Vicente	S. Vicente	12.230989, -15.755374	1566	14	1/1
Gabu	Gabu [or Nova Lamego]	Gabu	12.283333, -14.216667	2000	48	4/13
Gabu	Madina Boé	Madina do Boé	11.75, -14.216667	3036	98	2/32
Gabu	Mandinga [or Miniou, Mandinga]	Mandinga	12.618496, -15.102543	6437	48	2/2
Gabu	Nhampurbani, Pitche	Nhampurbani, Pitche	12.333333, -13.95	3036	71	1/1
Gabu	Pitche	Pitche	12.3226, -13.95412	1352	66	8/121
Oio	Bissoram	Bissorã	12.223056, -15.4475	7500	15	11/189
Oio	Cutiá, Mansoa	Cutiá, Mansoa [or Mansôa]	12.174722, -15.240833	3036	35	3/26
Oio	Farim	Farim	12.483889, -15.221667	800	11	1/29
Quinara	Buba	Buba	11.592204, -14.988713	1687	40	1/1
Quinara	Chitole	Chitole [or Xitole]	11.733333, -14.816667	877	28	3/6
Quinara	Entre Buba e o rio Coruball	between Buba and Corubal river	11.646348, -14.893414	5000	23	1/1
Quinara	Tombô-Fulacunda	Buba-Tombó	11.65, -15.01667	1094	39	
Tombali	Cachamba	Cachamba	11.191942, -15.0839	5168	18	1/1
Tombali	Cacine	Cacine	11.123447, -15.01538	1640	11	11/47
Tombali	Catió	Catió	11.283333, -15.25	301	9	8/56
Tombali	Catió - Cufer	Catió, Cufer	11.291869, -15.175909	700	9	2/8
	Guiné-Bissau	Guiné-Bissau, undetermined locality				10/13

Specimens from Guinea-Bissau are scarce in collections worldwide. The main collections holding specimens of amphibians and reptiles from Guinea-Bissau are the IICT, with 980 specimens; ZFMK, with 108 specimens (Aulyia et al. 2012); the MHNC, with 329 specimens (Monard 1940a, b), and the MNHN with 46 specimens (data retrieved from GBIF.org in February 2021). Other specimens exist in several other museums but generally not exceeding 10 specimens per collection (data retrieved from GBIF.org in March 2020).

The dataset of this collection is available at GBIF (Ceríaco and Marques 2018a; https://www.gbif.org/dataset/c6e94ce4-5d25-4758-8a52-2d5ee4d520fc).

### São Tomé & Príncipe collections

Since the early 2000's the herpetofauna of São Tomé & Príncipe has been subject of several studies, including the descriptions of various new species of amphibians and reptiles. A provisional checklist of the terrestrial herpetofauna of São Tomé, Príncipe, and Annobon was provided by Ceríaco et al. (2018a). This work listed eight species of amphibians (five for São Tomé and three for Príncipe) and 23 species of reptiles (12 for São Tomé and 13 for Príncipe). New studies have since contributed to the expansion of our knowledge of the herpetofauna of the country, including the description of a new species of the genus *Boaedon* (Ceríaco et al. 2021), and several revisions have resurrected synonymised taxa, as the case of *Schistometopum ephele* (O’Connell et al. in press). Exemplifying this constant flux of work, the recent publication of two major reviews on the herpetofauna of these islands by Bell et al. (in press) and Ceríaco et al. (2021) provide detailed historical overviews on the study of the amphibians and reptiles and updated the known number of species to eight amphibians and 22 reptiles.

The collection comprises 234 specimens (76 amphibians and 158 reptiles), eight species of amphibians and 20 species of reptiles (Table 5), corresponding to 80% of the known taxa for the country. The amphibian collection comprises representatives of five families and five genera corresponding to six anurans and one caecilian. The family Dermophiidae is the best represented with 43 individuals of the São Tomé Caecilian, *Schistometopum thomense* (Bocage, 1873), endemic to São Tomé Island. The family Phrynobatrachidae is the second best represented within the collection with a total of 15 individuals, of which nine correspond to the Príncipe Puddle Frog, *Phrynobatrachus dispar* (Peters, 1870), endemic to Príncipe Island, and six correspond to the Calm Puddle Frog, *Phrynobatrachus leveleve* Uyeda, Drewes & Zimkus, 2007, endemic to São Tomé Island. All known amphibian species are present in the collection with the exception of the Moller’s Reed Frog, *Hyperolius molleri* (Bedriaga 1892). The reptile collection comprises representatives of six families and 11 genera, all squamates. The families Scincidae, Lamprophiidae, and Colubridae are the best represented in the collection with representatives of all known taxa for the country with 76, 23, and 21 specimens respectively. The Scincidae is the family with the greatest diversity of species.

**Table 5.** Overview of the São Tomé & Príncipe’s amphibian and reptile collections of IICT.

Family	Genus	Species	Localities – Accession number	References	Number of specimens
			(* denotes a type specimen)		
**AMPHIBIANS**					
**ANURA Duméril, 1806**					
**Hyperoliidae** Laurent, 1943	*Hyperolius* Rapp, 1842	*Hyperolius drewesi* Bell, 2016	Roça Esperança – IICT/A 13A/1954		1
		*Hyperolius thomensis* Bocage, 1886	Roça Saudade – IICT/A STP6; São Tomé Island [undetermined locality] – IICT/A STP5		2
**Arthroleptidae** Mivart, 1869	*Arthroleptis* Smith, 1849	*Leptopelis palmatus* (Peters, 1868)	Príncipe Airport – IICT/A 52–53/1955, 54–55/1956; Santo António – IICT/A 13–14/1955; Ribeira Izé – IICT/A 44–45/1955	Manaças (1958)	8
**Ptychadenidae** Dubois, 1987	*Ptychadena* Boulenger, 1917	*Ptychadena newtoni* (Bocage, 1886)	Roça Monte Café – IICT/A 1/1961; São Tomé Island [undetermined locality] – IICT/A 15/1967, 16A–16B/1967		4
**Phrynobatrachidae** Laurent, 1941	*Phrynobatrachus* Günther, 1862	*Phrynobatrachus dispar* (Peters, 1870)	Príncipe Airport – IICT/A 46–51/1955; Roça Azeitona [or Roça São Jorge] – IICT/A STP7; Roça Esperança – IICT/A 15/1954, 18/1954	Manaças (1958)	9
		*Phrynobatrachus leveleve* Uyeda, Drewes & Zimkus, 2007	Roça Monte Café – IICT/A 37/1954; São Tomé Island [undetermined locality] – IICT/A 2–4/1954, STP4A, 4B		6
**GYMNOPHIONA Müller, 1832**					
**Dermophiidae** Taylor, 1969	*Schistometopum* Parker, 1941	*Schistometopum thomense* (Bocage, 1873)	Potó-Correia – IICT/A 9/1966; Roça Milagrosa – IICT/A 1/1954, 9–12/1954, 9/1966, STP3B, 3C; Roça Monte Café – IICT/A 26–41/1954; Roça Pinheira – IICT/A 1/1967; Roça Ponta-Figo – IICT/A 56–57/1958, STP3A; Roça S. Nicolau – IICT/A 5/1954; Roça Saudade – IICT/A 1–9/1963, 20–25/1954	Manaças (1958)	43
		*Schistometopum ephele* Taylor, 1965	Roça Porto Alegre – IICT/A 6–8/1954;	Manaças (1958)	3
**TOTAL NUMBER OF AMPHIBIAN SPECIMENS**					**76**
**REPTILES**					
**SQUAMATA** Oppel, 1811					
**Gekkonidae** Gray, 1825	*Hemidactylus* Goldfuss, 1820	*Hemidactylus greeffii* Bocage, 1886	Roça Nova Moca – IICT/R 12/1954, 20/1954	Manaças (1958)	2
		*Hemidactylus longicephalus* Bocage, 1873	Santo António – 67–68/1955, 104–106/1955	Manaças (1958)	5
		*Hemidactylus mabouia* (Moreau de Jonnès, 1818)	Roça Esperança – IICT/R 40/1954, 43/1954; Roça Nova Moca – IICT/R 17–19/1954, 49/1954; Roça Potó-Correia – IICT/R 16–18/1966	Manaças (1958)	8
		*Hemidactylus principensis* Miller, Sellas & Drewes, 2012	Príncipe Airport – IICT/R 102/1955	Manaças (1958)	1
		*Hemidactylus* sp.	Tinhosa Grande Islet – IICT/R 1/1970		1
	*Lygodactylus* Gray, 1864	*Lygodactylus delicatus* Pasteur, 1962	Príncipe Airport – IICT/R 64–66/1955	Manaças (1958)	3
**Scincidae** Cuvier, 1808	*Feylinia* Gray, 1845	*Feylinia polylepis* Bocage, 1887	Roça Porto Real – IICT/R 28/1954, STP9; Roça Esperança – IICT/R 30–33/1954, 42/1954, STP9	Manaças (1958)	8
	*Panaspis* Cope, 1868	*Panaspis africana* (Gray, 1845)	Príncipe Airport – IICT/R 71/1955, 76/1955, 81–98/1955	Manaças (1958)	20
		*Panaspis thomensis* Ceríaco, Soares, Marques, Bastos-Silveira, Scheinberg, Harris, Brehm & Jesus, 2018	Roça Monte Café – IICT/R 45/1954, 47–48/1954; Roça Nova Moca – IICT/R 2/1954, 50/1954	Manaças (1958); Soares et al. (2018)	5
	*Trachylepis* Fitzinger, 1843	*Trachylepis adamastor* Ceríaco, 2015	Tinhosa Grande Islet – IICT/R 1–2/1970, 1–6/1971; Príncipe Airport – IICT/R 69/1955, 73–74/1955; Roça Esperança – IICT/R 35/1954	Ceríaco (2015); Ceríaco et al. (2016)	13
**Scincidae** Cuvier, 1808	*Trachylepis* Fitzinger, 1843	*Trachylepis affinis* (Gray, 1838)	Príncipe Airport – IICT/R 70/1955, 72/1955, 77–80/1955, 99/1955; Roça Esperança – IICT/R 36–39/1954, 44/1954	Manaças (1958); Ceríaco et al. (2016)	14
		*Trachylepis thomensis* Ceríaco, Marques & Bauer, 2016	Rolas Islet – IICT/R 3–7/1954; Roça Bela Vista – IICT/R 13–16/1954; Potó-Correia – IICT/R 11–16/1966; Roça Pinheira – IICT/R STP11; São Tomé Island [undetermined locality] – IICT/R 14/1967	Manaças (1958, 1973); Ceríaco et al. (2016)	17
SERPENTES					
**Typhlopidae** Merrem, 1820	*Afrotyphlops* Broadley & Wallach, 2009	*Afrotyphlops elegans* (Peters, 1868)	Príncipe Island [undetermined locality] – IICT/R 111–112/1956; Roça Esperança – IICT/R 29/1954; Santo António – IICT/R 27/1954	Manaças (1958)	4
	*Letheobia* Cope, 1868	*Letheobia newtoni* (Bocage, 1890)	Roça Monte Café – IICT/R 22–23/1954, STP3; Roça Potó-Correia – IICT/R 7/1966, 10/1966	Manaças (1958, 1973)	5
		*Letheobia* sp.	Roça Porto Real – IICT/R 6/1967, 11–12/1967		3
**Lamprophiidae** Fitzinger, 1843	*Boaedon* Duméril, Bibron & Duméril, 1854	*Boaedon bedriagae* Boulenger, 1907	Água-Izé – IICT/R 51/1954; Angra Toldo – IICT/R 2/1967; Ponta-Figo – IICT/R STP12; Potó-Correia – IICT/R 4/1966, 18/1967, 21/1967; Roça Boa Entrada – IICT/R 1771967; Roça Porto Alegre – IICT/R 8/1954; São Tomé Island [undetermined locality] – IICT/R 3/1966, 19/1967, STP13	Manaças (1973)	11
		*Boaedon mendesi* Ceríaco, Arellano, Jadin, Marques, Parrinha & Hallermann, 2021	Príncipe Airport – IICT/R 63/1955; Roça Sundy – IICT/R 113–114/1956, 2671954, 8/1967; Santo António – IICT/R 109/1955; Príncipe Island [undetermined locality] – IICT/R 52–56/1955, 58–59/1955	Manaças (1958, 1973)	12
**Elapidae** Boie, 1827	*Naja* Laurenti, 1768	*Naja peroescobari* Ceríaco, Marques, Schmitz & Bauer, 2017	Ribeira Peixe – IICT/R 18/1972; Roça Porto Alegre – IICT/R 9/1954; Santa Josefina – IICT/R 20/1967; São Tomé Island [undetermined locality] – IICT/R 2/1966; Uba-Budo – IICT/R STP10	Manaças (1958, 1973); Ceríaco et al. (2017)	5
**Colubridae** Oppel, 1811	*Hapsidophrys* Fischer, 1856	*Hapsidophrys principis* (Boulenger, 1906)	Príncipe Airport – IICT/R 103/1955, 107–108/1955; Roça São Jorge [or Roça Azeitona] – IICT/R 61–62/1955; Roça Sundy – IICT/R 41/1954; Príncipe Island [undetermined locality] – IICT/R 60–61/1955, 110/1956, STP2	Manaças (1958)	10
	*Philothamnus* Smith, 1840	*Philothamnus thomensis* Bocage, 1882	Água-Izé – IICT/R 10/1954; Angra Toldo – IICT/R 3/1967; Ponta-Figo – IICT/R 115–117/1958; Roça Potó-Correia – IICT/R 5–8/1966, 13/1967; Roça Milagrosa – IICT/R 21/1954; São Tomé Island [undetermined locality] – IICT/R STP1	Manaças (1958, 1973)	11
**TOTAL NUMBER OF REPTILES SPECIMENS**					**158**

**Figure 11.**
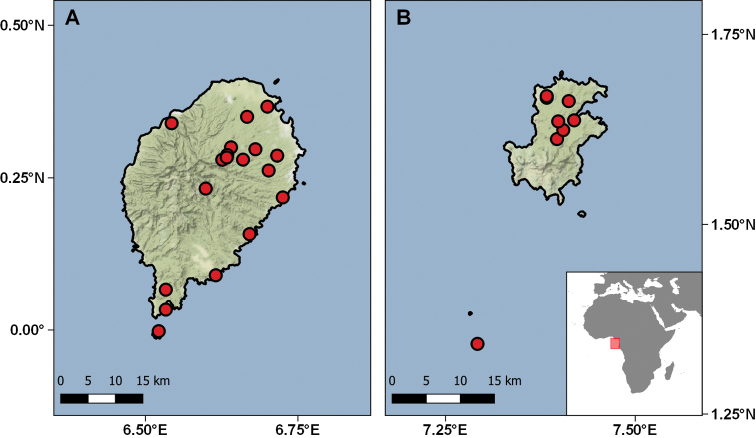
Distribution of the São Tomé (**A**) & Príncipe (**B**) localities represented in the IICT herpetological collections.

Geographically these specimens come from 23 different localities distributed in the two islands, São Tomé and Príncipe, and in two small islets on each coast of the main islands, respectively, Rolas and Tinhosa Grande (Table 6; Fig. 11). The temporal range of collecting events is from 1954 to 1971, although the bulk of the specimens were collected in the years 1954, 1956, and 1966. The main contributors to the collections were Fernando Frade and his team during the Scientific Mission to São Tomé and Príncipe (1954), and Décio Passos (birth and death dates unknown), an airport worker in Príncipe, who collected and offered a good series of specimens during 1955 and 1956. This material was primarily studied and published by Sara Manaças on two different occasions (Manaças 1958, 1973). In recent years the importance of this collection has increased with the descriptions of six new species, all based on the IICT material (Ceríaco 2015; Ceríaco et al. 2016, 2017, 2021a; Soares et al. 2018), which have resulted in a good series of types in the collection, including the Adamastor Skink, *Trachylepis adamastor* Ceríaco, 2015 (holotype and seven paratypes), the São Tomé Leaf-litter Skink, *Panaspis thomensis* Ceríaco, Soares, Marques, Bastos-Silveira, Scheinberg, Harris, Brehm & Jesus, 2018 (two paratypes), the São Tomé Cobra, *Naja peroescobari* Ceríaco, Marques, Schmitz & Bauer, 2017 (three paratypes), and the Príncipe Jita Snake, *Boaedon mendesi* Ceríaco, Arellano, Jadin, Marques, Parrinha & Hallermannn, 2021 (five paratypes). These specimens have also been used in phylogeographic studies (Ceríaco et al. 2020c).

**Table 6.** Gazetteer of São Tomé & Príncipe localities of IICT specimens. Latitude and longitude decimal coordinates are presented in WGS-84 projection.

Island/Islet	Verbatim locality	Current locality	Latitude and Longitude	Uncertainty (meters)	Elevation (meters)	Number of taxa/records
Príncipe	Aeroporto	Príncipe Airport	1.662259, 7.41235	1039	180	9/50
Príncipe	Roça Azeitona	Roça Azeitona [or Roça São Jorge]	1.666667, 7.383333	301	171	2/3
Príncipe	Ilha do Príncipe [or Príncipe]	Príncipe Island	1.612248, 7.396894	11813	149	3/12
Príncipe	Porto Real [or Roça Porto Real]	Roça Porto Real	1.624118, 7.405149	656	132	2/5
Príncipe	Roça Esperança	Roça Esperança	1.635551, 7.398481	154	188	7/18
Príncipe	Roça Sundy	Roça Sundy	1.668775, 7.383353	852	169	2/5
Príncipe	Santo António	Santo António	1.636944, 7.419444	790	12	4/9
São Tomé	Água-Izé [or Ribeira Izé]	Água-Izé [or Ribeira Izé]	0.217868, 6.725149	210	38	3/4
São Tomé	Angra Toldo	Angra Toldo	0.15770, 6.67070	301	19	2/2
São Tomé	Ponta-Figo [or Roça Ponta-Figo]	Ponta-Figo [or Roça Ponta-Figo]	0.339465, 6.54286	920	151	3/7
São Tomé	Potó-Correia [or Roça Potó-Correia]	Potó-Correia [or Roça Potó-Correia]	0.29685, 6.680288	1000	291	6/18
São Tomé	Ribeira Peixe	Ribeira Peixe	0.09028, 6.61528	301	19	1/1
São Tomé	Roça Bela Vista	Roça Bela Vista	0.366667, 6.7	3036	27	1/4
São Tomé	Roça Boa Nova	Roça Boa Entrada	0.35, 6.666667	3036	180	1/1
São Tomé	Roça Milagrosa	Roça Milagrosa	0.27978, 6.65995	211	465	2/8
São Tomé	Roça Monte-Café	Roça Monte Café	0.299931, 6.64031	311	694	5/23
São Tomé	Roça Nova Moca	Roça Nova Moca	0.287436, 6.63414	339	854	3/8
São Tomé	Pinheira [or Pinheiro]	Roça Pinheira	0.286313, 6.716026	44	118	2/2
São Tomé	Roça Porto Alegre	Roça Porto Alegre	0.033333, 6.533333	634	16	3/5
São Tomé	Roça S. Nicolau	Roça S. Nicolau	0.279608, 6.625934	427	917	1/1
São Tomé	Roça Saudade	Roça Saudade	0.283333, 6.633333	3036	771	2/16
São Tomé	Santa Josefina	Santa Josefina	0.066667, 6.533333	3036	109	1/1
São Tomé	Uba-Budo	Uba-Budo	0.262, 6.702	333	254	1/1
São Tomé	São Tomé	São Tomé Island	0.232334, 6.598798	25289	695	7/15
Rolas	Ilhéu das Rolas	Rolas Islet	-0.002066, 6.521748	1365	89	1/5
Tinhosa Grande	Pedras Tinhosas	Tinhosa Grande Islet	1.342118, 7.292135	117	55	2/9

The main collections holding specimens of amphibians and reptiles from São Tomé & Príncipe are CAS with 1562 specimens, followed by UMMZ with 1106, and USNM with 778 specimens (data retrieved from GBIF.org in February 2021). The IICT collection, together with the collection hosted at MUHNAC, is the fourth largest collection with a total of 457 specimens.

The dataset of this collection is available at GBIF (Ceríaco and Marques 2018b; https://www.gbif.org/dataset/c6e94ce4-5d25-4758-8a52-2d5ee4d520fc).

### Angola collection

After approximately four decades of violent armed conflict that closed the country to researchers, the study of Angolan herpetofauna has experienced a rapid growth in the last decade. The most recent checklists of the herpetofauna of Angola report 117 species of amphibians and 278 reptiles, of which 54 are endemic to the country (Marques et al. 2018; Baptista et al. 2019; Branch et al. 2019). However, the available checklists are rapidly becoming outdated due to the rapid growth of species discovery and new species descriptions (see Branch 2018; Marques et al. 2019a, b, 2020; Ceríaco et al. 2020a, b; Hallermannn et al. 2020). Marques et al. (2018) provide a comprehensive review of the history of herpetological research in Angola.

The collection comprises 677 specimens (259 amphibians and 418 reptiles), covering 21 species of amphibians and 45 species and 2 subspecies of reptiles (Table 7). A total of ten families of amphibians are represented in the collection comprising eleven different genera, all anurans. Ptychadenidae is the best represented family with 45 specimens of the multiple cryptic group of Mascarene Grass Frog, Ptychadena cf. mascareniensis; 16 specimens of Spotted Grass Frog, *Ptychadena subpunctata* (Bocage, 1866); 17 specimens of a series of tadpoles and juveniles of an unidentified Grass Frog *Ptychadena* sp.; two specimens of Grandison’s Grass Frog, *Ptychadena grandisonae* Laurent, 1954; and a single specimen of both Anchieta’s Grass Frog, *Ptychadena anchietae* (Bocage, 1868) and Upemba Grass Frog, *Ptychadena upembae* (Schmidt & Inger, 1959). These are followed by the families Hyperolidae with a series of 91 tadpoles and juveniles of an unidentified Reed Frog *Hyperolius* sp., two specimens of the Angolan Reed Frog *Hyperolius angolensis* Steindachner, 1867, and Phrynobatrachidae with 48 specimens of the Natal Dwarf Puddle Frog *Phrynobatrachus natalensis* (Smith, 1849). These three families make up almost 80% of the amphibian specimens. The reptile collection is taxonomically more diverse, comprising a total of 15 families corresponding to a total of 31 genera. Chelonians are represented by a single family, Pelomedusidae, while all other families are squamates. Among the squamates (non-snakes) the Scincidae is the best represented family with 124 specimens of four different genera, including *Trachylepis* (102 specimens), *Sepsina* (16 specimens), *Panaspis* (four specimens), and *Lubuya* (two specimens); followed by the family Agamidae with 51 specimens of two different genera, *Acanthocercus* (28 specimens) and *Agama* (23 specimens), and the families Chamaeleonidae (41 specimens) and Lacertidae (31 specimens), both represented by a single species, the Quilo Flap-Neck Chamaeleon, *Chamaeleo dilepis quilensis* Bocage, 1886 and the Angolan Rough-Scaled Lizard *Ichnotropis bivittata* Bocage, 1866, respectively. This collection includes a good diversity of snakes representative of the country, comprising six different families, Typhlopidae, Viperidae, Lamprophiidae, Elapidae, Colubridae, and Natricidae, representing 31% of the collection.

**Table 7.** Overview of the Angola amphibian and reptile collections of IICT.

Family	Genus	Species	Localities – Accession number	References	Number of specimens
			(* denotes a type specimen)		
**AMPHIBIANS**					
**ANURA Duméril, 1806**					
**Pipidae** Gray, 1825	*Xenopus* Wagler, 1827	*Xenopus petersii* Bocage, 1897	Calandula [or Kalandula] waterfalls – IICT/A 1/1957; Lucusse – IICT/A 18/1957; Calombe river – IICT/A 27/1959	Ruas (2002a)	3
**Bufonidae** (Gray, 1825)	*Sclerophrys* Tschudi, 1838	*Sclerophrys garmani* (Meek, 1897)	Calombe river – IICT/A 168/1959	Ruas (2002a)	1
		*Sclerophrys gutturalis* (Power, 1927)	Calombe river – IICT/A 155/1959, IICT/A 166/1959; Caluando river, Luando Reserve – IICT/A 66–67/1958; Dilolo lake – IICT/A 64–65/1958; Lubango – IICT/A 55–1957; Luena – IICT/A 14/1957, IICT/A 17/1957; Luvuei – IICT/A 20/1957	Ruas (2002a)	10
		*Sclerophrys pusilla* (Mertens, 1937)	Calandula [or Kalandula] waterfalls – IICT/A 2/1957	Ruas (2002a)	1
		*Sclerophrys* sp.	Calombe river – IICT/A 151/1959	Ruas (2002a)	1
	*Poyntonophrynus* Frost, Grant, Faivovich, Bain, Haas, Haddad, de Sá, Channing, Wilkinson, Donnellan, Raxworthy, Campbell, Blotto, Moler, Drewes, Nussbaum, Lynch, Green & Wheeler, 2006	*Poyntonophrynus kavangensis* Poynton & Broadley, 1988	Calombe – IICT/A 156/1959	Ruas (2002a)	1
**Brevicipitidae** Bonaparte, 1850	*Breviceps* Merrem, 1820	*Breviceps poweri* Parker, 1936	Calombe – IICT/A 163/1959, 169/1959, 174–177/1959	Ruas (2002a)	6
**Hemisotidae** Cope, 1867	*Hemisus* Günther, 1859	*Hemisus marmoratus* (Peters, 1854)	Calombe – IICT/A 150/1959, 171/1959		2
**Hyperoliidae** Laurent, 1943	*Hyperolius* Rapp, 1842	*Hyperolius angolensis* Steindachner, 1868	Caconda – IICT/A 54/1957; Cameia lake – IICT/A 10-1958		2
		*Hyperolius* sp.	Calombe – IICT/A 100–112/1959, 152/1959; Calombe river – IICT/A 28–84/1959; Caluando river, Luando Reserve – IICT/A 26/1959; Saurimo – IICT/A 6/1957; Angola [undetermined locality] – IICT/A 147/1959		91
**Arthroleptidae** Mivart, 1869	*Arthroleptis* Smith, 1849	*Arthroleptis stenodactylus* Pfeffer, 1893	Calombe – IICT/A 172–173/1959	Ruas (2002a)	2
**Ptychadenidae** Dubois, 1987	*Ptychadena* Boulenger, 1917	*Ptychadena anchietae* (Bocage, 1868)	Dilolo lake – IICT/A 62/1958	Ruas (2002a)	1
		*Ptychadena grandisonae* Laurent, 1954	Calombe river – IICT/A 113/1959; Luena – IICT/A 15/1957	Ruas (2002a)	2
		Ptychadena cf. mascareniensis (Duméril & Bibron, 1841)	Cameia lake – IICT/A 2–8/1958, 15–37/1958; Caluando river, Luando Reserve – IICT/A 17–20/1959, 22–23/1959; Dilolo lake – IICT/A 55–61/1958; Kwanza river margins, Luando Reserve – IICT/A 16-1959	Ruas (2002a)	45
		*Ptychadena subpunctata* (Bocage, 1866)	Cameia lake – IICT/A 1/1958, 38–50/1958; Dilolo lake – IICT/A 53/1958, 63/1958	Ruas (2002a)	16
		*Ptychadena* sp.	Calandula [or Kalandula] waterfalls – IICT/A 7–13/1957, 11/1958; Calombe – IICT/A 160–162/1959; Cameia lake – IICT/A 52/1958; Luena – IICT/A 16/1957; Angola [undetermined locality] – IICT/A 3/5/1957, 53/1957		17
		*Ptychadena upembae* (Schmidt & Inger, 1959)	Cameia lake – IICT/A 14/1958	Ruas (2002a)	1
**Ptychadenidae** Dubois, 1987	*Ptychadena* Boulenger, 1917	*Ptychadena uzungwensis* (Loveridge, 1932)	Calombe – IICT/A 167/1959	Ruas (2002a)	1
**Phrynobatrachidae** Laurent, 1941	*Phrynobatrachus* Günther, 1862	*Phrynobatrachus natalensis* (Smith, 1849)	Calombe river – IICT/A 144–145/1959; Camanongue – IICT/A 21–52/1957; Kwanza river source, Luando Reserve – IICT/A 1/1959, 10–13/1959; Kwanza river margins, Luando Reserve – IICT/A 2–9/1959, 21/1959;	Ruas (2002a)	48
**Pyxicephalidae** Bonaparte, 1850	*Amietia* Dubois, 1987	*Amietia angolensis* (Bocage, 1866)	Calombe – IICT/A 164–165/1959; Luena – IICT/A 21A/1957	Ruas (2002a)	3
**Ranidae** Batsch, 1796	*Amnirana* Dubois, 199	*Amnirana darlingi* (Boulenger, 1902)	Calombe – IICT/A 154/1959, 157/1959, 159/1959; Angola [undetermined locality] – IICT/A – 148–149/1959	Ruas (2002a)	5
**TOTAL NUMBER OF AMPHIBIAN SPECIMENS**					**259**
**REPTILES**					
**CHELONII** Brongiart, 1800					
**Pelomedusidae** Cope, 1868	*Pelomedusa* Wagler, 1830	*Pelomedusa subrufa* (Lacepède, 1788)	Angola [undetermined locality] – IICT/R 4/1958		1
	*Pelusios* Wagler, 1830	*Pelusios bechuanicus* FitzSimons, 1933	Angola [undetermined locality] – IICT/R 1/1958, 1A/1958, 2A/1958, 3/1958		4
**SQUAMATA** Oppel, 1811					
**Gekkonidae** Gray, 1825	*Hemidactylus* Goldfuss, 1820	*Hemidactylus longicephalus* Bocage, 1873	Quizambil mines – IICT/R 1–3/1957	Manaças (1963); Ceríaco, Agarwal, Marques and Bauer (2020)	3
		*Hemidactylus nzingae* Ceríaco, Agarwal, Marques & Bauer, 2020	Candande farm – IICT/R 4–5/1957	Manaças (1963); Ceríaco, Agarwal, Marques and Bauer (2020)	2
**Amphisbaenidae** Gray, 1825	*Dalophia* Gray, 1865	*Dalophia angolensis* Gans, 1976	Calombe, 7 km “west” from Luena – IICT/R 50/1959, 167/1959, 204/1959, 265/1959, 294–296/1959, 298–300/1959, 318/1959, 387/1959, 399/1959, 430/1959	Gans (1976)	16
	*Zygaspis* Cope, 1885	*Zygaspis nigra* Broadley & Gans, 1969	Calombe, 7 km “west” from Luena – IICT/R 44/1959, 203/1959, 314–316/1959, 339A/1959		6
**Varanidae** Hardwicke & Gray, 1824	*Varanus* Merrem, 1820	*Varanus niloticus* (Linnaeus, 1766)	Dilolo lake – IICT/R 108/1958	Manaças (1963)	1
**Lacertidae** Bonaparte, 1831	*Ichnotropis* Peters, 1854	*Ichnotropis bivittata* Bocage, 1866	Luena – IICT/R 136–137/1958; Luena (Santa Cruz farm) – IICT/R 229–231/1959; Cameia lake – IICT/R 5/1958; Calombe – IICT/R 58–60/1959, 79–81/1959, 244/1959, 252/1959, 267/1959, 277/1959, 279/1959, 320–323/1959, 339/1959, 374–375/1959, 410/1959, 420/1959, 437/1959; Angola [undetermined locality] – IICT/R Angola6–9	Manaças (1963)	31
**Scincidae** Cuvier, 1808	*Lubuya* Horton, 1972	*Lubuya ivensii* (Bocage, 1879)	Calombe – IICT/R 48/1959, 239/1959	Manaças (1963)	2
	*Panaspis* Cope, 1868	*Panaspis* sp.	Calombe – IICT/R 268/1959, 276–277/1959, 288/1959		4
	*Sepsina* Bocage, 1866	*Sepsina angolensis* Bocage, 1866	Calombe – IICT/R 47/1959, 79A–81A/1959, 200–201/1959, 264/1959, 386/1959, 409/1959, 416/1959, 431–432/1959, 436/1959, 438–439/1959, Angola1		16
**Scincidae** Cuvier, 1808	*Trachylepis* Fitzinger, 1843	*Trachylepis bayonii* (Bocage, 1872)	Calombe – IICT/R 243/1959, 246/1959, 254–255/1959, 258/1959, 268–276/1959, 278/1959, 288/1959, 324–325/1959, 327–338/1959, 364–373/1959, 376–385/1959, 378A/1959, 385A/1959; 421–425/1959, 427–428/1959; Angola [undetermined locality] – IICT/R Angola18	Manaças (1963)	60
		*Trachylepis* sp.	Cameia lake – IICT/R 30–31/1958		2
		*Trachylepis wahlbergii* (Peters, “1869” 1870)	Kuito – IICT/R 77/1957, 81/1957; Cameia lake – IICT/R 23/1958, 26/1958, 232/1959; Calombe – IICT/R 61–67/1959, 253/1959, 256–257/1959, 259/1959, 280/1959, 283/1959, 326/1959, 335/1959, 405/1959, 426/1959; Dilolo lake – IICT/R 79–84/1959, 124–125/1958; Luena – IICT/R 234–235/1959, 238/1959; Santa Cruz farm, Luena – IICT/R 138/1958; Angola [undetermined locality] – IICT/R Angola10–16	Manaças (1963)	40
**Gerrhosauridae** Fitzinger, 1843	*Gerrhosaurus* Wiegmann, 1828	*Gerrhosaurus bulsi* Laurent, 1954	Cameia lake – IICT/R 122/1958; Dilolo lake – IICT/R 2/1958, 72/1958	Manaças (1963)	3
	*Tetradactylus* Merrem, 1820	*Tetradactylus ellenbergeri* (Angel, 1922)	Calombe – IICT/R 310–313/1959, 319/1959		5
**Agamidae** Gray, 1827	*Acanthocercus* Fitzinger, 1843	*Acanthocercus cyanocephalus* (Falk, 1925)	Cachingues – IICT/R 83/1957; Cacolo – IICT/R 15–18/1959; Calombe – IICT/R 53–55/1959, 69/1959, 251/1959, 261/1959, 291/1959, 302/1959, 391/1959, 395/1959; Cassamba – IICT/R 40/1957; Luchazes – IICT/R 38/1957; Luena – IICT/R 28/1957, 33/1957; Lumbala Nguimbo – IICT/R 36–37/1957, Kuito – IICT/R 78/1957; Santa Cruz farm, Luena – IICT/R 133–135/1959, 139–141/1959	Manaças (1963)	28
	*Agama* Daudin, 1802	*Agama aculeata* Merrem, 1820	Cameia lake – IICT/R 27–28/1958; Calombe – IICT/R 57/1959, 68/1959, 70/1959, 262–263/1959, 360–361/1959, 401/1959, 407/1959, 449/1959, 450A/1959; Cassamba – IICT/R 39-1957; Huambo – IICT/R 86/1957	Manaças (1963)	15
		*Agama shacki* Mertens, 1938	Calandula [or Kalandula] waterfalls – IICT/R 32/1959, 32A/1959; outskirts of Huambo – IICT/R 84–85/1957		4
		*Agama* sp.	Kwanza river waterfalls – IICT/R 8–11/1957	Manaças (1963)	4
**Chamaeleonidae** Gray, 1825	*Chamaeleo* Laurenti, 1768	*Chamaeleo dilepis* Leach, 1819	Cacolo – IICT/R 19/1957; Cameia lake – IICT/R 24/1958, 74/1958; Calombe – IICT/R 56/1959, 71/1959, 354–356/1959, 396–397/1959, 402–403/1959, 408/1959, 411/1959, 413–414/1959, 451/1959, 454–455/1959; Dilolo lake – IICT/R 85–96/1958, 123/1958; Luena (Santa Cruz farm) – IICT/R 131 132/1958, 146/1958, 146A/1958, 149/1959, 149A/1959; Luena –IICT/R 42/1959, 241/1959; Mutumbo – IICT/R 82-1957	Manaças (1963	41
SERPENTES					
**Typhlopidae** Merrem, 1820	*Afrotyphlops* Broadley & Wallach, 2009	*Afrotyphlops angolensis* (Bocage, 1866)	Calombe Reserve – IICT/R 415A/1959, 416A/1959, 429/1959; Kwanza river source, Luando Reserve – IICT/R 34/1959		4
		*Afrotyphlops* sp.	Dundo – IICT/R Angola17		1
**Viperidae** Oppel, 1811	*Bitis* Gray, 1842	*Bitis arietans* (Merrem, 1820)	Luena – IICT/R Angola5		1
		*Bitis gabonica* (Duméril, Bibron & Duméril, 1854)	Moxico – IICT/R A-1966		1
**Viperidae** Oppel, 1811	*Causus* Wagler, 1830	*Causus bilineatus* Boulenger, 1905	Cameia lake – IICT/R 12A/1958; Calombe – IICT/R 249/1959	Manaças (1973 “1974”)	2
		*Causus rhombeatus* (Lichtenstein, 1823)	Calombe – IICT/R 151–152/1958, 51/1959, 52A/1959, 209/1959, 290/1959, 304/1959, 306/1959, 309/1959, 386A/1959, 433/1959, 453/1959, 479/1959; Camanongue – IICT/R 75/1957; Cameia lake – IICT/R 75/1958; Cazombe river source – IICT/R 52/1959; Dilolo lake – IICT/R 126/1958; Luena (Santa Cruz farm) – IICT/R 145/1958; Luena – IICT/R 8/1959, Angola4, Angola19; Angola [undetermined locality] – IICT/R 28/1959, 31/1959, 33A/1959, 38/1959, 345/1959, 384A/1959, 410B/1959, 437A/1959, 438A/1959, 441/1959	Manaças (1973 “1974”)	31
**Lamprophiidae** Fitzinger, 1843	*Boaedon* Duméril, Bibron & Duméril, 1854	*Boaedon fradei* Hallermann et al. 2020	Calombe – IICT/R 150/1958, 5/1959, 40/1959, 341/1959, 350/1959, 400/1959, 404/1959, 410A/1959, 450/1959, 340/1968; Luena – IICT/R 240/1968	Manaças (1973 “1974”); Hallermannn et al. (2020)	11
	*Lycophidion* Fitzinger, 1843	*Lycophidion capense* (Smith, 1831)	Calombe – IICT/R 292/1959, 448/1959		2
	*Psammophis* Boie, 1825	*Psammophis angolensis* (Bocage, 1872)	Cameia lake – IICT/R 7/1958		1
		*Psammophis mossambicus* Peters, 1882	Calombe – IICT/R 29/1959, 75/1959, 77/1959, 18/1959, 245/1959, 431A/1959, 443/1959, 452/1959; Cameia National Park – IICT/R 77/1958; Dilolo lake – IICT/R 127/1958; Luena – IICT/R Angola20; Angola [undetermined locality] – IICT/R 12/1958, 14/1958	Manaças (1973 “1974”)	13
		*Psammophis zambiensis* Hughes & Wade, 2002	Calombe – IICT/R 303/1959; Cameia lake – IICT/R 30A/1958	Manaças (1973 “1974”)	2
		*Psammophylax acutus* (Günther, 1888)	Calombe – IICT/R 210–227/1959, 285/1959, 434/1959, 444/1959	Manaças (1973 “1974”)	21
		*Psammophylax* sp.	Menongue – IICT/R 15/1958		1
	*Prosymna* Gray, 1849	*Prosymna angolensis* Boulenger, 1915	Milando – IICT/R 14/1957; Angola [undetermined locality] – IICT/R 348–349, 348–349/1959		5
**Elapidae** Boie, 1827	*Pseudohaje* Günther, 1858	*Pseudohaje goldii* (Boulenger, 1895)	Luachimo dam canal – IICT/R Angola3		1
**Colubridae** Oppel, 1811	*Crotaphopeltis* Fitzinger, 1843	*Crotaphopeltis barotseensis* Broadley, 1968	Dilolo lake – IICT/R 362/1959		1
		*Crotaphoepltis hotamboeia* (Laurenti, 1768)	Calombe – IICT/R 109–110/1958; Dilolo lake – IICT/R 362/1959, 406/1959, 417/1959	Manaças (1973 “1974”)	4
	*Dasypeltis* Wagler, 1830	*Dasypeltis scabra* (Linnaeus, 1758)	Calombe – IICT/R 447/1959	Manaças (1973 “1974”)	1
	*Dipsadoboa* Günther, 1858	*Dipsadoboa shrevei* (Loveridge, 1932)	Calombe – IICT/R 4/1959		1
	*Dispholidus* Duvernoy, 1832	*Dispholidus typus typus* (Smith, 1828)	Calombe – IICT/R 76/1959, 457–458/1959; Luena (Santa Cruz farm) – IICT/R 142/1958, 147–148/1958; Luena – IICT/R 386/1958, 1–2/1959, 386/1968	Manaças (1973 “1974”)	9
		*Dispholidus typus punctatus* Laurent, 1955	Calombe – IICT/R 76/1959	Manaças (1973 “1974”)	1
**Colubridae** Oppel, 1811	*Philothamnus* Smith, 1840	*Philothamnus angolensis* Bocage, 1882	Candande farm – IICT/R 7/1957; Kuito – IICT/R 79/1957	Manaças (1973 “1974”)	2
		*Philothamnus heterolepidotus* (Günther, 1863)	Calombe – IICT/R 78/1958; Dilolo lake – IICT/R 121/1958, 130/1958; Lungué-Bungo river [or Lungwebungu] margins – IICT/R 41/1957, 247/1957; Uíge – IICT/R 17/1958	Manaças (1973 “1974”)	6
**Natricidae** Bomaparte, 1838	*Limnophis* Günther, 1865	*Limnophis bangweolicus* (Mertens, 1936)	Cameia lake – IICT/R 25/1958; Dilolo lake – IICT/R 107/1958, 251/1958		3
		*Limnophis bicolor* Günther, 1868	Calombe – IICT/R 33/1959, 45/1959; Luena – IICT/R 37/1959; Forest patch, Luena – IICT/R 35/1959		4
	*Natriciteres* Loveridge, 1953	*Natriciteres* sp.	Angola [undetermined locality] – IICT/R 16/1958		1
**TOTAL NUMBER OF REPTILES SPECIMENS**					**418**

**Figure 12.**
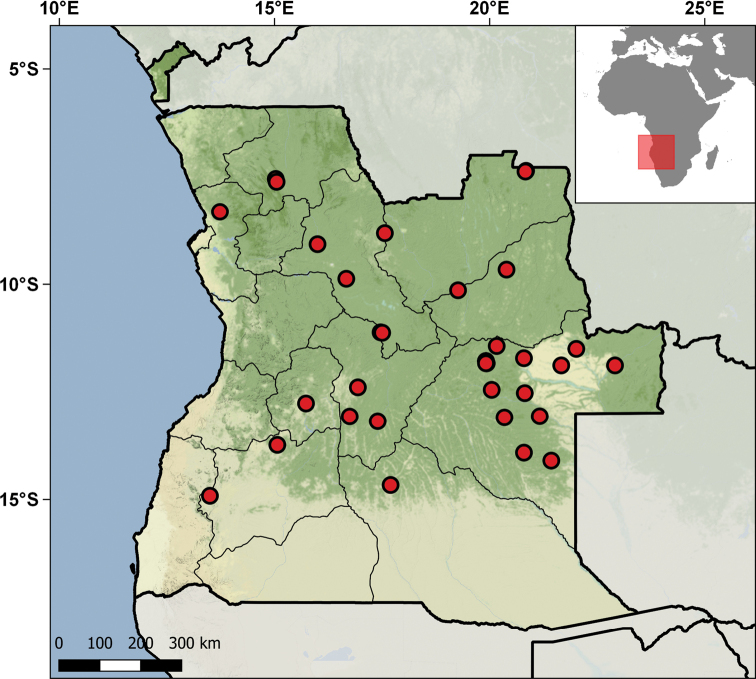
Distribution of the Angola localities represented in the IICT herpetological collections.

The geographic range of this collection covers 38 different localities from 10 different provinces: Bengo, Bié, Cuando-Cubango, Huambo, Huíla, Lunda Norte, Lunda Sul, Malanje, Moxico, and Uíge (Table 8; Fig. 12). Moxico is the best represented province, with 574 records from 14 different localities, while Lunda Sul is the least sampled with only one record. Collecting events took place between 1957 and 1968, although most of the material was collected between 1957 and 1959 during the Apiary research mission to Angola organised by the CZL, especially aimed at the study of bees and honey production.

**Table 8.** Gazetteer of Angola localities of IICT specimens. Latitude and longitude decimal coordinates are presented in WGS-84 projection.

Province	Verbatim locality	Current locality	Latitude and Longitude	Uncertainty (meters)	Elevation (meters)	Number of taxa/records
Bengo	Minas do Quizambil	Quizambil mines	-8.3166667, 13.733333	301	262	1/3
Bié	Cachingues	Cachingues	-13.071851, 16.749528	954	1713	1/1
Bié	Mutumbo	Mutumbo	-13.183333, 17.4	430	1452	1/1
Bié	Silva Porto	Kuito	-12.391968, 16.93867	2060	1716	3/4
Cuando Cubango	Menongue?	Menongue	-14.666667, 17.7	3060	1378	1/1
Huambo	Nova Lisboa [or arredores de Nova Lisboa]	Huambo [or outskirts of Huambo]	-12.766667, 15.733333	5000	1719	2/3
Huíla	Colonata de Caconda	Caconda	-13.735583, 15.066912	1600	1670	1/1
Huíla	Sá da Bandeira	Lubango	-14.916667, 13.5	6100	1751	1/1
Lunda Norte	Cacolo, Mennungo [or Cacolo, Minungo]	Cacolo	-10.14172, 19.267969	1832	1351	2/5
Lunda Norte	Canal da Barragem de Luachimo	Luachimo Dam canal	-7.361733, 20.84899	980	643	1/1
Lunda Norte	Dundo	Dundo	-7.377554, 20.835054	2240	731	1/1
Lunda Norte	Vila Henrique Carvalho	Saurimo	-9.662523, 20.39485	5000	1075	1/1
Malanje	Quedas do Duque de Bragança	Calandula [or Kalandula] waterfalls	-9.073248, 16.001402	800	1064	4/12
Malanje	Posto do Milando	Milando	-8.8166667, 17.566667	600	666	1/1
Moxico	Calombe-Luso, Moxico	Calombe	-11.833333, 19.933333	3060	1345	37/249
Moxico	Calombe (7 km de Vila Luso)	Calombe, 7 km “west” from Luena	-11.833333, 19.933333	3060	1345	2/20
Moxico	Cameia	Cameia National Park	-11.883333, 21.666667	10000	1092	1/1
Moxico	Cascata do Rio Cuanza, Cangandala	Kwanza River waterfall	-9.874712, 16.670194	3060	1086	1/4
Moxico	Cassamba	Cassamba	-13.093312, 20.346395	2500	1345	2/2
Moxico	Fazenda Santa Cruz, Luso	Santa Cruz farm, Luena	-11.783333, 19.916667	3060	1337	6/19
Moxico	Lago Cameia	Cameia lake	-11.716667, 20.8	980	1110	16/63
Moxico	Lago Dilolo	Dilolo lake	-11.5, 22.016667	4350	1088	14/44
Moxico	Lucusse	Lucusse	-12.526722, 20.818813	1758	1144	1/1
Moxico	Vila Gago Coutinho	Lumbala Nguimbo	-14.101654, 21.435308	2051	1122	1/2
Moxico	Luvuei	Luvuei	-13.066667, 21.166667	1132	1136	1/1
Moxico	Margens do Rio Lungué	Lungué-Bungo river [or Lungwebungu] margins	-12.45, 20.05	1300	1184	1/2
Moxico	Mata da Horta dos Padres, Luso	Horta dos Padres woods, Luena	-11.783333, 19.916667	3060	1337	1/1
Moxico	Moxico	Moxico [undetermined locality]	–	–	–	1/1
Moxico	Nascente do Rio Cazombe, Luso	Cazombe river source	-11.883333, 22.916667	3060	1116	1/1
Moxico	Vila Luso [or Luso];	Luena	-11.783333, 19.916667	3060	1337	14/26
Moxico	Polígono Florestal, Luso	Forest patch, Luena	-11.783333, 19.916667	10000	1330	1/1
Moxico	Posto do Bussaco	Camanongue	-11.433333, 20.166667	2000	1258	2/33
Moxico	Reserva da Palanca Preta, Nascente do Rio Quanza	Kwanza river source, Luando Reserve [or Kwanza river margins]	-11.116667, 17.46666667	3060	1191	2/14
Moxico	Rio Caluando (Reserva da Palanca Preta)	Caluando river, Luando Reserve	-11.126218, 17.504279	5000	1174	4/10
Moxico	Rio Calombe [Rio Calombe (Reserva da Palanca Preta)]	Calombe river	-11.833333, 19.916667	1370	1349	9/96
Moxico	Serra Luchazes	Luchazes	-13.9166667, 20.8	600	1271	1/1
Uíge	Fazenda Otília, Enconge	Candande Farm	-7.55, 15.03	980	763	2/3
Uíge	Uíge	Uíge	-7.6166667, 15.05	4829	825	1/1
–	Angola	Angola [undetermined locality]	–	–	–	12/41

Part of the snake and lizard collections was studied and published by Manaças (1963, 1973 “1974”, 1982), while the amphibians were studied by Ruas (1996, 2002a). In 1976 the American herpetologist Carls Gans (1923–2009) described a new species of amphisbaenian, the Angolan Worm Lizard, *Dalophia angolensis*, based on a series of specimens from this collection (Gans 1976). Several other well-known herpetologists such as Donald G. Broadley (1932–2016) and Barry Hughes (1935–to date) used the collection for their own research and provided important contributions to specimen identification. More recently the collection has been used in several projects, including new species descriptions (Ceríaco et al. 2020a, b; Hallermannn et al. 2020).

The collection holds type material for several species: *Dalophia angolensis* Gans, 1976 (holotype and 18 paratypes); Queen Nzinga’s Tropical Gecko, *Hemidactylus nzingae* Ceríaco, Agarwal, Marques & Bauer, 2020 (two paratypes); and Frade’s Brown House Snake, *Boaedon fradei* Hallermannn, Ceríaco, Schmitz, Ernst Conradie, Verburgt, Marques & Bauer, 2020 (five paratypes). The IICT holds one of the largest known herpetological collections from Angola, with 677 specimens. Currently, the largest collection of Angolan amphibians and reptiles is held by MD, with a total of 2753 specimens (Ceríaco et al. 2020d), followed by the collections of AMNH with a total of 2280 specimens, the CAS with 1347 specimens, the TM with 935 specimens, and the CM with 806 specimens (data retrieved from GBIF.org in February 2021). Other collections, as is the case of PEM in South Africa, ISCED and INBAC in Angola, have considerable and comparable collections, but the data regarding these collections is not available on online databases.

The dataset of this collection is available at GBIF (Ceríaco and Marques 2018c; https://www.gbif.org/dataset/ae52efd5-bfd4-4a81-ba12-813cace064f3).

### Mozambique collection

Mozambique has a high diversity of amphibians and reptiles but remains relatively unexplored when compared with other southern African regions (Pietersen 2014). Since Wilhelm Peters’ (1815–1883) historical survey and the publication of his two major works (Peters 1854, 1882), very few surveys have been conducted in the country. The scientific literature of Mozambican herpetofauna was mostly incorporated on comprehensive works about southern and eastern African herpetofauna (e.g., FitzSimons 1962; Broadley 1990a; Channing 2001; Du Preez and Carruthers 2009; Spawls et al. 2018). In the last decade Mozambique has been targeted by researchers and new fieldwork is being conducted. This has already resulted in important contributions and new species descriptions (e.g., Branch et al. 2005a, 2014, 2017; Branch and Bayliss 2009; Branch and Tolley 2010; Portik et al. 2013a; Conradie et al. 2016) and checklists (Broadley 1990b, 1992; Branch et al. 2005b; Schneider et al. 2005; Downs and Wirminghaus 2010; Jacobsen et al. 2010; Pietersen et al. 2013; Ohler and Frétey 2014; Pietersen 2014; Weinell et al. 2017). Currently, the Mozambican herpetofauna comprises more than 100 amphibians and 294 reptiles (Frost 2021; Bates 2018).

The collection comprises 1181 specimens (532 amphibians and 649 reptiles), 26 species of amphibians, and 70 species of reptiles and two subspecies (Table 9), corresponding to approximately 26% of the known taxa for the country. The amphibian collection comprises representatives of 11 families and 16 genera, all anurans. The families Bufonidae, Pyxicephalidae, and Ptychadenidae are the best represented with 193, 125, and 85 specimens, respectively. The reptile collection comprises representatives of 16 families and 50 genera, all squamates, with the exception of four juvenile specimens of *Crocodylus niloticus* Laurenti, 1768. The families Scincidae, Agamidae, and Chamaeleonidae are the best represented in the collection with representatives of all taxa known from the country with 199, 127, and 83 specimens, respectively. Lamprophiidae is the family with the greatest diversity of species (13), followed by the Scincidae (10) and Colubridae (9).

**Table 9.** Overview of the Mozambique amphibian and reptile collections of IICT.

Family	Genus	Species	Localities – Accession number	References	Number of specimens
			(* denotes a type specimen)		
**AMPHIBIANS**					
**ANURA Duméril, 1806**					
**Pipidae** Gray, 1825	*Xenopus* Wagler, 1827	*Xenopus muelleri* (Peters, 1844)	Porto Henrique – IICT/A 1–9/1948, 10–19/1948; Chizizira – IICT/A 1/1965; Gorongosa – IICT/A 44/1955, 49–54/1955, 69–76/1955, 85–90/1955; Caniçado, B.T.L. vivarium – IICT/A 42–42/1963, 278–283/1955; Mozambique [undetermined locality] – IICT/A 38–39/1966	Manaças (1950); Ruas (2002b)	50
**Bufonidae** (Gray, 1825)	*Schismaderma* Smith, 1849	*Schismaderma carens* (Smith, 1848)	Guenguela stream, Chibuto – IICT/A 185–189/1948, 209/1948; Matola – IICT/A 252–255/1948; Moamba – IICT/A 171–179/1948	Manaças (1950), Ruas (2002b)	19
	*Sclerophrys* Tschudi, 1838	*Sclerophrys garmani* (Meek, 1897)	Boane – IICT/A 10/1963, 21/1963, 35/1948, 38/1948, 59/1948, 61/1948, 67/1948, 69/1948; Caniçado, watering channel – IICT/A 24–32/1963, 89/1963, 91/1963, 103/1963, 106/1963; Caniçado – IICT/A 60/1963; Centro Social – IICT/A 114/1963; Guenguela stream, Chibuto – IICT/A 190–215/1948; Guijá – IICT/A 81–83/1963; Manhiça – IICT/A 257/1948; Maqueze – IICT/A 239–245/1948; Moamba – IICT/A 180/1948; Mucomaze – IICT/A 22–33/1948; Porto Henrique – IICT/A 39/1948, 286/1948; Lagoa de S. Matinho – IICT/A 315–318/1955 Mozambique [undetermined locality] – IICT/A Moz3	Manaças (1950); Ruas (2002b)	81
		*Sclerophrys gutturalis* (Power, 1927)	Centro Social – IICT/A 5/1964; Chizizira – IICT/A 18–20/1966; Dondo – IICT/A 163–166/1948; Gorongosa – IICT/A 7–19/1955, 78/1955, 82–83/1955, 95–96/1955, 118–123/1955, 144–147/1955, 152–156/1955, 171–175/1955; Guijá – IICT/A 80/1963; Mauele – IICT/A 79–102/1948; Sussundenga – IICT/A 30–31/1966, 36/1966; Mozambique [undetermined locality] – IICT/A 67A/1948, 309–315/1955, Moz4	Manaças (1950); Ruas (2002b)	83
		*Sclerophrys pusilla* (Mertens, 1937)	Chizizira – IICT/A 33–34/1966; Gorongosa – IICT/A 97–100/1955, 157/1955	Ruas (2002b)	7
		*Sclerophrys* sp.	Licungo river – IICT/A Moz1; Mozambique [undetermined locality] – IICT/A 308/1955		3
**Microhylidae** Günther, 1858 (1843)	*Phrynomantis* Peters, 1867	*Phrynomantis bifasciatus* (Smith, 1847)	Lagoa Chicungue, Bilene – IICT/A 320/1955	Ruas (2002b)	1
**Brevicipitidae** Bonaparte, 1850	*Breviceps* Merrem, 1820	*Breviceps mossambicus* Peters, 1854	Manhiça – IICT/A 68/1948; Ponta do Ouro – IICT/A 34/1948	Manaças (1950); Ruas (2002b)	2
**Hemisotidae** Cope, 1867	*Hemisus* Günther, 1859	*Hemisus marmoratus* (Peters, 1854)	Mozambique [undetermined locality] – IICT/A 258/1948	Ruas (2002b)	1
**Hyperoliidae** Laurent, 1943	*Hyperolius* Rapp, 1842	*Hyperolius marmoratus* Rapp, 1842	Chimoio – IICT/A 74–75/1948; Manhiça – IICT/A 249–250/ 1948, 54–57/1948, 72/1948; Gorongosa – IICT/A 20/1955; Mozambique [undetermined locality] – IICT/A 298/1955, Moz2	Manaças (1950)	12
		*Hyperolius* sp.	Maluana – IICT/A 69/1963; Gorongosa – IICT/A 45/1955		2
		*Hyperolius tuberilinguis* Smith, 1849	Boane – IICT/A 22/1963; Chimoio – IICT/A 25–29/1966; Ulongué – IICT/A 162/1948; Gorongosa – IICT/A 48/1955		8
**Ptychadenidae** Dubois, 1987	*Ptychadena* Boulenger, 1917	*Ptychadena guibei* Laurent, 1954	Gorongosa – IICT/A 55/1955		1
		*Ptychadena mascareniensis* (Duméril & Bibron, 1841)	Manhiça – IICT/A 62–63/1948; Magaiza, Panda – IICT/A 246/1948; Mauele – IICT/A 103–140/1948, 142–161/1948; Pafuri – IICT/A 77/1948; Mozambique [undetermined locality] – IICT/A 289/1955	Manaças (1950); Ruas (2002b)	63
**Ptychadenidae** Dubois, 1987	*Ptychadena* Boulenger, 1917	*Ptychadena oxyrhynchus* (Smith, 1849)	Gorongosa – IICT/A 56–68/1955, 133–137/1955, 161/1955	Ruas (2002b)	19
		*Ptychadena taenioscelis* Laurent, 1954	Mucomaze – IICT/A 21/1948; Gorongosa – IICT/A 295/1955	Ruas (2002b)	2
**Phrynobatrachidae** Laurent, 1941	*Phrynobatrachus* Günther, 1862	*Phrynobatrachus mababiensis* FitzSimons, 1932	Pafuri – IICT/A 76/1948, 78/1948; Umbeluzi – IICT/A 297/1955	Ruas (2002b)	3
		*Phrynobatrachus natalensis* (Smith, 1849)	Namaacha – IICT/A 41/1948, 43/1948, 45/1948, 49–52/1948; Luá (Ile) river – IICT/A 1–2/1955	Ruas (2002b)	12
**Pyxicephalidae** Bonaparte, 1850	*Amietia* Dubois, 1987	*Amietia angolensis* (Bocage, 1866)	Gorongosa – IICT/A 3–6/1955, 21–41/1955, 47/1955, 91–94/1955, 101–131/1955, 138–143/1955, 148–151/1955, 158–160/1955, 167–170/1955, 272–275/1955, 284–28871955; Mozambique [undetermined locality] – IICT/A 29A/1955, Moz5	Ruas (2002b)	83
	*Cacosternum* Boulenger, 1887	Cacosternum cf. boettgeri (Boulenger, 1882)	Gorongosa Mount – IICT/A 225/1955	Ruas (2002b)	1
		Cacosternum cf. nanum (Boulenger, 1887)	Gorongosa – IICT/A 42/1955	Ruas (2002b)	1
	*Pyxicephalus* Tschudi, 1838	*Pyxicephalus edulis* Peters, 1854	Caniçado – IICT/A 90/1963, 92–93/1963; Caniçado, watering channel – IICT/A 101–109/1963; Chibuto – IICT/A 181–182/1948; Namaacha – IICT/A 40/1948; Mozambique [undetermined locality] – 286A/1955	Ruas (2002b)	14
	*Strongylopus* Tschudi, 1838	*Strongylopus rhodesianus* (Hewitt, 1933)	Gorongosa Mount – IICT/A 176–197/1955	Ruas (2002b)	22
	*Tomopterna* Duméril & Bibron, 1841	*Tomopterna marmorata* Peters, 1854	Manhiça – IICT/A 60/1948, 256/1948	Ruas (2002b)	2
**Ranidae** Batsch, 1796	*Amnirana* Dubois, 199	*Amnirana darlingi* (Boulenger, 1902)	Umbeluzi – IICT/A 291–294/1955	Ruas (2002b)	4
**Rhacophoridae** Hoffman, 1932 [1858]	*Chiromantis* Peters, 1854	*Chiromantis xerampelina* Peters, 1854	Chicualacuala – IICT/A 73/1948; Enchisa – IICT/A 20/1948; Manhiça – IICT/A 251/1948; Maqueze – IICT/A 216–232/1948, 234–238/1948; Limpopo river margins – IICT/A 61–66/1963, 74/1963; Moamba – IICT/A 168–170/1948	Manaças (1950); Ruas (2002b)	35
**TOTAL NUMBER OF AMPHIBIAN SPECIMENS**					**532**
**REPTILES**					
**CROCODYLIA** Gmelin, 1789					
**Crocodylidae** Cuvier, 1808	*Crocodylus* Laurenti, 1768	*Crocodylus niloticus* Laurenti, 1768	Nhaluiro – IICT/R 13–16/1971		4
**SQUAMATA** Oppel, 1811					
**Gekkonidae** Gray, 1825	*Afroedura* Loveridge, 1944	*Afroedura loveridgei* Broadley, 1963	Viola, Mazowe River Bridge – IICT/R/DB UM 3992	Broadley (1963)	1
	*Chondrodactylus* Peters, 1870	*Chondrodactylus turneri* (Gray, 1864)	Guenguela stream, Chibuto – IICT/R 1345/1948; Enchisa – IICT/R 178/1948; Magaiza, Panda – IICT/R 1638–1640/1948	Manaças (1952)	5
	*Hemidactylus* Goldfuss, 1820	*Hemidactylus mabouia* (Moreau de Jonnès, 1818)	Chibuto – IICT/R 1320–1322/1948; Mauele – IICT/R 1582/1952; Nhaluiro – IICT/R 8–10/1971; Gorongosa – IICT/R 114/1955, 148–149/1955	Manaças (1952)	10
	*Homopholis* Boulenger, 1885	*Homopholis wahlbergii* (Smith, 1849)	Homoíne – IICT/R 111/1963		1
	*Lygodactylus* Gray, 1864	*Lygodactylus capensis* (Smith, 1849)	Gorongosa – IICT/R 7–8/1955, 14/1955, 16/1955, 115/1955, 146–149/1955, 153A/1955; Lifidzi, Angónia – IICT/R 132A/1948, 1782–1783/1948; Manhiça – IICT/R 644–646/1948;	Manaças (1952, 1961)	16
**Amphisbaenidae** Gray, 1825	*Monopeltis* Smith, 1848	*Monopeltis sphenorhynchus* (Peters, 1879)	Manhiça – IICT/R 532–538/1948	Manaças (1954)	8
	*Zygaspis* Cope, 1885	*Zygaspis violacea* (Peters, 1854)	Manhiça – IICT/R 524–531/1948, 1747/1948	Manaças (1954)	9
**Varanidae** Hardwicke & Gray, 1824	*Varanus* Merrem, 1820	*Varanus exanthematicus* (Bosc, 1792)	Caniçado – IICT/R 39-1963		1
**Lacertidae** Bonaparte, 1831	*Heliobolus* Fitzinger, 1843	*Heliobolus lugubris* (Smith, 1938)	Pafuri – IICT/R 727/1948	Manaças (1961)	1
	*Nucras* Gray, 1838	Nucras cf. holubi (Steindachner, 1882)	Boane – IICT/R 52/1963; Gorongosa – IICT/R 36/1955, 131/1955, 137–138/1955; Lifidzi, Angónia – IICT/R 1792–1795/1948; Namaacha – IICT/R 400/1948	Manaças (1952, 1961	10
**Scincidae** Cuvier, 1808	*Acontias* Cuvier, 1816 [1817]	*Acontias aurantiacus* (Peters, 1854)	Mauele – IICT/R 1023/1948, 1151–1152/1948, 1196/1948, 1199/1948	Manaças (1954)	5
	*Mochlus* Günther, 1864	*Mochlus sundevalli* (Smith, 1849)	Mauele – IICT/R 1164–1167/1948, 1581/1948; Gorongosa – IICT/R 28/1955	Manaças (1952, 1961)	6
	*Panaspis* Cope, 1868	*Panaspis wahlbergii* (Smith, 1849)	Lifidzi, Angónia – IICT/R 1790–1791/1948	Manaças (1952)	2
	*Scelotes* Fitzinger, 1826	*Scelotes arenicola* (Peters, 1854)	Mauele – IICT/R 1153–1156/1948, 1200–1202/1948	Manaças (1954)	7
	*Trachylepis* Fitzinger, 1843	*Trachylepis boulengeri* (Sternfeld, 1911)	Gorongosa – IICT/R 116/1955		1
		*Trachylepis depressa* (Peters, 1854)	Mauele – IICT/R 1584/1948	Manaças (1952)	1
		*Trachylepis margaritifera* (Peters, 1854)	Nhandare river margins, Gorongosa – IICT/R 10–13/1955, 13A/1955; Nhandare river, Gorongosa – IICT/R 30–31/1955; Namaacha – IICT/R 348/1948, 414–415/1948, 441–449/1948; Nhaluiro – IICT/R 4/1971, 7/1971; Sussundenga – IICT/R 10–11/1955	Manaças (1952, 1961)	23
		*Trachylepis* sp.	Mauele – IICT/R 1582/1948; Porto Henrique – IICT/R 235/1948		2
		*Trachylepis striata* (Peters, 1844)	Boane – IICT/R 14/1963, 16/1963, 45/1963, 51/1963, 53–54/1963; Caniçado, watering channel – IICT/R 59/1963; Chibuto, Ribeira de Guenguela – IICT/R 1354–1357/1948; Dondo – IICT/R 911/1948; Lifidzi, Angónia – IICT/R 132C/1948; Limpopo river Dam – IICT/R 33–34/1963; Manhiça – IICT/R 561–613/1948, 1692–1694/1948, 1719/1948, 1744/1948, 1962–1964/1948; Maqueze, Alto Changane – IICT/R 1504–1515/1948; Mauele – IICT/R 1024–1032/1948, 1160–1162/1948, 1184–1192/1948; Moamba – IICT/R 1297–1298/1948, 1323/1948; Mutuali – IICT/R Moz10; Nhaluiro – IICT/R 1–3/1971, 5–6/1971; São Martinho do Bilene – IICT/R 178/1955; road between Inhassoro and Vilanculos – IICT/R 2/1955; Sussundenga – IICT/R 17/1964; Umbeluzi – IICT/R 1–6/1963; Gorongosa – IICT/R 32–33/1955, 87–92/1955	Manaças (1952, 1961)	135
		*Trachylepis varia* (Peters, 1867)	Enchisa – IICT/R 179/1948; Gorongosa Mount – IICT/R 144/1955; Gorongosa – IICT/R 34–35/1955, 117/1955, 144/1955; Lifidzi, Angónia – IICT/R 1789/1948, 1789A/1948; Mauele – IICT/R 1163/1948, 1588/1948; Namaacha – IICT/R 417/1948	Manaças (1952, 1961)	17
**Cordylidae** Mertens, 1937	*Smaug* Stanley, Bauer, Jackman, Branch & Mouton (2011)	*Smaug mossambicus* (FitzSimons, 1958)	Gorongosa – IICT/R 29/1955		1
**Cordylidae** Mertens, 1937	*Platysaurus* Smith, 1844	*Platysaurus intermedius wilhelmi* Hewitt, 1909	Namaacha – IICT/R 418–420/1948, 450–454/1948; 15 mls SE of Manica – IICT/R/DB UM 3579	Manaças (1952)	9
		*Platysaurus maculatus lineicauda* Broadley, 1965	14 mls west of Morrumbala – IICT/R/DB UM 7981	Broadley (1965)	1
		*Platysaurus maculatus maculatus* Broadley, 1965	Mitucué Mountains – IICT/R/DB UM 8064, 8071	Broadley (1965)	2
**Gerrhosauridae** Fitzinger, 1843	*Gerrhosaurus* Wiegmann, 1828	*Gerrhosaurus flavigularis* Wiegmann, 1828	Gorongosa – IICT/R 59–60/1955; 124/1955, 127/1955, 156/1955; Manhiça – IICT/R 1691/1948; Mutuali – IICT/R 1/1953; Mozambique [undetermined locality] – IICT/R Moz12	Manaças (1952, 1961)	8
	*Matobosaurus* Bates & Tolley, 2013	*Matobosaurus validus* (Smith, 1849)	Centro Social – IICT/R 6/1965; Gorongosa – IICT/R 39/1955, 121/1955; Nhandare river margins, Gorongosa – IICT/R 9/1955; Namaacha – IICT/R 416/1948	Manaças (1961)	5
	*Tetradactylus* Merrem, 1820	Tetradactylus cf. ellenbergeri (Angel, 1922)	Meponda – IICT/R Moz6		2
**Agamidae** Gray, 1827	*Acanthocercus* Fitzinger, 1843	*Acanthocercus atricollis* (Smith, 1849)	Boane – IICT/R 17/1963; Caniçado, B.T.L. vivarium – IICT/R 69–70/1963, Moz16; Guenguela stream, Chibuto – IICT/R 1326–1344/1948, 1429–1436/1948; Guijá – IICT/R 964–966/1948; Inharrime – IICT/R 110/1963; Manhiça – IICT/R 553–560/1948; Maqueze, Alto Changane – IICT/R 1499–1500/1948; Limpopo river margins – IICT/R 79/1963; Namaacha – IICT/R 349–413/1948; Sussundenga – IICT/R 39/1966	Manaças (1952)	57
	*Agama* Daudin, 1802	*Agama armata* Peters, 1855	Lifidzi, Angónia – IICT/R 132B/1948, 1784–1788/1948; Manhiça – IICT/R 1695/1948; Mauele – IICT/R 1204–1212/1948; Sussundenga – IICT/R 16–22/1966	Manaças (1952)	20
		*Agama mossambica* Peters, 1854	Centro Social – IICT/R 7/1964; Dondo – IICT/R 898–904/1948; Gorongosa – IICT/R 5–6/1955, 18–26/1955, 61/1955, 64–70/1955, 93–97/1955, 111–113/1955, 128–129/1955, 132–134/1955, 139–141/1955	Manaças (1961)	41
		*Agama kirkii* Boulenger, 1885	Centro Social – IICT/R 8–9/1964; Sussundenga IICT/R 2–4/1964, 12–15/1966		9
**Chamaeleonidae** Gray, 1825	*Chamaeleo* Laurenti, 1768	*Chamaeleo dilepis* Leach, 1819	Boane – IICT/R 12–13/1963, 15/1963; Caniçado IICT/R 67–68/1963, 84–85/1963, 95–97/1963; Gorongosa – IICT/R 471955, 27/1955, 71–80/1955, 81A/1955, 82/1955, 98–99/1955, 100–102/1955, 108–110/1955, 122/1955, 125–126/1955, 161–165/1955, 166–167/1955; Guenguela stream, Chibuto – IICT/R 1346–1353/1948, 1354A/1948, 1356A/1948, 1426–1428/1948, 1439–1440/1948; Guijá – IICT/R 37–38/1948, 58/1963, 71/1963, 115/1963; Lifidzi, Angónia – IICT/R 1796–1800/1948; Manhiça IICT/R 1801/1948, 614/1948, 616–620/1948, 642/1948; Matola – IICT/R 20/1963; Mocuba – IICT/R 3A/1955; São Martinho do Bilene – IICT/R 174–177/1955	Manaças (1952, 1961)	81
		*Chamaeleo gracilis* Hallowell, 1844	Mozambique [undetermined locality] – IICT/R Moz17		1
	*Rhampholeon* Günther, 1874	*Rhampholeon gorongosae* Broadley, 1971	Gorongosa – IICT/R 145/1955		1
SERPENTES					
**Typhlopidae** Merrem, 1820	*Afrotyphlops* Broadley & Wallach, 2009	Afrotyphlops cf. fornasinii (Bianconi, 1849)	Mauele – IICT/R 1157–1159/1948		3
		Afrotyphlops cf. mucruso (Peters, 1854)	Chizizira – IICT/R 38/1966; Nhaluiro – IICT/R 1A/1971; Pafuri – IICT/R Moz26		3
		Afrotyphlops cf. schlegelii (Bianconi, 1849)	Meponda – IICT/R Moz5; Nova Mambone – IICT/R Moz3; presumably Mozambique [undetermined locality] – IICT/R Moz25		3
	*Leptotyphlops* Fitzinger, 1843	*Leptotyphlops incongnitus* (Broadley & Watson, 1976)	Cafumpe – IICT/R 63/1948	Manaças (1954)	1
**Viperidae** Oppel, 1811	*Bitis* Gray, 1842	*Bitis arietans* (Merrem, 1820)	Gorongosa – IICT/R 17/1955, 40A/1955, 43–44/1955, 150/1955; Govuro IICT/R 5/1956; Manhiça IICT/R 797/1948; Tchizigine – IICT/R 10/1964; Ulongué – IICT/R 103/1948; Mozambique [undetermined locality] – IICT/R Moz7, Moz8	Manaças (1959)	11
	*Causus* Wagler, 1830	*Causus defilippii* (Jan, 1863)	Chimoio – IICT/R 35/1966; Gorongosa – IICT/R 49/1955, 82–83/1955	Manaças (1959)	4
		Causus cf. rhombeatus (Lichtenstein, 1823)	Metengo Balama – IICT/R 2/1949	Manaças (1959)	1
**Lamprophiidae** Fitzinger, 1843	*Amblyodipsas* Peters, 1857	*Amblyodipsas microphthalma* (Bianconi, 1852)	Mauele – IICT/R 948/1948, 1150/1948	Manaças (1959)	2
		*Amblyodipsas polylepis* (Bocage, 1873)	Nova Mambone – IICT/R 2/1956		1
	*Aparallactus* Smith, 1849	*Aparallactus capensis* Smith, 1849	Mauele – IICT/R 1012/1948, 1194/1948; Sussundenga – IICT/R 16/1964; Porto Henrique – IICT/R 1642/1948		4
		*Aparallactus lunulatus* (Peters, 1854)	Gorongosa – IICT/R 54/1955	Manaças (1959)	1
	*Atractaspis* Smith, 1849	*Atractaspis bibronii* Smith, 1849	Gorongosa – IICT/R 57/1955		1
	*Boaedon* Duméril, Bibron & Duméril, 1854	*Boaedon capensis* Duméril, Bibron & Duméril, 1854	Boane – IICT/R 8–9/1963; Manhiça – IICT/R 1682/1948, 1684/1948, 1687/1948; Mauele – IICT/R 1248/1948; Namaacha – IICT/R 460/1948; Gorongosa – IICT/R 62/1955, 106/1955, 143/1955; Ulongué – IICT/R 1/1947; Mozambique [undetermined locality] – IICT/R 40/1967	Manaças (1959)	12
		*Boaedon* sp.	Pafuri – IICT/R Moz11		1
	*Limaformosa* Broadley, Tolley, Conradie, Wishart, Trape, Burger, Kusamba, Zassi-Boulou & Greenbaum, 2018	*Limaformosa capensis* (Smith, 1847)	Chibuto – IICT/R 1425/1948	Manaças (1959)	1
	*Lycophidion* Fitzinger, 1843	*Lycophidion capense* (Smith, 1831)	Gorongosa – IICT/R 56/1955	Manaças (1959)	1
	*Psammophis* Boie, 1825	*Psammophis mossambicus* Peters, 1882	Boane – IICT/R Moz1; Gorongosa – IICT/R 47/1955; Manhiça – IICT/R 1685–1686/1948; Maputo – IICT/R 241/1948; Mauele – IICT/R 157/1948, 1015/1948; Nova Mambone – IICT/R 7/1955; São Martinho do Bilene – IICT/R Moz 14–15; Mozambique [undetermined locality] – IICT/R Moz9, 21–23	Manaças (1959)	14
**Lamprophiidae** Fitzinger, 1843	*Psammophis* Boie, 1825	*Psammophis orientalis* Broadley, 1977	Inhassoro – IICT/R 3/1955; Gorongosa – IICT/R 48/1963, 84–86/1955, 160/1955; Nhaluiro – IICT/R 2/1971; Nova Mambone – IICT/R 12/1956	Manaças (1959)	9
	*Psammophylax* Fitzinger, 1843	*Psammophylax tritaeniatus* (Günther, 1868)	Metengo Balama – IICT/R 3/1949; Mozambique [undetermined locality] – IICT/R 252	Manaças (1959)	2
	*Xenocalamus* Günther, 1868	*Xenocalamus bicolor lineatus* Roux, 1907	Manhiça – IICT/R 1589/1948, 1688/1948	Manaças (1952, 1959)	2
**Elapidae** Boie, 1827	*Aspidelaps* Fitzinger, 1843	*Aspidelaps scutatus* Smith, 1849	Mauele – IICT/R 1174/1948	Manaças (1961)	3
	*Dendroaspis* Schlegel, 1848	*Dendroaspis angusticeps* (Smith, 1849)	São Martinho do Bilene – IICT/R 70A/1955		1
		*Dendroaspis polylepis* Günther, 1864	Maputo – IICT/R 173/1955; Mozambique [undetermined locality] – IICT/R 88/1948		2
	*Elapsoidea* Bocage, 1866	*Elapsoidea boulengeri* Boettger, 1895	Guijá – IICT/R 969-1948; Mauele – IICT/R 1193/1948, 1218/1948		2
		*Elapsoidea sundevallii* Smith, 1848	Mauele – IICT/R 1218/1948		1
	*Naja* Laurenti, 1768	*Naja annulifera* Peters, 1854	Moamba – IICT/R 1278/1948; Mozambique [undetermined locality] – IICT/R Moz9		2
		*Naja mossambica* Peters, 1854	Chinde – IICT/R 4/1949; Gorongosa – IICT/R 38/1955	Manaças (1959)	2
**Colubridae** Oppel, 1811	*Crotaphopeltis* Fitzinger, 1843	*Crotaphoepltis hotamboeia* (Laurenti, 1768)	Mauele – IICT/R 1276/1948; Nova Mambone – IICT/R 3/1956; Porto Henrique – IICT/R 1641/1948; São Martinho do Bilene – IICT/R 170/1955; Gorongosa – IICT/R 46/1955, 53/1955, 55/1955, 81/1955, 105/1955, 119/1955; Mozambique [undetermined locality] – IICT/R Moz18	Manaças (1959)	11
	*Dasypeltis* Wagler, 1830	*Dasypeltis scabra* (Linnaeus, 1758)	Maqueze, Alto Changane – IICT/R 1501/1948	Manaças (1959)	1
	*Dipsadoboa* Günther, 1858	*Dipsadoboa aulica* (Günther, 1864)	Chibuto – IICT/R 1324/1948; Nova Mambone – IICT/R 1A/1956	Manaças (1959)	2
	*Dispholidus* Duvernoy, 1832	*Dispholidus typus* (Smith, 1828)	Chibuto – IICT/R 1422–1423/1948; Gorongosa – IICT/R 45/1955, 107/1955, 118/1955, 136/1955; Guenguela stream, Chibuto – IICT/R 1424/1948; Manhiça – IICT/R 1683/1948; Nova Mambone – IICT/R 2A/1948; São Martinho do Bilene – IICT/R 171/1955;	Manaças (1959)	10
	*Philothamnus* Smith, 1840	*Philothamnus angolensis* Bocage, 1882	Angónia – IICT/R 5/1949; Gorongosa – IICT/R 45A/1955, 58/1955, 130/1955, 154/1955, 157–158/1955; Mozambique [undetermined locality] – IICT/R Moz20		8
		*Philothamnus hoplogaster* (Günther, 1863)	Chizizira – IICT/R 15/1964; Gorongosa – IICT/R 123/1956, 153/1956, 155/1956, 159/1956; Nova Mambone – IICT/R 1/1956	Manaças (1959)	6
		*Philothamnus semivariegatus* (Smith, 1840)	Chizizira – IICT/R 11/1964; Gorongosa – IICT/R 103/1955; Meponda, Niassa lake – IICT/R Moz2; Meponda – IICT/R Moz4; Nova Mambone – IICT/R 4/1956; Sussundenga – IICT/R 14/1964; Mozambique [undetermined locality] – IICT/R A/1948, Moz24	Manaças (1959)	8
	*Telescopus* Wagler, 1830	*Telescopus semiannulatus* Smith, 1849	Nova Mambone – IICT/R 8/1956; Pafuri – IICT/R 725/1948		2
	*Thelotornis* Smith, 1849	*Thelotornis capensis* Smith, 1849	Gorongosa – IICT/R 50–51/1955, 130A/1955, 151–152/1955		5
**Natricidae** Bomaparte, 1838	*Natriciteres* Loveridge, 1953	*Natriciteres olivacea* (Peters, 1854)	Gorongosa – IICT/R 52/1955, 120/1955; Tchizine – IICT/R 12/1964	Manaças (1959)	3
**TOTAL NUMBER OF REPTILES SPECIMENS**					**649**

**Figure 13.**
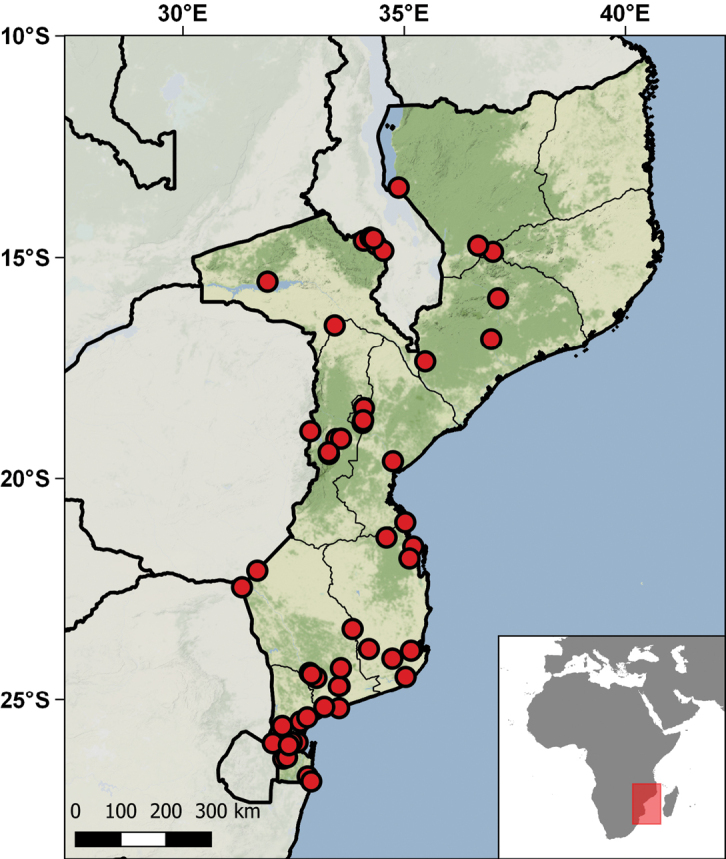
Distribution of the Mozambique localities represented in the IICT herpetological collections.

Geographically, these specimens are from 62 different localities distributed in the nine provinces of the country (Table 10; Fig. 13). The temporal range of collecting events is from 1947 to 1971, although the majority of the specimens were collected in 1948 and 1955. The main contributors to the collections were Fernando Frade and his team during the Scientific Mission to Mozambique, and Marques da Silva (dates of birth and death unknown) from the Trypanosomiasis Eradication Mission. Several specimens were also collected by staff of the Zootechnical Post in Angonia.

**Table 10.** Gazetteer of Mozambique localities of IICT specimens. Latitude and longitude decimal coordinates are presented in WGS-84 projection.

Province	Verbatim locality	Current locality	Latitude and Longitude	Uncertainty (meters)	Elevation (meters)	Number of taxa/records
Gaza	Alferes Chamusca, canal de rega (Guijá) [or Viveiro de B.T.L., Guijá, Alferes Chamusca]	Caniçado [or Caniçado, watering channel; or Caniçado, B.T.L. vivarium]	-24.49750, 33.01333	9059	34	7/38
Gaza	Barragem do Rio Limpopo	Limpopo river Dam	-24.384942, 32.862182	3182	36	1/2
Gaza	Chibuto	Chibuto	-24.689738, 33.538492	3660	104	5/9
Gaza	Chicuala-Cuala	Chicualacuala [a.k.a Eduardo Mondlane]	-22.079167, 31.678611	3036	456	1/1
Gaza	Guijá	Guijá	-24.418921, 32.901765	11027	36	5/13
Gaza	Ribeira de Guenguela, Chibuto	Guenguela stream, Chibuto	-24.69473, 33.520468	3113	22	7/79
Gaza	Maquese	Maqueze	-24.279167, 33.566667	3036	23	2/29
Gaza	Maquese (Alto Changane)	Maqueze, Alto Changane	-23.394722, 33.831389	3996	57	3/15
Gaza	Margens do Rio Limpopo	Limpopo river margins	-25.19361, 33.52583	3036	8	2/8
Gaza	Pafuri [or Mabaça (Pafuri)]	Pafuri	-22.453056, 31.332778	500	223	6/7
Inhambane	Govuro	Govuro	-21.33047, 34.59716	3036	127	1/1
Inhambane	Homoíne	Homoíne	-23.884444, 35.151389	3036	108	1/1
Inhambane	Inharime	Inharrime	-24.476944, 35.030278	3036	39	1/1
Inhambane	Inhassoro	Inhassoro	-21.534722, 35.202222	3036	42	1/1
Inhambane	Magaiza (Panda)	Magaiza, Panda	-23.847222, 34.199444	2639	108	2/4
Inhambane	Mauele	Mauele [undetermined locality]	–	–	–	20/149
Inhambane	Mambone	Nova Mambone	-20.988056, 35.022222	500	5	10/10
Inhambane	Panda	Panda	-24.063333, 34.730278	7220	160	1/1
Inhambane	Inhassoro, Vilanculos	road between Inhassoro and Vilanculos	-21.803504, 35.117303	27302	23	1/1
Inhambane	S. Martinho de Bilene	São Martinho do Bilene / Lagoa São Martinho / Lagoa Chicungue, Bilene	-25.281111, 33.253889	10000	21	8/16
Manica	15 mls SE of Vila Manica, P.E.A.	15 mls SE of Vila Manica, P.E.A.	-18.933332, 32.877127	1000	700	1/1
Manica	Chimoio [or Vila Perry]	Bengo, Chimoio	-19.110117, 33.462887	4772	715	3/8
Manica	Cafumpe	Cafumpe	-19.101389, 33.570278	765	715	1/1
Manica	Chizizira [or Posto Piscicola Chizizira]	Chizizira	-19.448611, 33.297222	3433	536	6/9
Manica	Sussundenga	Sussundenga	-19.403889, 33.290278	3036	600	8/20
Maputo	Boame [or Boane]	Boane	-26.041667, 32.325278	3036	51	8/24
Maputo	Centro Social	Centro Social [undetermined locality]	–	–	–	5/6
Maputo	Echisa (Maputo)	Alto Enchisa	-26.329907, 32.264797	4298	47	3/3
Maputo	Maluana	Maluana	-25.495, 32.654167	2000	62	1/1
Maputo	Manhiça	Manhiça	-25.402222, 32.807222	6500	22	19/121
Maputo	Lourenço Marques	Maputo	-25.965278, 32.589167	7000	21	2/2
Maputo	Matola	Matola	-25.962222, 32.458889	7317	42	2/6
Maputo	Mohamba [or Mohamba, Chibuto, or Moamba]	Moamba	-25.596111, 32.243333	3036	112	6/18
Maputo	Mucomaze, Maputo	Mucomaze	-26.733333, 32.816667	3036	30	2/13
Maputo	Namaacha	Namaacha	-25.982624, 32.027959	3036	575	9/40
Maputo	Porto Henrique, Maputo [or Porto Henriques]	Porto Henrique	-26.3, 32.348889	1000	32	5/24
Maputo	Ponta do Ouro, Maputo	Ponta do Ouro	-26.842778, 32.896944	1807	34	1/1
Maputo	Umbeluzi	Umbeluzi	-26.028333, 32.39	1977	16	3/11
Nampula	Mutuali	Mutuali	-14.870556, 37.004444	4000	587	2/2
Niassa	Meponda, Lago Niassa	Meponda, Lago Niassa	-13.421667, 34.871667	2071	476	1/1
Niassa	Meponda	Meponda	-13.421667, 34.871667	3036	483	3/3
Niassa	Mitucué Mountains, Niassa	Mitucué Mountains	-14.731545, 36.66941	5311	1136	1/2
Sofala	Dondo	Dondo	-19.609444, 34.743056	1787	55	3/10
Sofala	Margens do Rio Nhandare, Gorongosa [or Vila Paiva de Andrada, Rio Nhantare]	Nhandare river margins, Gorongosa [or Nhandare river, Gorongosa]	-18.74608, 34.05541	35862	205	2/8
Sofala	Serra da Gorongosa	Mount Gorongosa	-18.41098, 34.086773	13178	1487	3/24
Sofala	Vila Paiva de Andrada [or Gorongosa]	Gorongosa	-18.684092, 34.070301	1815	372	40/331
Tete	Angónia	Angónia	-14.715833, 34.373056	40000	1250	1/1
Tete	Fumo- Chide, Angónia	Chidê	-14.619722, 34.085278	460	1147	1/1
Tete	Lifidzi (Angónia)	Lifidzi, Angónia	-14.55, 34.233333	1800	1250	7/23
Tete	Metengo, Balama, Angónia	Metengo Balama	-14.848056, 34.526111	2000	1370	2/2
Tete	Nhaluiro [or Nhaluiro Velho, or Ribeira Nhaluiro]	Nhaluiro	-15.543889, 31.909722	3036	481	6/16
Tete	Vila Coutinho	Ulongué	-14.574444, 34.306111	3036	1295	3/3
Tete	Viola (Mazoe River Bridge), P.E.A.	Viola, Mazowe River Bridge	-16.53173, 33.428165	3036	173	1/1
Zambézia	14 mls west of Morrumbala	14 mls west of Morrumbala	-17.354788, 35.472782	1000	309	1/1
Zambézia	Mocuba	Mocuba	-16.845556, 36.964167	1439	148	1/1
Zambézia	Rio Licungo	Licungo river [undetermined locality]	–	–	–	1/1
Zambézia	Rio Luá (Ile)	Luá (Ile) river	-15.92, 37.119167	6000	415	1/2
	Tchizine	Tchizine [undetermined locality]	–	–	–	1/1
	Tchizigine	Tchizigine [undetermined locality]	–	–	–	1/1
	Moçambique	Mozambique [undetermined locality]	–	–	–	15/30
		Presumably Mozambique	–	–	–	2/2

The collection was primarily studied and published on by Sara Manaças resulting in four publications (Manaças 1950b, 1954, 1959, 1961a), while Clara Ruas published a paper focused on the amphibians (Ruas 2002b). In 1965 the Zimbabwean herpetologist, Donald G. Broadley (1932–2016) described two new species of *Platysaurus*, the Spotted Flat Lizard, *Platysaurus maculatus maculatus* (two paratypes) and the Striped Tail Flat Lizard, *Platysaurus maculatus lineicauda* (one paratype), based on some specimens from this collection (Broadley 1965). The previously unpublished specimens of Wilhelm’s Flat Lizard, *Platysaurus intermedius wilhelmi* Hewitt, 1909, represent the first records for the subspecies in Mozambique.

Globally, this is the largest collection of amphibians and reptiles from Mozambique with 1168 specimens, followed by those of the TM with 847, the MCZ with 643, and the MNHN with 510 (data retrieved from GBIF.org in February 2021). Other specimens exist in several other museums, with numbers ranging from 132 to 456 specimens per collection (data retrieved from GBIF.org in February 2021).

The dataset of this collection is available at GBIF (Ceríaco and Marques 2018d; https://www.gbif.org/dataset/3c66c8f5-a981-46ea-8b0f-6ae44f799220).

### Macau collection

The most comprehensive account of the herpetofauna of China was provided by Zhao and Adler (1993), listing a total of 661 species. Since then, that number almost doubled and new species are being described every year (Murphy 2016; Kai et al. 2020). However, works dedicated to the herpetofauna of Macau are scarce (e.g., Barros 1978), contrary to those concerning other Chinese territories, such as Hong Kong (e.g., Romer 1975, 1979a, b; Karsen et al. 1998). Easton and Leung (1993) provided the only systematic account of the reptiles and amphibians of Macau that is available in English. This work lists a total of 31 species for Macau and was mostly based on material collected between 1989 and 1992 that was deposited in the BPBM. Zhao and Leung (1999) published an updated checklist (available only in Chinese) of the amphibians and reptiles from Macau and recorded a total of 38 species for the region. The most recently updated checklist of Macau herpetofauna is available on the online database Macau Biodiversity (www.macaubiodiversity.org; accessed 5 February 2021), listing nine species of amphibians and 30 species of reptiles occurring in the territory.

The collection comprises a total of 318 specimens (266 amphibians and 52 reptiles) that include nine species of amphibians and 16 species of reptiles (Table 11). The amphibian collection includes representatives of eight genera and six different families, including two specimens of the iconic South China Giant Salamander, *Andrias sligoi* (Boulenger, 1924). This collection is dominated by the Asian Toad, *Duttaphrynus melanostictus* (Schneider, 1799) with 151 specimens, followed by the Banded Bull Frog, *Kaloula pulchra* Gray, 1831 with 27 specimens, and the Ornamented Pygmy Frog, *Microhyla ornata* (Duméril & Bibron, 1841) with 18 specimens. Specimens of the Asian Toad account for nearly half of all the material from Macau, while most of the remaining amphibian species are represented by 10 specimens or less. The reptile collection is considerably smaller, with only 52 specimens, but more diverse, covering 13 genera from eight different families. The Gekkonidae is the best represented family among squamates, with 18 specimens from genera *Gekko* and *Hemidactylus*, followed by the Agamidae with five specimens of *Calotes versicolor* (Daudin, 1802) and four specimens of *Leiolepis reevesii* (Grey, 1831). Most snake species are represented by a single specimen, and *Colubridae* is the most represented family with seven specimens from four different species.

**Table 11.** Overview of the Macau amphibian and reptile collections of IICT.

Family	Genus	Species	Localities – Accession number	References	Number of specimens
			(* denotes a type specimen)		
**AMPHIBIANS**					
**URODELA Duméril, 1806**					
**Cryptobranchidae** Fitzinger, 1826	*Andrias* Tschudi, 1837	*Andrias sligoi* (Boulenger, 1924)	Bought in a market [undetermined locality] – IICT/A A-B/1988	Dias et al. (1994)	2
**ANURA Duméril, 1806**					
**Bufonidae** (Gray, 1825)	*Duttaphrynus* Frost, Grant, Faivovich, Bain, Haas, Haddad, de Sá, Channing, Wilkinson, Donnellan, Raxworthy, Campbell, Blotto, Moler, Drewes, Nussbaum, Lynch, Green & Wheeler, 2006	*Duttaphrynus melanostictus* (Schneider, 1799)	Cheoc Van – IICT/A 21-22/1989; Hac-Sá barracks road – IICT/A 81-88/1989, 154-161/1989; Old Military Road – IICT/A 89-100/1989; Hac-Sá – 12/1988, 31/1988, 19-20/1089, 37-43/1989, 153/1989, 165/1989, 183-195/1989; former N°1 post of Maritime Delegation – IICT/A 13/1989, 32/1989, 61-62/1989, 65-70/1989, 164/1989; Maritime Delegation road – IICT/A 1-6/1989; On the Military Road to Coloane village – IICT/A 128/1989; Coloane Alto Military Road – IICT/A 140-152/1989; Garden of Montanha Russa [or Montanha Russa Municipal Park] – IICT/A 23-29/1989, 207-208/1989, 210-227/1989, 230-237/1989, 239-246/1989; Ka-Ho – IICT/A 31/1989; Macau [undetermined locality] – IICT/A 248-252/1989; Bought in a market [undetermined locality] – IICT/A 166-169/1989; Mong-Há Municipal Park – IICT/A 59-60/1989, 247/1989; Picnic Park – IICT/A 33-36/1989; Seac-Pai-Van – IICT/A 13/1988, 117-118/1989	Mendes et al. (1994a)	151
**Dicroglossidae** Anderson, 1871	*Hoplobatrachus* Peters, 1863	*Hoplobatrachus rugulosus* (Wiegmann, 1834)	Bought in a local market [undetermined locality] – IICT/A 76-77/1989		2
	*Fejervarya* Bolkay, 1915	*Fejervarya limnocharis* (Gravenhorst, 1829)	Hac-Sá barracks road – IICT/A 163/1989; Old Military Road – IICT/A 101-104/1989; On the road from Maritime Delegation to Cheoc Van – IICT/A 9/1989; On the Military Road to Coloane village – IICT/A 125-127/1989; Garden of Montanha Russa [or Montanha Russa Municipal Park] – IICT/A 124/1989	Mendes et al. (1994b)	10
**Microhylidae** Günther, 1858	*Kaloula* Gray, 1831	*Kaloula pulchra* Gray, 1831	Old Military Road – IICT/A 111-116/1989; Hac-Sá – IICT/A 196-205/1989; On the road from Maritime Delegation to Cheoc Van – IICT/A 7-8/1989; On the Military Road to Coloane village – IICT/A 129-130/1989; Garden of Montanha Russa [or Montanha Russa Municipal Park] – IICT/A 119-123/1989; Macau [undetermined locality] – IICT/A 253/1989; Mong-Há Municipal Park – IICT/A 78/1989	Mendes et al. (1994b)	27
	*Microhyla* Tschudi, 1838	*Microhyla pulchra* (Hallowell, 1861)	Hac-Sá – IICT/A 206/1989	Mendes et al. (1994b)	1
		*Microhyla ornata* (Duméril & Bibron, 1841)	Old Military Road – IICT/A 105-110/1989; former N°1 post of Maritime Delegation – IICT/A 15-18/1989, 71-75/1989; On the road from Maritime Delegation to Cheoc Van – IICT/A 10/1989; Coloane Alto Military Road – IICT/A 139/1989; Seac-Pai-Van – IICT/A 14/1989	Mendes et al. (1994b)	18
		*Microhyla* sp.	On the Military Road to Coloane village – IICT/A 132-138/1989		7
**Ranidae** Batsch, 1796	*Sylvirana* Dubois, 1992	*Sylvirana guentheri* (Boulenger, 1882)	Garden of Montanha Russa [or Montanha Russa Municipal Park] – IICT/A 30/1989	Mendes et al. (1994b)	1
**Rhacophoridae** Hoffman, 1932	*Polypedates* Tschudi, 1838	*Polypedates megacephalus* Hallowell, 1861	On the road from Maritime Delegation to Cheoc Van – IICT/A 11-12/1989; On the Military Road to Coloane village – IICT/A 131/1989; former N°1 post of Maritime Delegation – IICT/A 14/1989	Mendes et al. (1994b)	4
**Rhacophoridae** Hoffman, 1932	*Polypedates* Tschudi, 1838	*Polypedates megacephalus* Hallowell, 1861	Ka-Ho – IICT/A Macau1; former N°1 post of Maritime Delegation – IICT/A Macau2; Macau [undetermined locality] – IICI/A Macau3		43
**TOTAL NUMBER OF AMPHIBIAN SPECIMENS**					**266**
**REPTILES**					
**CHELONIA** Brongniart, 1800					
**Geomydidae** Theobald, 1868	*Mauremys* Ritgen, 1828	*Mauremys reevesii* (Gray, 1831)	Macau [undetermined locality] – IICT/R Macau1-2		3
**SQUAMATA** Oppel, 1811					
**Agamidae** Gray, 1827	*Calotes* Cuvier, 1816	*Calotes veriscolor* (Daudin, 1802)	Hac-Sá – IICT/R 10-11/1988, 28/1988; Ka-Ho – IICT/R 23/1989; Mong-Há Inn – IICT/R 3-1988		5
	*Leiolepis* Cuvier, 1829	*Leiolepis reevesii* (Gray, 1831)	Hac-Sá – IICT/R 13-14/1989, 16-17/1989	Pinheiro (1994)	4
**Gekkonidae** Gray, 1825	*Gekko* Laurenti, 1768	*Gekko chinensis* (Gray, 1842)	Mong-Há Municipal Park – IICT/R 20-21/1989; former N°1 post of Maritime Delegation – IICT/R 7-9/1989		5
	*Hemidactylus* Gray, 1825	*Hemidactylus bowringii* (Gray, 1845)	Seac-Pai-Van – IICT/R 15-17/1988; Hac-Sá – IICT/R 18-19/1989		5
		*Hemidactylus brookii* Gray, 1845	Mong-Há Inn – IICT/R 4-5/1988; Coloane – IICT/R 8/1988		3
		*Hemidactylus garnotii* Duméril & Bibron, 1836	Seac-Pai-Van – IICT/R 21-22/1988		2
		*Hemidactylus* sp.	Mong-Há Inn – IICT/R 22/1989; Coloane – IICT/R 29-30/1988		3
**Scincidae** Cuvier, 1808	*Scincella* Mittleman, 1950	*Scincella reevesii* (Gray, 1838)	Mong-Há Inn – IICT/ 1-2-/1988; Hac-Sá Barracks Road – IICT/R 2/1989; former N°1 post of Maritime Delegation – IICT/R 3-4/1989		5
SERPENTES					
**Colubridae** Oppel, 1811	*Lycodon* Boie, 1827	*Lycodon subcinctus* Boie, 1827	On the Military Road to Coloane village – IICT/R 12/1989		1
	*Ptyas* Fitzinger, 1843	*Ptyas korros* (Schlegel, 1837)	Hac-Sá – IICT/R 6/1989, 15/1989; Bought in a local market – IICT/R 41/1988		3
		*Ptyas mucosa* (Linnaeus, 1758)	Hac-Sá – IICT/R 6/1988		1
	*Xenochrophis* Günther, 1864	*Xenochrophis piscator* (Schneider, 1799)	Maritime Delegation road – IICT/R 10-11/1989		2
**Elapidae** Boie, 1827	*Bungarus* Daudin, 1803	*Bungarus multicinctus* Blyth, 1861	Bought in a market [undetermined locality] – IICT/R 38/1988		1
	*Ophiophagus* Günther, 1864	*Ophiophagus hannah* (Cantor, 1836)	Bought in a market [undetermined locality] – IICT/R 39/1988		1
**Viperidae** Oppel, 1811	*Trimeresurus* Lacepede, 1804	*Trimeresurus albolabris* Gray, 1842	Natural and Agrarian Museum, Seac-Pai-Van [or Museu Natural e Agrário, Seac-Pai-Van]– IICT/R 5/1989		1
**Typhlopidae** Merrem, 1820	*Indotyphlops* Hedges, Marion, Lipp, Marin & Vidal, 2014	*Indotyphlops braminus* (Daudin, 1803)	Hac-Sá – IICT/R 1/1989, 19-20/1988; Seac-Pai-Van – IICT/ R 25/1988		5
			Bought in a market [undetermined locality] – IICT/R 24/1988, 40/1988		2
**TOTAL NUMBER OF REPTILE SPECIMENS**					52

**Figure 14.**
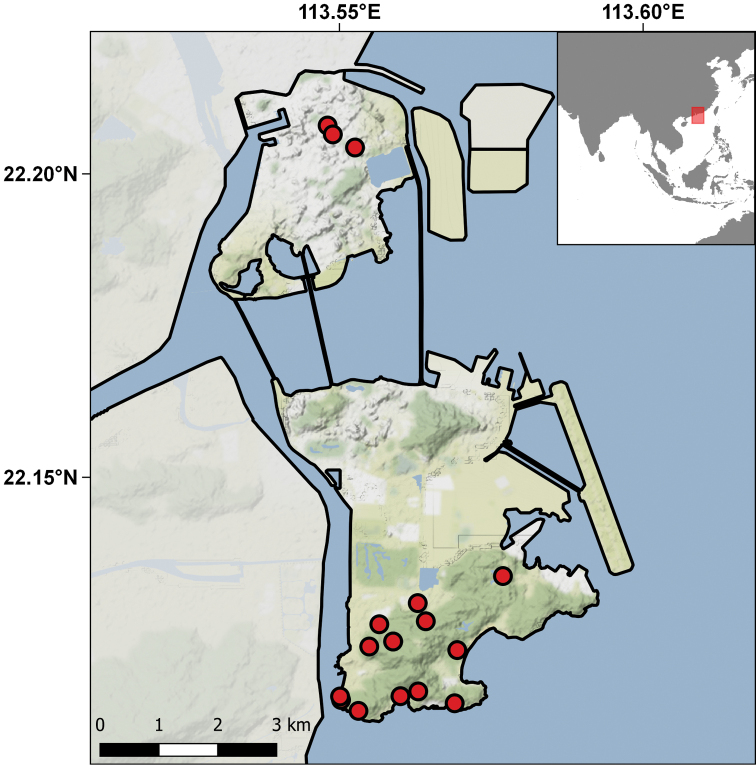
Distribution of the Macau localities represented in the IICT herpetological collections.

Except for one specimen collected in 1984, all the specimens were collected in November 1988 and July 1989 during two expeditions organised and led by Jaime Augusto Travassos Dias (1920–1999; Dias et al. 1994). Geographically, this material comes from 19 different localities in the Macau Peninsula and Coloane Island (Table 12; Fig. 14). Hac-Sá, in Coloane, and Garden of Montanha Russa in Macau are the best represented localities with 52 and 51 records, respectively, while few other localities exceed 20 records. Some material was purchased at local markets and might have been imported from neighbouring regions of China. This is the case for *Andrias sligoi* and *Ophiophagus hannah* (Cantor, 1836), two iconic species that are represented in the IICT collection but were not recorded by Easton and Leung (1993) nor Zhao and Leung (1999).

**Table 12.** Gazetteer of Macau localities of IICT specimens. Latitude and longitude decimal coordinates are presented in WGS-84 projection.

Island	Verbatim locality	Current locality	Latitude and Longitude	Uncertainty (meters)	Elevation (meters)	Number of taxa/records
Coloane	Chok Van	Cheoc Van	22.113993, 113.560058	100	51	1/2
Coloane	Coloane	Coloane	22.126318, 113.56423	400	63	3/5
Coloane	Estrada do Quartel de Hac-Sa [or Estrada do Quartel de Ac-Sa]	Hac-Sá Barracks road	22.112826, 113.568982	108	40	3/18
Coloane	Estrada Militar Velha	Old military road	22.122163, 113.554919	200	57	4/28
Coloane	Granja (Serviços Agrários da Câmara Municipal das Ilhas)	Natural and Agrarian Museum, Seac-Pai-Van [or Museu Natural e Agrário, Seac-Pai-Van]	22.125826, 113.556575	62	8	1/1
Coloane	Hac-Sa [or Ac-Sa]	Hac-Sá	22.121577, 113.569413	786	22	9/52
Coloane	Posto n°1	former N°1 post of Maritime Delegation	22.113377, 113.550274	60	9	5/26
Coloane	Estrada da Delegação [or Estrada da Delegação Marítima]	Maritime Delegation road	22.113943, 113.550113	200	7	2/8
Coloane	Estrada da Delegação Marítima a Chok-Van	On the road from Maritime Delegation to Cheoc Van	22.111574, 113.55314	300	52	4/6
Coloane	Estrada Militar a Coloane	On the Military road to Coloane village	22.122987, 113.558851	496	92	6/15
Coloane	Estrada Militar do Alto de Coloane	Alto de Coloane, military road	22.122987, 113.558851	496	92	2/14
Coloane	Ka-Ho	Ka-Ho	22.133762, 113.576933	26	56	3/3
Coloane	Parque de Merendas	Picnic Park	22.114778, 113.562985	60	85	1/4
Coloane	Siac-Pai-Van	Seac-Pai-Van	22.129308, 113.562903	300	13	5/10
Macau	Montanha Russa [or Montanha Russa (jardim)]	Garden of Montanha Russa [or Montanha Russa Municipal Park]	22.204354, 113.552576	60	22	4/51
Macau	Mong-Há	Mong-Há Municipal Park	22.20797, 113.548063	225	33	3/6
Macau	Pousada Mong-Há	Mong-Há Inn	22.206509, 113.548921	40	28	4/6
–	Comprada num mercado em Macau [or Comprada em Macau, or Mercado]	Bought in a local market [undetermined locality]	–	–	–	6/9
–	Macau	Macau [undetermined locality]	–	–	–	4/8

This collection was partly studied by Dias et al. (1994), Mendes et al. (1994a, b), and Pinheiro (1994), but none of these authors provided a complete overview of the collection.

Even though specimens from mainland China are common in museum collections, specimens of amphibians and reptiles from Macau are scarce in western museum collections. IICT holds the largest known herpetological collection from that region, with 323 specimens, followed by the BPBM with 27 specimens (Easton and Leung 1993).

The dataset of this collection is available at GBIF (Ceríaco and Marques 2018e; https://www.gbif.org/dataset/7df6557f-1996-4874-a08a-1ac5718ef413).

### Portuguese India (Goa) Collection

The Indian herpetofauna is still poorly known and lacks an updated systematic account. Despite recent efforts to summarise the knowledge of Indian amphibians and reptiles in checklists and field guides (e.g., Daniel 2002; Venugopal 2010; Das and Das 2017), the works of Boulenger (1890), Smith (1931, 1935, 1943), and Sharma (1998, 2007) remain the most comprehensive accounts of India’s herpetofauna. There are approximately 610 species of reptiles (Khandekar et al. 2021) and 472 species of amphibians (Gosavi et al. 2021) currently known from India, of which nearly half are endemic. However, these numbers are expected to increase as several new species have been described in recent years (e.g., Biju et al. 2011; Agarwal et al. 2019; Giri et al. 2019). While there are several accounts of the herpetofauna of former British India (e.g., Günther 1864; Boulenger 1890; Smith 1931, 1935, 1943; Constable 1949), reports on the reptiles and amphibians of former Portuguese territories in India are scarce (see Das 2004 for a detailed history of herpetological research in India). The first contribution from Portuguese naturalists was published by Bocage (1863), reporting only seven species. Later, Ferreira (1897a) provided an account of the Indian reptiles and amphibians present in the collections of the Zoological Section of the National Museum of Lisbon, listing 68 species of reptiles and 8 species of amphibians. All of those specimens were destroyed in a fire in 1978. Themido (1941) gave an account of the small collection of reptiles from Portuguese India present in the Museu Zoológico da Universidade de Coimbra (currently MCUC), covering a total of 12 species of reptiles. After the annexation of Goa by the Republic of India in 1961, the ZSI collected 413 specimens of reptiles from 40 localities in the state of Goa between 1966 and 1969. This material was later examined and published by Sharma (1976), who listed 46 species, including two newly described species of lizards (*Cnemaspis goaensis* and *Lygosoma goaensis*).

The collection comprises only 26 specimens (12 amphibians and 14 reptiles) belonging to three different species (Table 13). The Dicroglossidae is the only amphibian family represented in the collection, with 12 specimens of *Hoplobatrachus tigerinus* (Daudin, 1802). Squamates are represented by the families Agamidae, with 13 specimens of *Calotes versicolor*, and Scincidae, with only one specimen of *Lygosoma punctata* (Gmelin, 1799).

**Table 13.** Overview of the former Portuguese India amphibian and reptile collections of IICT.

**Family**	**Genus**	**Species**	**Localities – Accession number**	**References**	**Number of specimens**
			(* denotes a type specimen)		
**AMPHIBIANS**					
**Dicroglossidae** Anderson, 1871	*Hoplobatrachus* Peters, 1863	*Hoplobatrachus tigerinus* (Daudin, 1802)	Santa Cruz [or Calaphur] – IICT/A 15-21/1959; Taleigão – IICT/A 10-14/1959	Manaças (1961)	12
**TOTAL NUMBER OF AMPHIBIAN SPECIMENS**					**12**
**REPTILES**					
**SQUAMATA** Oppel, 1811					
**Agamidae** Gray, 1827	*Calotes* Cuvier, 1816	*Calotes veriscolor* (Daudin, 1802)	Santa Cruz [or Calaphur] – IICT/R 1-9/1959; Pernem – IICT/R India1-4	Manaças (1961)	13
**Scincidae** Cuvier, 1808	*Lygosoma* Hardwicke & Gray, 1827	*Lygosoma punctata* (Gmelin, 1799)	Pernem – IICt/R India5		1
**TOTAL NUMBER OF REPTILE SPECIMENS**					**14**

**Figure 15.**
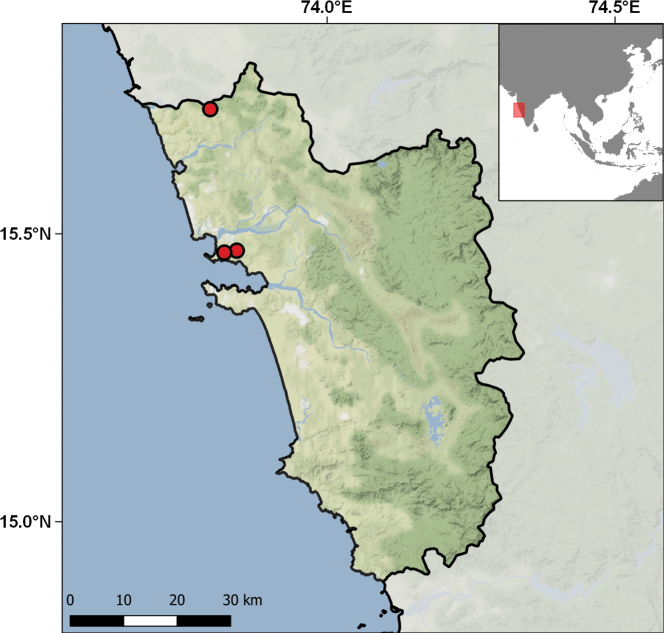
Distribution of the Goa localities represented in the IICT herpetological collections.

Geographically, this collection covers only three localities in the state of Goa: Santa Cruz (or Calaphur) with 16 records, Taleigão, and Pernem, with five records each (Table 14; Fig. 15). This material was opportunistically collected in November 1959 by Armando Castel-Branco (1909–1977), a researcher for the CZL, while conducting entomological studies in Goa. This modest collection was studied and published by Manaças (1961b).

**Table 14.** Gazetteer of the former Portuguese India localities of IICT specimens. Latitude and longitude decimal coordinates are presented in WGS-84 projection.

State	Verbatim locality	Current locality	Latitude and Longitude	Uncertainty (meters)	Elevation (meters)	Number of taxa/records
Goa	Pernem	Pernem	15.71674, 73.796996	1833	23	2/5
Goa	Santa Cruz	Santa Cruz [or Calaphur]	15.470833, 73.843056	1833	12	2/16
Goa	Taleigão	Taleigão	15.4675, 73.821389	1833	16	1/5

The dataset of this collection is available at GBIF (Ceríaco and Marques 2018e; https://www.gbif.org/dataset/7df6557f-1996-4874-a08a-1ac5718ef413).

### East Timor collection

Despite a series of herpetological surveys that started in the beginning of the twenty-first century (Kaiser et al. 2011; O’Shea et al. 2012), the knowledge of the reptiles and amphibians of this country is still very incipient. The most recent account of the herpetofauna of mainland East Timor was provided by O’Shea et al. (2015) and reports at least 60 species of amphibians and reptiles, including more than 20 undescribed species. A first record of the herpetofauna of Antaúro Island was provided by Kaiser et al. (2013), listing 14 species of reptiles, of which at least five are likely undescribed endemic species. The exclave of Oecusse District, in the western part of Timor Island, was also surveyed in recent years (Sanchez et al. 2012). Historically, even though some authors addressed the herpetofauna of Timor (e.g., van Lidth de Jeude 1895), these efforts are mostly focused on the western part of the island, with records from the territory that is now East Timor being scarce. The Portuguese contribution to the knowledge of the herpetofauna of East Timor started with the explorations of Francisco Newton between 1895 and 1897, whose specimens were examined by Ferreira (1897b, 1898) and were subsequently lost in the fire that destroyed the zoological collections of Museu Bocage, Lisbon, in 1978. Other Portuguese contributions were published by Themido (1941) based on material collected by several contributors and offered to the MCUC. Following Portuguese decolonisation in 1975, Indonesia invaded the country, instituting a period of political instability that halted biological research. This period lasted until 2002, when East Timor regained independence, and was followed by a series of surveys that greatly improved previous knowledge of the country’s herpetofauna and are expected to continue increasing the number of known species (Kaiser et al. 2011, 2013; O’Shea et al. 2012, 2015; Sanchez et al. 2012). Most specimens vouchered during these recent surveys have been deposited in the collections of the USNM.

The material from East Timor constitutes the smallest herpetological sub-collection of the IICT, with only 17 reptile specimens. Despite its small size, this collection covers nine species of reptiles from eight different families (Table 15), corresponding to roughly 15% of the total number of species currently known from East Timor. The Gekkonidae is the best represented family, with three specimens of *Hemidactylus frenatus* Duméril & Bibron, 1836 and two specimens of *Gekko gecko* (Linnaeus, 1758). With only four specimens, *Cylindrophis boulengeri* Roux, 1911, is the best represented species. No amphibian species are represented in the collection.

**Table 15.** Overview of the East Timor amphibian and reptile collections of IICT.

Family	Genus	Species	Localities – Accession number	References	Number of specimens
			(* denotes a type specimen)		
**REPTILES**					
**SQUAMATA** Oppel, 1811					
**Agamidae** Gray, 1827	*Draco* Linnaeus, 1758	*Draco timorensis* Kuhl, 1820	Dili – IICT/R 6-1956, Timor3	Manaças (1956, 1972)	2
**Gekkonidae** Gray, 1825	*Gekko* Laurenti, 1768	*Gekko gecko* (Linnaeus, 1758)	Dili – IICT/R 4/1956, Timor6	Manaças (1956, 1972)	2
	*Hemidactylus* Gray, 1825	*Hemidactylus frenatus* Duméril & Bibron, 1836	Dili – IICT/R 1-3/1956	Manaças (1972)	3
**Varanidae** Hardwicke & Gray, 1824	*Varanus* Merrem, 1820	*Varanus timorensis* (Gray, 1831)	Dili – IICT/R 7/1956	Manaças (1972)	1
SERPENTES					
**Colubridae** Oppel, 1811	*Dendrelaphis* Boulenger, 1890	*Dendrelaphis inornatus timorensis* Smith, 1927	Dili – IICT/R Timor2, Timor5		2
**Elapidae** Boie, 1827	*Laticauda* Laurenti, 1768	*Laticauda colubrina* (Schneider, 1799)	Dili – IICT/R Timor4		1
**Viperidae** Oppel, 1811	*Trimeresurus* Lacepede, 1804	*Trimeresurus insularis* Kramer, 1977	Dili – IICT/R 8/1956	Manaças (1972)	1
**Cylindrophiidae** Fitzinger, 1843	*Cylindrophis* Wagler, 1828	*Cylindrophis boulengeri* Roux, 1911	Dili – IICT/R 9-10/1956, Timor1, Timor8		4
**Typhlopidae** Merrem, 1820	*Indotyphlops* Hedges, Marion, Lipp, Marin & Vidal, 2014	*Indotyphlops braminus* (Daudin, 1803)	Dili – IICT/R Timor7		1
**TOTAL NUMBER OF REPTILE SPECIMENS**					**17**

**Figure 16.**
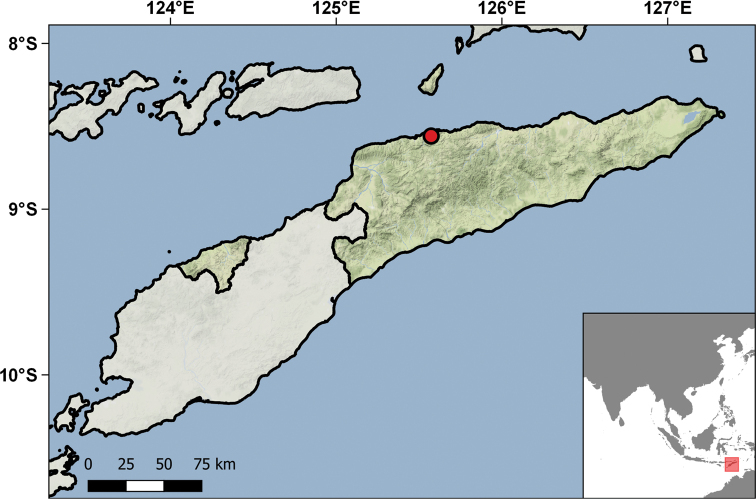
Distribution of the East Timor localities represented in the IICT herpetological collections.

Geographically, all specimens were collected in Dili (Table 16; Fig. 16). While a few specimens were collected in 1953 and sent to the CZL by the Portuguese anthropologist Ruy Cinatti (1915–1986), most of the material was collected and offered by Cunha Porto (birth and death dates unknown) in 1956. This small collection was only studied by Sara Manaças and published on twice (Manaças 1956, 1972).

**Table 16.** Gazetteer of East Timor localities of IICT specimens. Latitude and longitude decimal coordinates are presented in WGS-84 projection.

State	Verbatim locality	Current locality	Latitude and Longitude	Uncertainty (meters)	Elevation (meters)	Number of taxa/records
Dili	Dili	Dili	-8.558611, 125.573611	6710	3	8/14

Herpetological specimens from East Timor are rare in museum collections, with recent surveys contributing relevant collections for MCZ, with 256 specimens, and the NCSM, with 30 specimens (data retrieved from GBIF.org in February 2021). Material from other collections worldwide generally do not exceed 10 specimens (data retrieved from GBIF.org in February 2021). There is also a small collection with 23 specimens at the MCUC (Themido 1941).

The dataset of this collection is available at GBIF (Ceríaco and Marques 2018e; https://www.gbif.org/dataset/7df6557f-1996-4874-a08a-1ac5718ef413).

## Discussion

### The importance of the IICT herpetological collection for research and conservation

As part of an institution aimed at the scientific study of the Portuguese colonial territories, the CZL and its researchers had privileged access to the fauna of those areas. This resulted in very important herpetological collections, which rank amongst the largest available collections for some of the Portuguese speaking countries in Africa. Within the Portuguese natural history context, the IICT herpetological collections rank amongst the largest of the country.

Besides its overall diversity and extensive geographic distribution (297 species from 258 unique localities in eight countries), the collections house a considerable amount of type material. This type material belongs to nine different taxa, mainly from São Tomé & Príncipe (18 types), Angola (25 types), and Mozambique (three types).

As demonstrated by the role of the IICT specimens in the description of new species (Ceríaco 2015; Ceríaco et al. 2016, 2017, 2020a, 2021a; Soares et al. 2018; Hallermannn et al. 2020), national checklists and atlases (Ceríaco et al. 2018a; Marques et al. 2018), as well as phylogeographic studies (Ceríaco et al. 2020c), the collections housed in the IICT continue to play an important role on the development of herpetology in a global context, especially in the Portuguese speaking world. This is critically important, as this collection, originally created in a colonial context, can now serve to enhance and foster scientific cooperation and knowledge transfer between former colonial powers and independent countries.

Currently, the collection is fully catalogued and accessible. It is regularly consulted by international researchers, Portuguese and foreign students for their Master and PhD theses, and used in conservation assessments (Ceríaco and Marques 2019).

### The future of Portuguese natural history collections

Natural history collections are an irreplaceable resource for the study of past and present biodiversity and its future conservation, as well as an invaluable resource for teaching and training students (Miller et al. 2020). The recent global pandemic has brought attention to how these collections can be used in multidisciplinary studies such as helping to understand emerging diseases (Cook et al. 2020).

Despite their scientific importance and potential use for research, biodiversity conservation, and teaching, natural history collections currently face serious challenges (Kemp 2015). These challenges vary from country to country due to specific economic and political situations and different institutional backgrounds. This is particularly true for southern European countries whose funds for research are not comparable to their northern European and North American counterparts. Recently, Italian researchers pointed out the severe risk of the neglect and loss of the country’s natural history collections, due to severe disinvestment and lack of proper and sustainable management strategies (Andreone et al. 2014; Andreone 2015). Although perhaps not as dramatic as the Italian scenario, the Portuguese situation, for which the IICT collections serve as an example, is far from ideal.

Altogether, the Portuguese natural history collections house more than 3,500,000 specimens. Although this number may seem small when compared to other major natural history collections in Europe, the Portuguese natural history collections are rich in specimens from biodiversity hotspots, such as the Mediterranean region and the tropical regions spanned by their former colonial possessions and range from the mid-eighteenth century to the present day. As recently noted by Monfils et al. (2020), smaller, regional collections play a fundamental role in modern biodiversity research and conservation, comparable to those of larger museums. This is especially true for the IICT collections, which represent, for some African countries, the largest (or among the largest) herpetological collections available.

Despite its national and international importance, the staff allocated to the curation, preservation, and study of this collection is limited. None of the three main museums, MUHNAC, MHNC-UP, and MCUC, have collection managers as part of their permanent staff, and very few collections have permanent curators. The curatorial staff in the three main museums is composed of movable groups of volunteers, including graduate students, post-doctoral researchers, grantees from different research projects, and retired professors or researchers at other national institutions.

The major Portuguese natural history collections are currently part of larger interdisciplinary university museums, which were recently created through the merging of former more discipline-oriented museums. The University of Lisbon manages MUHNAC, which houses zoological, botanical (herbaria), geological, paleontological, anthropological, and scientific instrument collections, as well as an assortment of memorabilia and smaller collections related to the history of science in the university. The IICT collections, despite being institutionally independent from the museum, are in practice managed as part of the museum collections, as they share the same space and staff. Similarly, the University of Porto and the University of Coimbra, respectively, manage MHNC-UP and MCUC, which also house comparably diverse and interdisciplinary collections from the historical museums of both universities. With the exception of the Herbarium of the University of Coimbra (COI; the largest Portuguese herbarium, with ca. 800.000 specimens) which is run by the Department of Life Sciences, these interdisciplinary museums are all directly under the management of their respective dean’s offices. Other collections, usually run by research groups in departments, research centres, or municipalities, also exist, but these are usually smaller in number of specimens and have very limited taxonomic scope.

There are various reasons why these disciplinarily distinct collections were merged within the university museums structure, although one of the major drivers was the economic and management burden caused by having several independent museums within the universities. There are both advantages and disadvantages to such mergers, which have raised several challenges at the methodological, management, and even epistemological levels. Curating a biological collection is radically different from curating a collection of historical scientific instruments and developing a functional database that serves both the interests and needs of curators of almost opposite typologies of collections is challenging.

Being a university museum is, *a priori*, a very interesting opportunity in favour of natural history collections, as this institutional relation can foster important research collaborations and teaching partnerships between the museum and the rest of the academic community, from professors and researchers to graduate and undergraduate students (Cook et al. 2014). Some of the larger and more important natural history collections in the USA are part of universities, as it is the case of the MCZ, MVZ, YPM, or the natural history museums of the universities of Kansas, Michigan, and Florida, and their collections are used on a daily basis by the university community, as well as by national and international researchers. Contrary to this advantageous relationship between natural history collections and universities, the Portuguese case has produced different outcomes. Coming from decades of abandonment, these collections are generally perceived by the academic community as the dusty remains of the past practices of science, cumbersome to manage and use, and mostly oriented to the low-impact factor science of taxonomy. Merging it with other types of museum material, such as old scientific instruments and academic memorabilia, has contributed to reinforce the idea of museums as repositories of historical heritage, time capsules of the science of the past (Lourenço and Dias 2017), rather than tools of modern and impactful research.

This association is pernicious and has consequences for the relationship between the museum and its academic community. Firstly, it has led to a physical and emotional separation of professors, researchers, and students from the museum. This has resulted in several immediate problems, such as the abandonment of systematic and taxonomic studies associated with the collections, fostering the already worrisome distance between taxonomists and the rest of the academic community and the well-known negative consequences that this has for biodiversity studies as a whole (Britz et al. 2020). The lack of continuity in the use of collections and the transmission of collections-related practices has led to the loss of basic curatorial and natural history competencies by the community, such as specimen collecting, fixation, and taxidermic techniques, natural history collection management, etc. Although specimen collecting remains an essential tool for biological research (Rocha et al. 2014) and enriching collections is still fundamental to keeping natural history collections relevant for future research (Hope et al. 2018), Portuguese natural history collections are experiencing a considerable deceleration in accessioning new specimens, with some natural history collections not having incorporated any newly collected specimens in the last two decades.

Divorced from its research and teaching objectives, the collection staff is usually reduced to a minimum, which has immediate consequences for the curation and maintenance of its collections, including cataloguing and digitising, leading to drastic limitations on accessibility. This lack of accessibility contributes to a taxonomic impediment (Coleman 2015), frustrating the users of these collections and impeding, rather than supporting, research. This situation further confirms the current general idea of the museum as a dusty place, incapable of supporting modern research, creating a vicious circle. Unfortunately, these consequences have far-reaching effects on a global scale. As a considerable percentage of natural history specimens housed in Portuguese natural history collections originated in the former Portuguese colonial territories in South America, Africa and Asia, all of them important biodiversity hotspots but currently suffering from major threats to biodiversity, the lack of research in and accessibility to these collections is a major challenge for international researchers, particularly those in these megadiverse and developing countries (Romeiras et al. 2014; Neves et al. 2018; Ceríaco and Marques 2019) .

Natural history collections in Portuguese universities risk being considered simply as the historical heritage of the universities, becoming displays of past glories, mostly used in commemorative and outreach programs. This historical nature also causes some practical challenges in terms of their use in exhibitions other than those more focused on history. As many of these collections were assembled for research purposes from the mid-eighteenth century to the mid-twentieth century, their aesthetic and pedagogic value for exhibitions is limited. The concerns raised by the natural history collections being considered as historical heritage also causes problems regarding the accessibility and use of collections. As the first author personally experienced, researchers are sometimes blocked from the study of fluid preserved specimens simply because this would mean that the jar containing those specimens would have to be opened and that would destroy its historical sealant.

Recently, Portuguese natural history collections have been included in important international initiatives aimed at promoting their use, such as DiSSCo, a pan-European Research Infrastructure (Addink et al. 2019). This is a critical opportunity to review current practices and strategies. Natural history collections, although an important part of the historical scientific heritage of Portuguese universities, need to be acknowledged for their full potential and scientific importance. In the current biodiversity crisis, natural history collections should not be seen, managed, and funded at the same level as academic memorabilia or historical scientific instruments (notwithstanding their importance), but as fundamental tools for the study of the earth’s biota and its conservation. In order to achieve this, a new generation of museum naturalists needs to be trained and promoted.

Interestingly, the problems surrounding the Portuguese natural history museums are not new but are a recurrent situation. In 1865, the Portuguese herpetologist José Vicente Barbosa du Bocage (1823–1907), then director of the Zoological Section of the National Museum of Lisbon (precursor of current day MUHNAC), wrote a brief report about the state of the museum (Bocage 1865). The following statement, transcribed from Bocage’s (1865) mid-nineteenth century report, still applies:

"*In the current conditions, with the present organisation, the Lisbon museum not only is unable to develop and prosper but will shortly precipitate into a fast and lethal decadence. There is no staff to study part of its collections, there is no staff dealing with its conservation, there is no staff to prepare a portion of its specimens, there are no resources that can be used to enrich its collections. What more can I do besides ask for immediate help? There may be people amongst us that consider this establishment useless, and this may even be the most predominant feeling in the country; but in this case, logic demands that it cannot remain in its current conditions, but rather needs to be closed. To have or to not have a zoological museum is the first thing that needs to be decided, but if we decide to have one, the triumph of the status quo can’t be the corollary of the needed actions.*"

### A protocol to rescue abandoned natural history collections

After decades of being in limbo, world natural history collections are currently in a state of flux. Several major international initiatives, such as iDigBio (www.idigbio.org) in the USA and DiSSCo (http://www.dissco.eu) in Europe, are driving an important and almost revolutionary change on how society sees, uses, and values these resources. This revolution aims to foster the use of natural history collections data through digitisation and global accessibility (Hendrick et al. 2020). Modern technologies allow us to revisit specimens in ways that seemed like science fiction just a few years ago, extending the specimens beyond their traditional use and adding new layers of information (Webster 2018).

Yet not all collections present the minimum standards for joining the ongoing revolution, especially abandoned collections. Due to lack of proper curation, lack of accessibility, inconsistent internal organisation, and poor conservation, these abandoned collections risk being left aside, simply because in their current state they are almost unusable. This is not only a problem for the collections but a loss of critical scientific data. Unfortunately, this situation is not exclusive to the IICT collections; several collections around the world are in a similar or worse state.

**Figure 17.**
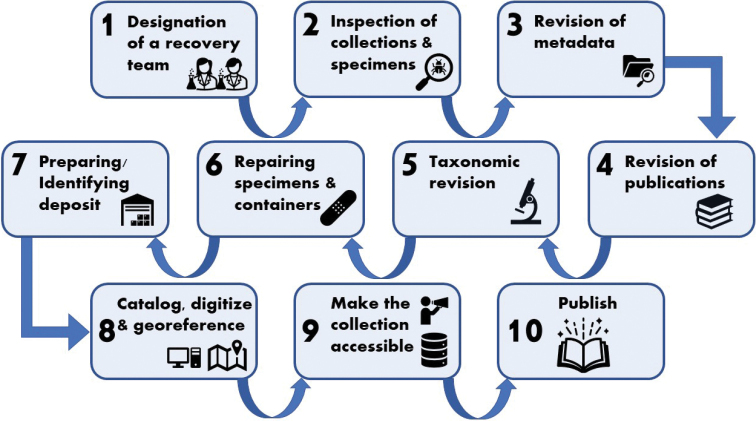
Ten steps workflow for recovering abandoned collections.

Based on our experience with the recovery of the IICT herpetological collection, we have developed a simplified workflow of ten steps (Fig. 17) for dealing with abandoned collections. For the purpose of this protocol, we assume that the legal ownership of the collection is clarified (i.e., that the collection has a legal owner), and it is accessioned in an institution. In the cases for which the legal ownership is not known, this should be addressed first.

1. Designation of a recovery team: The recovery team should be constituted by experts on the taxa (taxonomists) represented in the collection, collection managers or individuals trained in natural history curatorial practices, and volunteers or students. The presence of experts on conservation and restoration is desirable but depends on the particular collection. Health inspectors and other appropriate authorities should be called to assess potential dangers and hazards that the collection may pose to public health and security.
2. Inspection of the collection and specimens: An initial inspection of the collection allows the team to understand the challenge that lies ahead. During this inspection the team should record the number of specimens in the collection, identify the main conservation techniques used (taxidermy, osteological preparation, fluid preservation, etc.) and the main problems affecting the collection (e.g., pests, loss of preservative fluids, environmental problems in the storage area). Estimate how many specimens will need more complete intervention, and which materials, chemicals, and other gear are needed to proceed. The team should gather all available catalogues, logs of databases, field notebooks, and other documentation associated with the collection. The team should evaluate whether the place where the collection is housed has sufficient space and laboratory facilities to safely carry out the recovery procedures.
3. Revision of the available metadata: It is fundamental that all the available data associated with the collection and specimens be gathered and reviewed. Without this data, specimens will lose their scientific importance. This associated metadata may be in a variety of formats such as catalogues, databases, field notebooks, tags associated with the specimens, labels on containers or specimens, manuscript documentation, or data from studies of the specimens. This metadata should never be dissociated from the specimen, even if the team finds it to be outdated or not entirely correct. All metadata, if possible, should be digitised. Field notebooks and old catalogues can be digitised and converted to pdf format, while old labels can be digitally photographed. Original documentation should be kept and deposited in an appropriate place that is accessible to researchers, curators, and collection managers.
4. Review of available published sources: Many collections were studied and published on by researchers in the past. These publications often have additional data regarding the collections or specimens that should, whenever possible, be cross-linked with the specimens in the database. Publications related to the collection vary in format and content: they may be books or papers in scientific journals, and can provide more details on the locality, morphology, or nomenclatural type status of the specimen, etc. Specimen tissues may have sequences deposited in databases such as GenBank (www.genbank.org) or specimens may have been CT-Scanned, radiographed, or photographed and have morphological data available in morphology databases such as MorphoSource (www.morphosource.com) or Morphobank (www.morphobank.org).
5. Taxonomic revision of individual specimens: All specimens should be individually inspected by a trained taxonomist to review the available identification and confirm, correct, or update it as necessary. The identification should take into account the associated metadata and all published sources on the specimen. Identification techniques vary across taxonomic groups, thus the need for a trained taxonomist. New labels should be created and associated with the specimen, but always keep all the original labels.
6. Repairing specimens and replacing containers: As each specimen is individually examined for taxonomic purposes, the team will be able to evaluate the condition of the specimen and container. Some specimens will require intervention (e.g., rehydration, cleaning, consolidation of taxidermy mounts, etc.), and some will have problems with their containers (broken containers, evaporation problems, etc.), which can either be repaired or replaced with new containers. For fluid preserved specimens see Simmons (2014), for taxidermy see Ramotnik (2006), for entomological collections see Robinson (2008). The Society for Preservation of Natural History Collections (SPNHC; www.spnhc.org) has online resources regarding this topic.
7. Preparing or identifying a new storage location: In most instances abandoned collections are kept in insalubrious or inadequate repositories. This is one of the major drivers of collection problems. The team should evaluate the current storage area, determine whether the environmental conditions are appropriate for the collection, if cleaning or adaptations are required, or if it is preferable to move the collection to new repository. For a better understanding of the management and conditions for collection storage, see Elkin and Norris (2019).
8. Cataloguing, digitisation, and georeferencing protocols: If the collection is not catalogued (i.e., if the specimens do not have a unique identifier), each specimen should be assigned an individual catalogue number. All the data gathered and reviewed during this process should be digitised and formatted according to standard formats, such as DarwinCore (http://dwc.tdwg.org), in order to follow the FAIR standards (Wilkinson et al. 2016) and be shared in major international databases, such as GBIF (www.gbif.org). Whenever possible, locality data should be georeferenced using established protocols (see Chapman and Wieczorek 2020).
9. Make the collection accessible to the public: Whenever the recovery process is finished, the collection should be made accessible to the public, both physically and virtually through the museum databases and the publication of the datasets on GBIF (www.gbif.org). This will ensure that the collection is visible and usable by any interested parties. A usable and useful collection is the best insurance against future abandonment.
10. Publish: The team should publish the results of the recovery process, including any taxonomic or scientifically relevant findings they make.
